# Click to Translate: Synthesis of *Trans*‐Cyclooctene Modified 5′ mRNA Caps for Bioorthogonal Activation

**DOI:** 10.1002/cbic.70420

**Published:** 2026-06-13

**Authors:** Niclas Zips, Ekaterina Kulko, Stephanie Kath‐Schorr

**Affiliations:** ^1^ Department of Chemistry and Biochemistry University of Cologne Köln Germany

**Keywords:** 5′‐Cap, click‐to‐release, cyclooctene, mRNA, mRNA translation

## Abstract

mRNA translation can be controlled through chemical modification of the 5′‐Cap, yet systematic insights into structure–reactivity relationships of bioorthogonally activatable Cap analogues remain limited. Here, we report the synthesis of *trans*‐cyclooctene (TCO)‐modified 5′ mRNA caps (ZipCaps) and a comparative analysis of distinct TCO isomers. A controlled synthetic strategy enabled access to axial and equatorial derivatives, revealing stereochemical effects on click‐to‐release reactivity and stability. ZipCap‐modified mRNA exhibits reduced translation that can be restored upon tetrazine addition, enabling reversible chemical control of protein expression in cells. In contrast to a recent report describing complete translational suppression, residual translation is observed, highlighting the sensitivity of Cap‐dependent translation to structural variations.

## Graphical abstract

*Tr* *ans*‐cyclooctene‐modified 5′‐Cap analogues provide a platform for bioorthogonal modulation of mRNA translation. A controlled synthetic strategy enabled access to functionalized Caps that attenuate translation and permit partial restoration upon tetrazine addition. This approach offers a basis for externally triggered regulation of protein expression and future development of spatiotemporal control over mRNA function in cells.

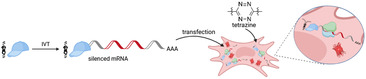

## 1 Introduction

Messenger RNA (mRNA) has emerged as a central platform in modern therapeutics [1]. However, precise spatial and temporal control over its translation remains a significant challenge. Such control would enable modulation of mRNA pharmacokinetics by restricting protein expression to defined sites or time windows, thereby minimizing off‐target effects and improving safety profiles [2]. Moreover, tools that permit on‐demand activation of mRNA translation are highly valuable for fundamental research, particularly for probing dynamic cellular processes with high spatiotemporal resolution [3]. Labeling of mRNAs has thus far primarily been achieved at terminal positions, either at the *N* ^2^‐position of the 5′‐Cap guanosine, as demonstrated by Rentmeister and coworkers, or at the 3′ end via the poly(A) tail [4]. In contrast, internal incorporation of reactive handles has been realized through the use of unnatural base pairs, as established in our previous work [5, 6]. In 2022, Rentmeister and co‐workers addressed the challenge to control mRNA translation by developing photocaged 5′‐Cap analogues in which the *N* ^2^‐position of guanosine is modified, enabling UV‐light‐induced activation of mRNA translation [4]. This modification inhibits binding to the eukaryotic translation initiation factor eIF4E, thereby suppressing translation. Although this strategy was demonstrated to function in vivo, including in zebrafish models [7], the limited tissue penetration of UV light restricts its applicability in mammalian systems and, in particular, in humans. To overcome this limitation, the approach presented here integrates translational silencing with the bioorthogonal click‐to‐release concept developed by Robillard and coworkers [8]. This approach relies on two complementary components: a *trans*‐cyclooctene (TCO) and a tetrazine trigger. While mRNA is typically delivered in vivo using lipid nanoparticles [9], previous work has shown that click‐to‐release reactions can also be achieved in vivo following systemic intravenous administration of tetrazines in large excess [10, 11]. More recent studies demonstrated that selective localization of tetrazines can be achieved through tetrazine‐functionalized antibody‐drug conjugates [12] or tetrazine‐conjugated tumor‐targeting diabodies [13], thereby restricting the click reaction predominantly to the desired site.

In this study, we report a comprehensive synthesis and systematic evaluation of TCO‐modified 5′‐Cap analogues. By accessing distinct TCO isomers, we directly compare axial and equatorial configurations with respect to their click‐to‐release performance. This analysis reveals pronounced stereochemical control over release kinetics and efficiency, consistent with established behavior of TCO systems [8, 14] and extends these structure–reactivity relationships to 5′‐Cap‐modified mRNA. By combining TCO‐caged mRNA with spatially controlled tetrazine delivery, selective activation of mRNA translation at defined tissue sites becomes feasible. During preparation of this manuscript, Rentmeister and coworkers reported a related concept using a different reporter system [15]. Under partially comparable experimental conditions, however, we observed a divergent outcome. Our results therefore complement these findings and additional investigations will be required to resolve the origin of this discrepancy. Notably, the present work places particular emphasis on the underlying synthetic strategy, providing alternative synthetic routes and identifying aspects that remain unresolved. We term this platform ZipCaps, reflecting its modular design and potential for site‐specific activation of mRNA translation.

**FIGURE 1:**
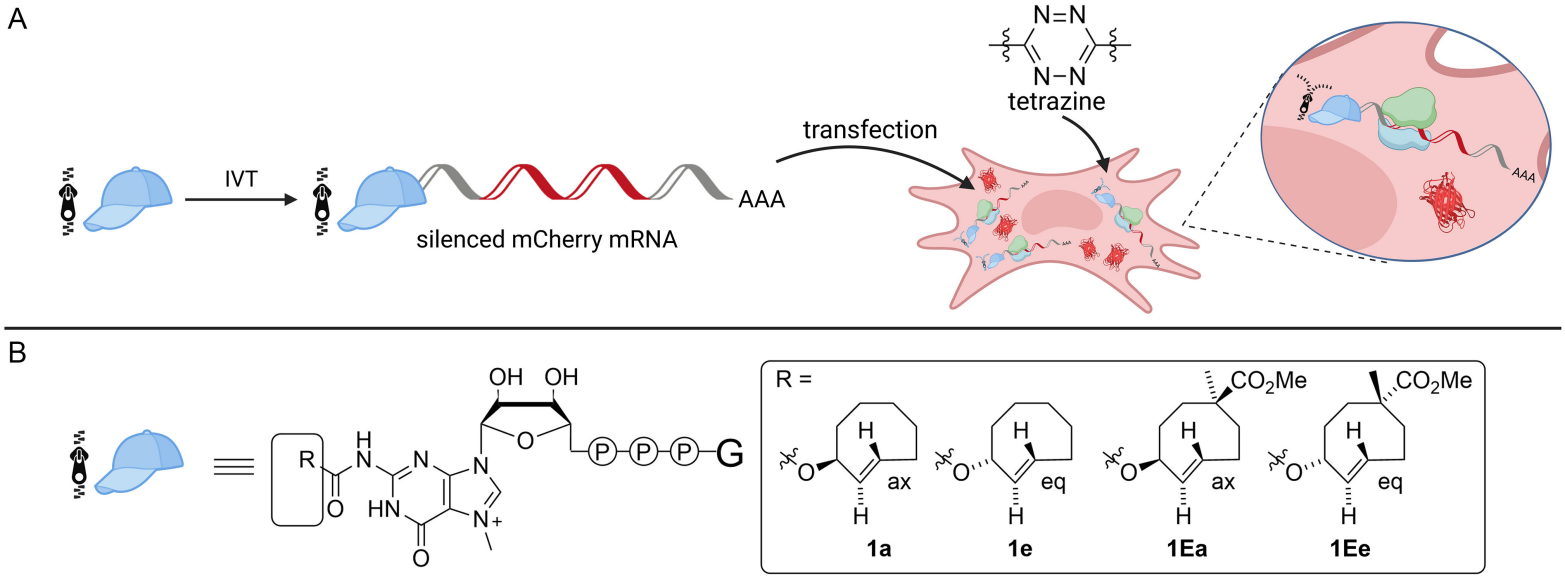
(A) Schematic representation of the general concept. A TCO‐functionalized Cap0 [16] analogue is incorporated into *mCherry* mRNA via in vitro transcription (IVT), yielding translationally inactive mRNA. Following cellular transfection, addition of a cell‐permeable tetrazine triggers click‐to‐release decaging, restoring the native Cap0 [16] structure and thereby translational competence. (B) Overview of the synthetic targets. ZipCap **1a** and **1e** differ in the orientation of the hydroxy group relative to the ring plane, adopting axial and equatorial configurations, respectively. An analogous relationship applies to **1Ea** and **1Ee**. Created in BioRender. Kath‐Schorr, S. (2026) https://BioRender.com/rvy9td2.

ZipCap **1a** was identified as the main target structure (Figure 1B), whereas the ester‐functionalized ZipCap **1Ea** was of particular interest due to the potential for further derivatization at the ester moiety. As isomerization of a functionalized *cis*‐cyclooctene (CCO) affords both axial (ax) and equatorial (eq) TCO isomers, ZipCaps containing the equatorial isomers were synthesized alongside their axial counterparts to enable direct comparison across systems. While axial TCO derivatives are known to exhibit significantly faster click‐to‐release kinetics [8, 17], attributed to increased steric accessibility of the strained double bond, inclusion of both isomers allows systematic evaluation of their impact in the context of 5′‐Cap‐modified mRNA.

## 2 Results and Discussion

### 2.1 Synthesis of TCO‐ZipCaps

**SCHEME 1:**
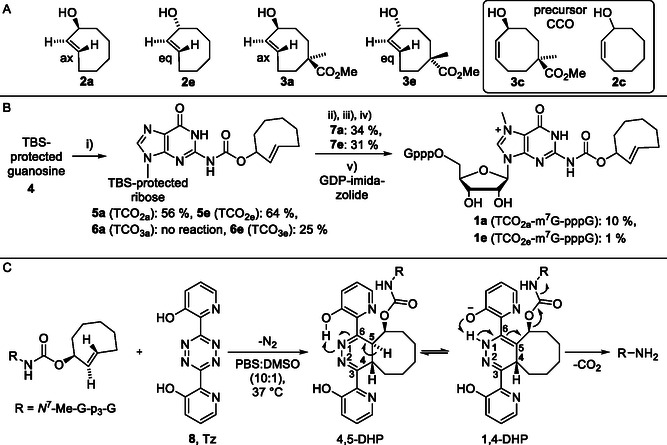
(A) TCO derivatives synthesized in this study. (B) Synthetic route towards TCO‐functionalized 5′ mRNA Cap analogues: i) COCl_2_,(20% in toluene), pyridine, dichloromethane, ii) methyl iodide, DMF, iii) TBAF, THF, iv) POCl_3_, trimethylphosphate, v) guanosine diphosphate imidazolide, ZnCl_2_, DMF, (C) Click‐to‐release mechanism of the TCO‐functionalized Cap. Following inverse electron demand Diels–Alder reaction and subsequent retro Diels–Alder reaction, a 4,5‐dihydropyridazine (DHP) intermediate is formed. Tautomerization to the corresponding 1,4‐DHP enables release of the caging group.

To enable installation of a bioorthogonally cleavable moiety at the 5′‐Cap, TCO derivatives bearing an allylic hydroxy group were required for subsequent click‐to‐release activation. The corresponding TCOs **2a**, **2e**, **3a**, and **3e** are shown in Scheme 1A and were obtained via flow photoisomerization from the respective CCO precursors **2c** [8] and **3c** [14].

Literature protocols for functionalization of the *N* ^2^‐position of guanosine via a carbamate linkage typically employ a one‐pot procedure using phosgene for isocyanate formation in the presence of excess alcohol and trimethylsilyl chloride for ribose protection [4]. While suitable for readily available alcohols, this approach is less practical for TCO derivatives, which are synthetically demanding and costly, often requiring flow chemistry [18, 19]. Minimization of reagent excess is therefore essential. To enable more controlled isocyanate formation and reduce TCO consumption, trimethylsilyl protection was replaced with the more robust *tert*‐butyldimethylsilyl (TBS) protecting group [4].

Following phosgene‐mediated activation at the *N* ^2^‐position, TCO derivatives **2a**, **2e**, and **3e** were coupled to TBS‐protected guanosine **4** using only 1.5 equivalents of the respective alcohol, affording TCO‐modified guanosine intermediates **5a**, **5e**, and **6e** in moderate to good yields (Scheme 1B). In contrast, TCO **3a** showed no conversion to **6a**, as NMR analysis indicated exclusive formation of the *cis*‐cyclooctene product, consistent with the known instability and rapid re‐isomerization of this derivative [14].

Subsequent *N* ^7^ methylation of **5a** and **5e** using excess methyl iodide, followed by TBS deprotection with tetrabutylammonium fluoride (TBAF), afforded the corresponding intermediates. Partial desilylation occurred during methylation, enabling complete deprotection with only 2.5 equivalents of TBAF. Subsequent 5′ monophosphorylation yielded **7a** and **7e** in 34% and 31% over three steps, respectively (Sections S2.4 and S2.6). Compared to previously reported procedures, this controlled strategy provides improved overall yields while significantly reducing TCO consumption [4, 15].

**FIGURE 2:**
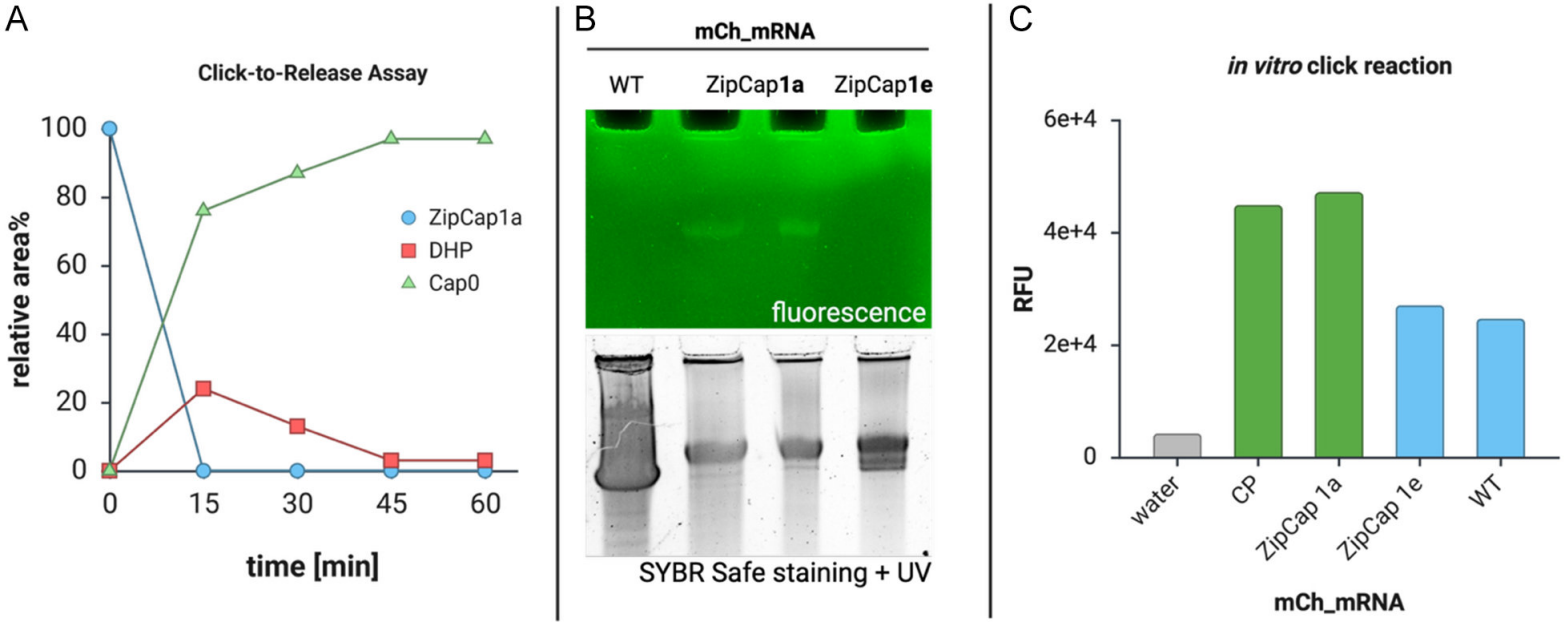
(A) Time‐dependent release of Cap0 from ZipCap **1a** after adding tetrazine **8**. After 15 min, no remaining ZipCap **1a** was detectable and 76% of Cap0 was formed, with the remaining fraction corresponding to DHP intermediates (Diels–Alder adducts). The release increased to 87% after 30 min, leaving ≈13% DHP. After 45 min, 97% of Cap0 was formed, with no further increase observed at 60 min, most likely due to formation of the nonreleasing 2,5‐DHP tautomer. (B) PAGE analysis of the incorporation of ZipCap **1a** and **1e** into mCherry mRNA. Wild‐type (WT) mCherry mRNA without modifications (**mCh_mRNA_WT**) [6] was used as a negative control (Section S1.2). ZipCap **1a** was synthesized twice independently and two different batches were analyzed**.** From left to right: WT, ZipCap **1a** (middle left: synthesis 1, middle right: synthesis 2), and lastly ZipCap **1e**. The upper panel shows fluorescence detection, while the lower panel displays SYBR Safe staining after UV illumination. (C) Fluorescence readout of the in vitro click‐to‐release reaction between **mCh_mRNA_ZipCap‐1a**/**1e** and a tetrazine‐conjugated fluorophore (**Tz‐FL**, Section S1.2). mCherry mRNA containing a cyclopropene moiety (**mCh_mRNA_CP**) served as a positive control [5, 6], while unmodified mRNA (**mCh_mRNA_WT**) was used as a negative control. The solution containing mRNA bearing ZipCap **1a** exhibited a strong fluorescent signal comparable to the positive control, indicating successful click reaction with **Tz‐FL**. In contrast, no detectable fluorescence was observed for the solution containing mRNA with ZipCap 1e, suggesting that this analogue does not efficiently undergo the click reaction under the tested conditions or is unstable in aqueous solution. Created in BioRender. Kath‐Schorr, S. (2026) https://BioRender.com/5sepuv3.

Coupling to imidazolide‐activated guanosine diphosphate under Lewis acidic conditions afforded Cap analogues **1a** and **1e**. Purification was achieved by C18 flash chromatography followed by preparative high‐performance liquid chromatography using a hydrophilic interaction liquid chromatography [20] column, providing the desired Cap analogues in high purity (Sections S2.8 and S2.9) and in 10% and 1% yield, respectively. Notably, equatorial derivatives **7e** and **1e** exhibited reduced stability in aqueous solution, as indicated by both the ^1^H NMR analysis of **7e** (Figures S5 and S6) and polyacrylamide gel electrophoresis (PAGE) analysis of mRNA containing **1e** as a Cap structure (Figure 2B). The PAGE results are discussed in more detail below.

### 2.2 Evaluation of Click‐to‐Release Reactivity and Stability

Building on the successful synthesis of ZipCaps, the reactivity of ZipCap **1a** was evaluated in a click‐to‐release assay using equimolar amounts of tetrazine **8** and **1a**. Reaction progress was monitored by liquid chromatography‐mass spectrometry (LC‐MS), employing a mixture of di‐ and triphosphate linked species (Section S4). In contrast to sulfonamide‐functionalized tetrazines, tetrazine **8** is synthetically more readily available while simultaneously offering moderate Caco‐2 values (Section S3). Therefore, **8** was selected as a compromise between reactivity, cellular accessibility, and synthetic tractability [21, 22].

In the in vitro click‐to‐release assay (Figure 2A), **1a** was fully consumed within 15 min, accompanied by the release of 76% Cap0. Cap0 release further increased over time, reaching a plateau of 97% after 45 min. The residual DHP is most likely attributable to formation of the nonreleasing 2,5‐DHP tautomer. The stability of **1a** was subsequently evaluated under conditions mimicking IVT conditions, both in the presence and absence of dithiothreitol, a common additive used to enhance yields during IVT. Under these conditions, **1a** remained intact over 2 h (Section S4.2).

### 2.3 In Vitro Synthesis of Capped mRNAs

To generate mRNAs bearing different 5′‐Cap structures, IVT was carried out using T7 RNA polymerase. As a model system, a mRNA encoding the *mCherry* reporter protein was selected. The corresponding double‐stranded DNA (dsDNA) template (**mCh_DNA**) was generated by polymerase chain reaction amplification from the *pmCherry‐N1* plasmid (Section S1.2). IVT reactions were performed in the presence of different Cap analogues followed by poly(A) tailing using *E. Coli* Poly(A) Polymerase, yielding Cap0‐capped [16] (**mCh_mRNA_Cap0**), ZipCap **1a**‐capped (**mCh_mRNA_ZipCap‐1a)**, ZipCap **1e**‐capped (**mCh_mRNA_ZipCap‐1e**), and uncapped (**mCh_mRNA_uncap**) transcripts. The integrity and full length of the resulting mRNA products were confirmed by agarose gel electrophoresis (Section S1.3). In addition, the transcription yield and capping efficiency of ZipCap **1a** were assessed in direct comparison with Cap0 and ARCA. ZipCap **1a** showed transcription performance and capping efficiencies comparable to those obtained with the established Cap analogues (see Section S1.2). Incorporation of ZipCaps into mRNA was assessed by PAGE followed by an *in‐gel* click reaction between the TCO moiety and a tetrazine‐conjugated fluorophore (**Tz‐FL**) to observe fluorescence turn‐on upon successful click reaction. WT mRNA was used as a negative control (Figure 2B). A distinct fluorescent signal was observed exclusively for **mCh_mRNA_ZipCap‐1a**, whereas no signal was detected for the corresponding equatorial analogue **mCh_mRNA_ZipCap‐1e**. This indicates reduced stability of the TCO carbamate linkage in the equatorial configuration under aqueous conditions, which is in agreement with the instability observed for the monophosphate precursor **7e**. Notably, ZipCap **1a** obtained from two independent syntheses was used (Figure 2B, middle left: synthesis 1; middle right: synthesis 2). These observations further align with the in vitro click‐to‐release experiments employing a tetrazine‐conjugated fluorophore and fluorescence‐based plate assay readout (Figure 2C). mRNA containing a cyclopropene moiety (**mCh_mRNA_CP**) served as a positive control [5, 6] and WT mRNA as a negative control. Notably, fluorescence was observed only for samples containing **mCh_mRNA_ZipCap‐1a,** indicating successful reaction of the strained alkene‐equipped mRNA with **Tz‐FL** and subsequent activation of the fluorophore. In contrast, no detectable fluorescence was observed for **mCh_mRNA_ZipCap‐1e**, further supporting the reduced stability of the equatorial isomer under the applied conditions. Accordingly, **mCh_mRNA_ZipCap‐1a** was selected for subsequent biological evaluation in cells.

### 2.4 Cellular Evaluation of ZipCap‐Mediated Control of Translation

**FIGURE 3:**
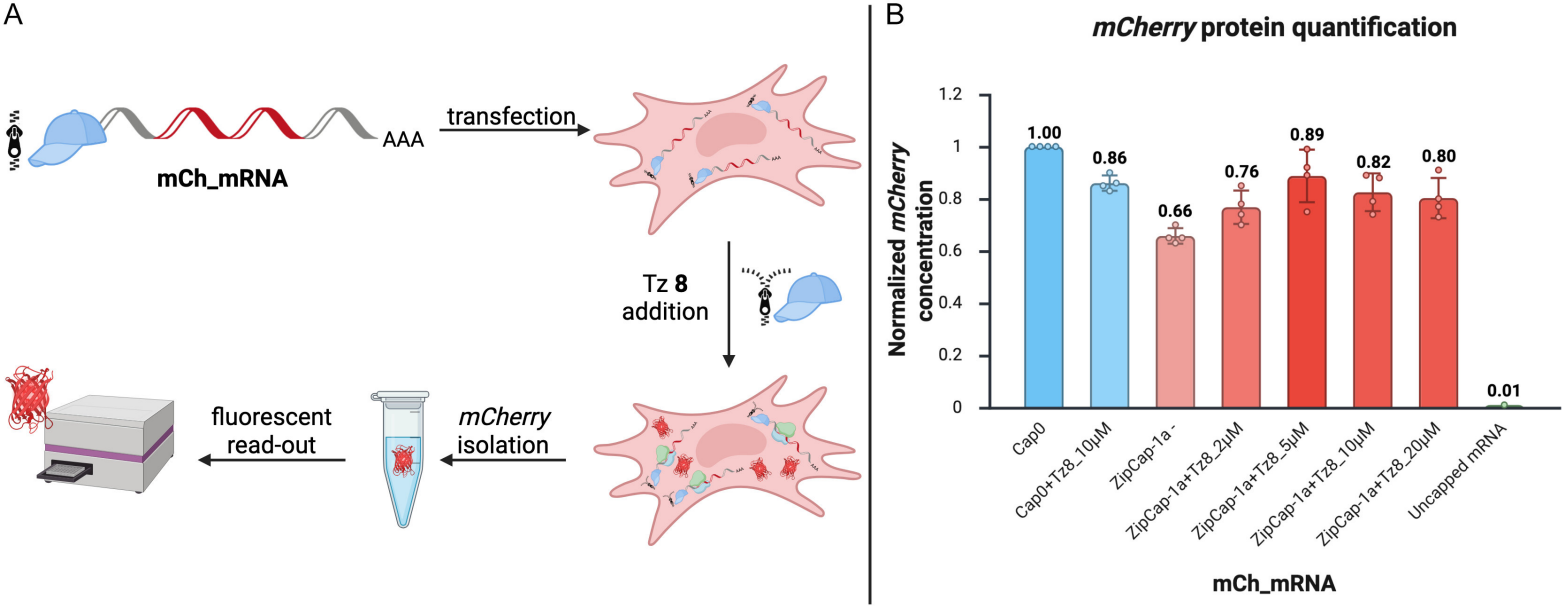
Evaluation of ZipCap‐mediated translational modulation. (A) Schematic illustration for the workflow for quantification of *mCherry* protein expression. Seeded cells were transfected with differently capped **mCh_mRNA** or uncapped mRNA. Tetrazine (Tz, **8**) was added 4 h post‐transfection, followed by further incubation. Cells were subsequently lysed to isolate total protein, and *mCherry* expression levels were quantified by fluorescence analysis. (B) Normalized *mCherry* protein concentrations. The concentration of **mCh_mRNA_Cap0** was set to 1 Data are presented as mean ± SD (*n* = 4 biologically independent experiments for **mCh_mRNA_Cap0** and **mCh_mRNA_ZipCap‐1a**, technical duplicates for each condition; *n* = 1 for uncapped mRNA). Because absolute fluorescence values varied between independent experiments, protein concentrations were normalized within each biological replicate to the corresponding **mCh_mRNA_Cap0** control. Statistical significance was determined using paired two‐tailed Student’s *t*‐tests comparing each condition to the corresponding **mCh_mRNA_Cap0** control. Exact *P* values are provided in the Supporting Information.

To assess the impact of ZipCap **1a** on mRNA translation, HeLa cells were transfected with *mCherry* encoding mRNA bearing either a Cap0 [16] structure, ZipCap **1a**, or no Cap structure as a negative control (Figure 3A). Four hours post‐transfection, the medium was exchanged and tetrazine **8** was added at varying concentrations. Cells transfected with **mCh_mRNA_Cap0** were harvested 24 h after transfection, whereas cells transfected with **mCh_mRNA_ZipCap‐1a** were harvested 24 h after tetrazine addition to account for the delayed onset of translation. *mCherry* expression was quantified by direct fluorescence measurement using a plate reader assay. Fluorescence signals were normalized to the untreated Cap0 control to allow comparison across conditions (Section S1.2).

As shown in Figure 3B, protein expression from ZipCap **1a** modified mRNA was reduced by ≈34% relative to the Cap0 control (**mCh_mRNA_Cap0**), indicating partial suppression of translation. Upon addition of tetrazine **8**, *mCherry* expression from **mCh_mRNA_ZipCap‐1a** was restored to 89% of the untreated Cap0 level, demonstrating efficient reactivation. Tetrazine was applied over a concentration range of 2–20 µM, with near maximal restoration already observed at 5 µM. At higher concentrations, a slight decrease in protein expression was observed for Cap0 transfected cells, suggesting a concentration‐dependent cellular stress response to tetrazine addition. Notably, no detectable protein expression was observed for uncapped mRNA, confirming the dependence of translation on a functional Cap structure (Figure 3B). We note that previous studies on photocaged FlashCaps [4] as well as recently reported TCO‐based Cap [15] analogues described stronger translational suppression. At present, the molecular basis for this behavior has not yet been fully understood, but it likely reflects the high sensitivity of Cap‐dependent translation to subtle structural and conformational differences within modified Cap architectures. Possible contributing factors include incomplete inhibition of eIF4E binding or partial accessibility of the Cap structure. In addition, since the axial isomer **2a** was used as a racemic mixture and **1a** thus consists of a mixture of diastereomers, it is conceivable that one enantiomer of **2a** efficiently suppresses eIF4E binding, whereas the other still permits interaction. Structural modeling (see Section S1.4) indicates that both R‐ and S‐TCO modified Caps can still be accommodated within the eIF4E binding pocket, although subtle stereochemistry‐dependent steric effects may contribute to incomplete translational suppression. Furthermore, compared to FlashCaps, the absence of the methylene bridge between the carbamate linkage and the (photo‐)cage may represent another contributing factor, potentially decreasing rotational freedom and thereby decreasing blockage of eIF4E binding [4]. Nevertheless, the observed suppression here is fully reversible, as demonstrated by efficient restoration of protein expression upon tetrazine treatment. Together, these findings establish ZipCap **1a** as a chemically responsive Cap analogue that enables external modulation of mRNA translation, while highlighting the need for further studies to elucidate the underlying mechanism and optimize activation conditions.

## 3 Conclusion

In this study, we established a synthetic platform for TCO‐modified 5′ mRNA Cap analogues and evaluated their structure–reactivity relationships. By implementing a controlled carbamate formation strategy, TCO‐modified guanosine derivatives were accessed with reduced reagent excess, addressing limitations associated with synthetically demanding TCO alcohols. Notably, TCO stereochemistry strongly influences click‐to‐release performance, with axial and equatorial isomers showing distinct reactivity and stability, providing design guidelines for bioorthogonally activatable RNA systems. Functional studies demonstrate that ZipCap‐modified mRNA attenuates translation and can be reactivated upon tetrazine treatment, enabling external modulation of protein expression. In contrast to recently described almost complete suppression of translation for related systems, residual translation is consistently observed in our system, highlighting the sensitivity of Cap‐dependent translation to subtle structural variations. Overall, this work complements recent advances in chemically inducible mRNA translation by providing both a synthetic and mechanistic perspective. The ZipCap platform establishes a basis for the rational design of chemically responsive Cap analogues and future strategies for spatiotemporal control of mRNA function.

## Funding

This work was supported by Bundesministerium für Bildung und Forschung (16LW0374) and Universität zu Köln (UoC Forum, project “RACE”).

## Conflicts of Interest

The authors declares no conflicts of interest.

## Supporting information

Supporting information available describing material and methods. The authors have cited additional references within the Supporting Information [24, 25, 26].
