# Supplementary material for: Click to Translate: Synthesis of Trans‐Cyclooctene Modified 5′ mRNA Caps for Bioorthogonal Activation
Source: Chembiochem. 2026 Jun 13;27(11):e70420. doi: 10.1002/cbic.70420 (PMC13264397; doi:10.1002/cbic.70420)
Supplement: Supplementary file 1 — Supporting information available describing material and methods. The authors have cited additional references within the Supporting Information [24, 25, 26]. [file CBIC-27-e70420-s001.pdf]

Supporting Information

Click to translate: synthesis of TCO modified 5' mRNA Caps for bioorthogonal activation

Niclas Zips[a]‡, Ekaterina Kulko[a]‡, Stephanie Kath-Schorr*

[a] University of Cologne, Department of Chemistry and Biochemistry, Institute of Organic Chemistry, 50939 Cologne, Germany

Table of content

1 Methods 1

1.1. General Methods 1

1.2. Biochemical Methods 6

1.3. Uncropped gel images 18

1.4. Structural Models of TCO-modified 5'mRNA Caps 21

2 Syntheses towards TCO-ZipCaps 22

2.1. Synthesis of TBS-protected guanosine **4** 22

2.2. Synthesis of TCO **2a** and **2e** 23

2.3. Synthesis of TBS-protected N^2-TCO_{ax}-guanosine **5a** 24

2.4. Synthesis of Monophosphate **7a** 26

2.5. Synthesis of TBS-protected N^2-TCO_{eq}-guanosine **5e** 27

2.6. Synthesis of Monophosphate **7e** 28

2.7. Synthesis of guanosine 5'-diphosphate imidazolide 32

2.8. Synthesis of ZipCap **1a** 35

2.9. Synthesis of ZipCap **1e** 36

2.10. Synthesis of TCOs **3a** and **3e** 37

2.11. Synthesis of TBS-protected N^2-TCO_{eq}-guanosine **6e** 39

2.12. Synthesis of TBS-protected N^2-TCO_{ax}-guanosine **6a** 41

2.13. Synthesis of Tetrazine **8** 43

3 Prediction of physicochemical properties 44

4 Release and stability assay **1a** 44

4.1. Release Assay of ZipCap **1a** 44

4.2. Stability Assay of ZipCap **1a** 51

5 NMR Spectra 53

6 References 72

1 Methods

1.1. General Methods

Working with Inert Gas

For reactions sensible to water or oxygen, an inert gas/oil pump vacuum system or a glovebox was used. Reaction vessels were evacuated, heated, and flushed with argon three times before usage. As inert gas, argon *BIP*® (99.9997 %) by Air Products was used without further purification.

Solvents and Reactants

Solvents and reactants with a purity of ≥ 98% were used. If not specified otherwise, reactants were used without further purification. Solvents, such as EtOAc, *c*-Hex, MeOH or DCM were distilled before usage. Other dry solvents were bought (*ACRO Seal*, extra dry, stored under argon and over molecular sieves) and used as received.

Unless otherwise stated, all chemicals and solvents employed in synthetic procedures were used directly without further purification as purchased from established suppliers (*Acros Organics, Alfa Aesar, BLD Pharm, Fisher Scientific, Fluka, Merck, Sigma-Aldrich, Carbosynth, Carl Roth, TCI*).

Thin-Layer Chromatography (TLC)

For thin-layer chromatography, *Macherey-Nagel POLYGRAM® Sil G/UV 254* foils were used. They were coated with silica gel (layer thickness 250 µm) and fluorescence indicator. For detection, UV light (λ = 254 nm) was used. Additionally, a potassium permanganate reagent (3 g $KMnO_4$, 20 g K_2CO_3, 5 ml 5% NaOH solution and 300 ml water) for substances sensible to oxidation were used, to make the spots visible. Solvent mixtures are given in volume fraction at the appropriate positions.

Column Chromatography (CC)

For column chromatography, silica gel 60 (0.035-0.07 mm) from *Acros* was used as stationary phase. Solvent mixtures are given in volume fraction at the appropriate positions.

Preparative high-performance liquid chromatography (prep HPLC)

Preparative HPLC purifications were performed on a *puriFLASH 5.250P* HPLC system (*Interchim*). Compounds of low to intermediate molecular weight were purified using an *Interchim US10C18-HQ* column (150 × 21.2 mm, particle size 10 µm) with acetonitrile as solvent B and one of the following aqueous modifiers as solvent A: 0.1 % ammonium acetate (NH4OAc, w/v), 0.1 % formic acid (FA, v/v), or 0.1 % trifluoroacetic acid (TFA, v/v). The specific modifier applied is indicated at the respective position.

Compounds containing phosphates were purified using an *Interchim PFB5C18-XS* column (150 × 21.2 mm, particle size 5 µm) with acetonitrile as solvent B and triethylammonium bicarbonate buffer (TEAB, 0.1 M, pH = 7.5-8) as solvent A.

Additionally, phosphate-containing compounds were purified using a *XBridge BEH Amide OBD Prep Column* (130 Å, 5 µm, 19 mm × 100 mm, *Waters*) with acetonitrile as solvent B and triethylammonium acetate (TEAA, 5 mM) as solvent A.

Reversed-phase flash chromatography (RP-FC)

Reversed-phase flash chromatography (RP-FC) purifications were performed using a *puriFLASH 5.250P* HPLC system (*Interchim*). Different columns were employed depending on the reaction scale. Reactions on a 1-4 g scale were purified using a *FlashPure Select C18* column (particle size: 30 µm; column loading: 40 g). Reactions on a 100-1000 mg scale were purified using an *Interchim PF-30C18HP-F0025* column (particle size: 30 µm; column loading: 25 g). Reactions on a scale of 100 mg or less were purified using an *Interchim PF-15SIHP-F0012* column (particle size: 15 µm; column loading: 12 g). As eluents, acetonitrile (solvent B) and one of the following aqueous modifiers (solvent A) were used: 0.1 % ammonium acetate (NH_4OAc, w/v), 0.1 % formic acid (FA, v/v), or 0.1 M triethylammonium bicarbonate buffer (pH = 7.5-8). The specific modifier applied is indicated at the respective position.

HPLC-MS analysis (LC-MS)

High performance liquid chromatography in combination with mass spectrometry (LC-MS) was performed to monitor reaction progress and to ensure purity and integrity of target compounds. HPLC-MS measurements was performed on an *amaZon SL* mass spectrometer *(Bruker Daltonics)* in combination with an *Elute SP* HPLC system (*Bruker Daltonics*). As a stationary phase a *Zorbax StableBond* (80 Å, C18, 2.1 × 50 mm, 5 µm)

column (*Agilent Technologies*) was employed for low to intermediate molecular weight compounds and a *XBridge Premier* (BEH Amide 2.5 µm VanGuard FIT, 2.1 × 100 mm) column (*Waters*) for phosphates. In general, all compounds were submitted to HPLC-MS using either 0.1 % (w/v) ammonium acetate as solvent A and acetonitrile as solvent B. If not noted otherwise, a gradient from 5 % to 100 % B at a flow rate of 0.8 mL/min for low to intermediate molecular weight compounds and 95 % to 50 % B in 10 min at a flow rate of 0.3 mL/min for phosphates were applied. LC-MS data were interpreted using *Compass DataAnalysis 4.2 or 5.2*.

Nuclear Magnetic Resonance (NMR)

1H NMR spectra were obtained on a Bruker Advance III 500 (500 MHz), a Bruker Avance I 300 (300 MHz), and a Bruker NEO400 evo (400 MHz) spectrometer. Solvents used for NMR analyses included deuterated chloroform ($CDCl_3$), deuterated dimethyl sulfoxide (DMSO-d_6), and deuterium oxide (D_2O). Chemical shifts are reported in parts per million (ppm). In ^{1}H NMR, the residual solvent signals were referenced as follows: $CDCl_3$ at 7.26 ppm, DMSO-d_6 at 2.50 ppm, and the residual HDO signal in D_2O at 4.79 ppm. In ^{13}C NMR, $CDCl_3$ was referenced at 77.16 ppm and DMSO-d_6 at 39.52 ppm. Singlets are denoted "s", doublets "d", triplets "t", quartets "q", and ambiguous multiplets "m". Coupling constants (J) are given in hertz (Hz). ^{13}C NMR spectra were recorded at 126 MHz, 101 MHz, and 75 MHz, while ^{31}P NMR spectra were acquired at 243 MHz. The spectra were interpreted using the software MestReNova v. 12.0.4 by Mestrelab Research S.L. (©2012). Where assignments are ambiguous, signals are separated by "/", overlapping protons or resonances within one signal are separated by ",".

High Resolution Mass Spectrometry (HR-MS)

High resolution mass spectra were recorded on an *LTQ Orbitrap XL* mass spectrometer from *Thermo Fisher Scientific* using electron spray ionisation (ESI) or on an *Exactive GC Orbitrap* mass spectrometer from *Thermo Fisher Scientific* using electron ionisation (EI).

Flow setup for photoisomerization of *cis*-cyclooctenes

The apparatus used for the preparation of *trans*-cyclooctenes is shown in Figure S 1. A photograph is provided in Figure S 2. Before each isomerization, the column, packed with $SiO_2/AgNO_3$ (10:1 w/w), was pre-equilibrated with the respective solvent mixture for at least 2 hours. Depending on the scale of the isomerization reaction, three different column sizes with silica gel loadings of 10 g, 25 g, or 40 g (*Interchim* flash column puriflash dry load empty F0010, F0025, or F0040) were used, corresponding to quartz glass tubes with lengths of 10 cm, 20 cm, or 40 cm, respectively.

Following the injection of the corresponding *cis*-cyclooctene and the photosensitizer (methyl benzoate, 3.0 equiv.), the mixture was passed through the column for a minimum of 1 hour before UV irradiation was initiated. Unless otherwise stated, a flow rate of 20 mL/min was applied. UV light was provided by two 55 W mercury lamps (*Osram*), which were integrated in a custom-made housing. The pump used was a *MERCK HITACHI* L-6250 Intelligent Pump. The setup is based on Mikula *et al.*.[19]

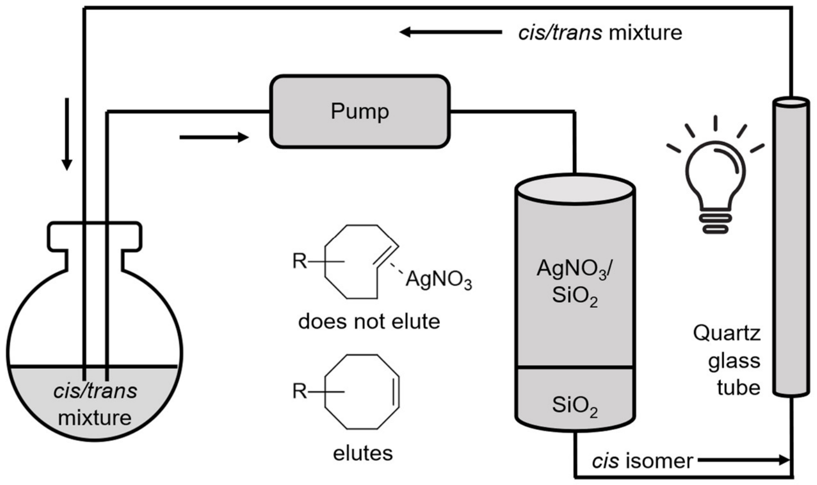

Figure S 1 Photochemical flow setup for the synthesis of TCOs

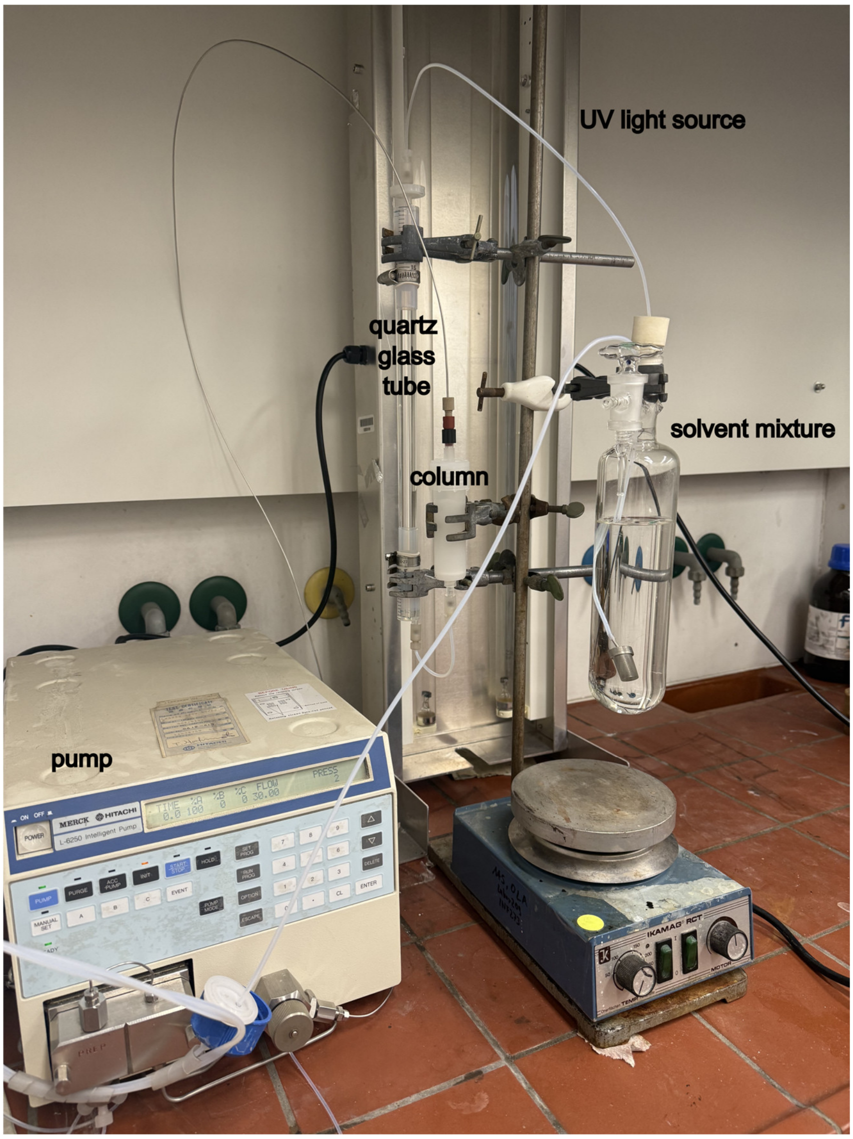

Figure S 2 Depiction of the flow reactor setup for the photoisomerization of cis-cyclooctenes. The reactor comprises a pump, a column filled with $SiO_2/AgNO_3$, a quartz glass tube, a flask for the solvents and reagents, and a UV light source.

Preparation of silver nitrate impregnated silica

Silver nitrate impregnated silica ($SiO_2/AgNO_3$, 10:1 w/w) was prepared by dissolving $AgNO_3$ (20 g) in H_2O (30 mL). The solution was then added to a previously prepared suspension of silica gel 60 (200 g, 0.035-0.070 mm, *Fisher Scientific*) in H_2O (400 mL). The resulting suspension was stirred thoroughly for 2 hours. Afterwards, the solvent was removed *in vacuo*, and the resulting solid was stored protected from light and moisture until further use.[18]

1.2. Biochemical Methods

List of DNA and RNA sequences

***pmCherry-N1* plasmid (4722 bp):**

mCherry protein coding sequence in red

TAGTTATTAATAGTAATCAATTACGGGGTCATTAGTTCATAGCCCATATATGGAG
TTCCGCGTTACATAACTTACGGTAAATGGCCCGCCTGGCTGACCGCCCAACGAC
CCCCGCCCATTGACGTCAATAATGACGTATGTTCCCATAGTAACGCCAATAGGG
ACTTTCCATTGACGTCAATGGGTGGAGTATTTACGGTAAACTGCCCACTTGGCA
GTACATCAAGTGTATCATATGCCAAGTACGCCCCCTATTGACGTCAATGACGGT
AAATGGCCCGCCTGGCATTATGCCCAGTACATGACCTTATGGGACTTTCCTACT
TGGCAGTACATCTACGTATTAGTCATCGCTATTACCATGGTGATGCGGTTTTGGC
AGTACATCAATGGGCGTGGATAGCGGTTTGACTCACGGGGATTTCCAAGTCTCC
ACCCCATTGACGTCAATGGGAGTTTGTTTTGGCACCAAAATCAACGGGACTTTC
CAAAATGTCGTAACAACTCCGCCCCATTGACGCAAATGGGCGGTAGGCGTGTA
CGGTGGGAGGTCTATATAAGCAGAGCTGGTTTAGTGAACCGTCAGATCCGCTA
GCGCTACCGGACTCAGATCTCGAGCTCAAGCTTCGAATTCTGCAGTCGACGGTA
CCGCGGGCCCGGGATCCACCGGTCGCCACCATGGTGAGCAAGGGCGAGGAG
GATAACATGGCCATCATCAAGGAGTTCATGCGCTTCAAGGTGCACATGGAGGG
CTCCGTGAACGGCCACGAGTTCGAGATCGAGGGCGAGGGCGAGGGCCGCCCC
TACGAGGGCACCCAGACCGCCAAGCTGAAGGTGACCAAGGGTGGCCCCCTGC
CCTTCGCCTGGGACATCCTGTCCCCTCAGTTCATGTACGGCTCCAAGGCCTACG
TGAAGCACCCCGCCGACATCCCCGACTACTTGAAGCTGTCCTTCCCCGAGGGC
TTCAAGTGGGAGCGCGTGATGAACTTCGAGGACGGCGGCGTGGTGACCGTGA
CCCAGGACTCCTCCCTGCAGGACGGCGAGTTCATCTACAAGGTGAAGCTGCGC
GGCACCAACTTCCCCTCCGACGGCCCCGTAATGCAGAAGAAGACCATGGGCTG
GGAGGCCTCCTCCGAGCGGATGTACCCCGAGGACGGCGCCCTGAAGGGCGAG
ATCAAGCAGAGGCTGAAGCTGAAGGACGGCGGCCACTACGACGCTGAGGTCAA
GACCACCTACAAGGCCAAGAAGCCCGTGCAGCTGCCCGGCGCCTACAACGTCA
ACATCAAGTTGGACATCACCTCCCACAACGAGGACTACACCATCGTGGAACAGT
ACGAACGCGCCGAGGGCCGCCACTCCACCGGCGGCATGGACGAGCTGTACAA
GTAAAGCGGCCGCGACTCTAGATCATAATCAGCCATACCACATTTGTAGAGGTT
TTACTTGCTTTAAAAAACCTCCCACACCTCCCCCTGAACCTGAAACATAAAATGA

ATGCAATTGTTGTTGTTAACTTGTTTATTGCAGCTTATAATGGTTACAAATAAAGC
AATAGCATCACAAATTTCACAAATAAAGCATTTTTTTCACTGCATTCTAGTTGTGG
TTTGTCCAAACTCATCAATGTATCTTAAGGCGTAAATTGTAAGCGTTAATATTTTG
TTAAAATTCGCGTTAAATTTTTGTTAAATCAGCTCATTTTTTAACCAATAGGCCGA
AATCGGCAAAATCCCTTATAAATCAAAAGAATAGACCGAGATAGGGTTGAGTGTT
GTTCCAGTTTGGAACAAGAGTCCACTATTAAAGAACGTGGACTCCAACGTCAAA
GGGCGAAAAACCGTCTATCAGGGCGATGGCCCACTACGTGAACCATCACCCTA
ATCAAGTTTTTTGGGGTCGAGGTGCCGTAAAGCACTAAATCGGAACCCTAAAGG
GAGCCCCCGATTTAGAGCTTGACGGGGAAAGCCGGCGAACGTGGCGAGAAAG
GAAGGGAAGAAAGCGAAAGGAGCGGGCGCTAGGGCGCTGGCAAGTGTAGCGG
TCACGCTGCGCGTAACCACCACACCCGCCGCGCTTAATGCGCCGCTACAGGGC
GCGTCAGGTGGCACTTTTCGGGGAAATGTGCGCGGAACCCCTATTTGTTTATTT
TTCTAAATACATTCAAATATGTATCCGCTCATGAGACAATAACCCTGATAAATGCT
TCAATAATATTGAAAAAGGAAGAGTCCTGAGGCGGAAAGAACCAGCTGTGGAAT
GTGTGTCAGTTAGGGTGTGGAAAGTCCCCAGGCTCCCCAGCAGGCAGAAGTAT
GCAAAGCATGCATCTCAATTAGTCAGCAACCAGGTGTGGAAAGTCCCCAGGCTC
CCCAGCAGGCAGAAGTATGCAAAGCATGCATCTCAATTAGTCAGCAACCATAGT
CCCGCCCCTAACTCCGCCCATCCCGCCCCTAACTCCGCCCAGTTCCGCCCATT
CTCCGCCCCATGGCTGACTAATTTTTTTTATTTATGCAGAGGCCGAGGCCGCCT
CGGCCTCTGAGCTATTCCAGAAGTAGTGAGGAGGCTTTTTTGGAGGCCTAGGCT
TTTGCAAAGATCGATCAAGAGACAGGATGAGGATCGTTTCGCATGATTGAACAA
GATGGATTGCACGCAGGTTCTCCGGCCGCTTGGGTGGAGAGGCTATTCGGCTA
TGACTGGGCACAACAGACAATCGGCTGCTCTGATGCCGCCGTGTTCCGGCTGT
CAGCGCAGGGGCGCCCGGTTCTTTTTGTCAAGACCGACCTGTCCGGTGCCCTG
AATGAACTGCAAGACGAGGCAGCGCGGCTATCGTGGCTGGCCACGACGGGCG
TTCCTTGCGCAGCTGTGCTCGACGTTGTCACTGAAGCGGGAAGGGACTGGCTG
CTATTGGGCGAAGTGCCGGGGCAGGATCTCCTGTCATCTCACCTTGCTCCTGC
CGAGAAAGTATCCATCATGGCTGATGCAATGCGGCGGCTGCATACGCTTGATCC
GGCTACCTGCCCATTCGACCACCAAGCGAAACATCGCATCGAGCGAGCACGTA
CTCGGATGGAAGCCGGTCTTGTCGATCAGGATGATCTGGACGAAGAGCATCAG
GGGCTCGCGCCAGCCGAACTGTTCGCCAGGCTCAAGGCGAGCATGCCCGACG
GCGAGGATCTCGTCGTGACCCATGGCGATGCCTGCTTGCCGAATATCATGGTG
GAAAATGGCCGCTTTTCTGGATTCATCGACTGTGGCCGGCTGGGTGTGGCGGA
CCGCTATCAGGACATAGCGTTGGCTACCCGTGATATTGCTGAAGAGCTTGGCG

GCGAATGGGCTGACCGCTTCCTCGTGCTTTACGGTATCGCCGCTCCCGATTCG
CAGCGCATCGCCTTCTATCGCCTTCTTGACGAGTTCTTCTGAGCGGGACTCTGG
GGTTCGAAATGACCGACCAAGCGACGCCCAACCTGCCATCACGAGATTTCGATT
CCACCGCCGCCTTCTATGAAAGGTTGGGCTTCGGAATCGTTTTCCGGGACGCC
GGCTGGATGATCCTCCAGCGCGGGGATCTCATGCTGGAGTTCTTCGCCCACCC
TAGGGGGAGGCTAACTGAAACACGGAAGGAGACAATACCGGAAGGAACCCGC
GCTATGACGGCAATAAAAAGACAGAATAAAACGCACGGTGTTGGGTCGTTTGTT
CATAAACGCGGGGTTCGGTCCCAGGGCTGGCACTCTGTCGATACCCCACCGAG
ACCCCATTGGGGCCAATACGCCCGCGTTTCTTCCTTTTCCCCACCCCACCCCCC
AAGTTCGGGTGAAGGCCCAGGGCTCGCAGCCAACGTCGGGGCGGCAGGCCCT
GCCATAGCCTCAGGTTACTCATATATACTTTAGATTGATTTAAAACTTCATTTTTA
ATTTAAAAGGATCTAGGTGAAGATCCTTTTTGATAATCTCATGACCAAAATCCCTT
AACGTGAGTTTTCGTTCCACTGAGCGTCAGACCCCGTAGAAAAGATCAAAGGAT
CTTCTTGAGATCCTTTTTTTCTGCGCGTAATCTGCTGCTTGCAAACAAAAAAACC
ACCGCTACCAGCGGTGGTTTGTTTGCCGGATCAAGAGCTACCAACTCTTTTTCC
GAAGGTAACTGGCTTCAGCAGAGCGCAGATACCAAATACTGTCCTTCTAGTGTA
GCCGTAGTTAGGCCACCACTTCAAGAACTCTGTAGCACCGCCTACATACCTCGC
TCTGCTAATCCTGTTACCAGTGGCTGCTGCCAGTGGCGATAAGTCGTGTCTTAC
CGGGTTGGACTCAAGACGATAGTTACCGGATAAGGCGCAGCGGTCGGGCTGAA
CGGGGGGTTCGTGCACACAGCCCAGCTTGGAGCGAACGACCTACACCGAACTG
AGATACCTACAGCGTGAGCTATGAGAAAGCGCCACGCTTCCCGAAGGGAGAAA
GGCGGACAGGTATCCGGTAAGCGGCAGGGTCGGAACAGGAGAGCGCACGAGG
GAGCTTCCAGGGGGAAACGCCTGGTATCTTTATAGTCCTGTCGGGTTTCGCCAC
CTCTGACTTGAGCGTCGATTTTTGTGATGCTCGTCAGGGGGGCGGAGCCTATG
GAAAAACGCCAGCAACGCGGCCTTTTTACGGTTCCTGGCCTTTTGCTGGCCTTT
TGCTCACATGTTCTTTCCTGCGTTATCCCCTGATTCTGTGGATAACCGTATTACC
GCCATGCAT

Primer sequences

mCherry forward primer (39 nt):

T7 promoter sequence underlined, after promotor sequence two additional nucleotides GG (dotted line) were added for enhanced T7 RNA polymerase transcription start)

5' TAATACGACTCACTATAGGGCTCAAGCTTCGAATTCTGC 3'

mCherry reverse primer (24 nt):

5' ACAAATGTGGTATGGCTGATTATG 3'

mCherry DNA gene sequence (834 bp):

TAATACGACTCACTATAGGGCTCAAGCTTCGAATTCTGCAGTCGACGGTACCGC
GGGCCCGGGATCCACCGGTCGCCACCATGGTGAGCAAGGGCGAGGAGGATAA
CATGGCCATCATCAAGGAGTTCATGCGCTTCAAGGTGCACATGGAGGGCTCCG
TGAACGGCCACGAGTTCGAGATCGAGGGCGAGGGCGAGGGCCGCCCCTACGA
GGGCACCCAGACCGCCAAGCTGAAGGTGACCAAGGGTGGCCCCCTGCCCTTC
GCCTGGGACATCCTGTCCCCTCAGTTCATGTACGGCTCCAAGGCCTACGTGAA
GCACCCCGCCGACATCCCCGACTACTTGAAGCTGTCCTTCCCCGAGGGCTTCA
AGTGGGAGCGCGTGATGAACTTCGAGGACGGCGGCGTGGTGACCGTGACCCA
GGACTCCTCCCTGCAGGACGGCGAGTTCATCTACAAGGTGAAGCTGCGCGGCA
CCAACTTCCCCTCCGACGGCCCCGTAATGCAGAAGAAGACCATGGGCTGGGAG
GCCTCCTCCGAGCGGATGTACCCCGAGGACGGCGCCCTGAAGGGCGAGATCA
AGCAGAGGCTGAAGCTGAAGGACGGCGGCCACTACGACGCTGAGGTCAAGAC
CACCTACAAGGCCAAGAAGCCCGTGCAGCTGCCCGGCGCCTACAACGTCAACA
TCAAGTTGGACATCACCTCCCACAACGAGGACTACACCATCGTGGAACAGTACG
AACGCGCCGAGGGCCGCCACTCCACCGGCGGCATGGACGAGCTGTACAAGTA
AAGCGGCCGCGACTCTAGATCATAATCAGCCATACCACATTTGT

mCherry mRNA sequence (mCh_mRNA) (817 nt):

GGGCUCAAGCUUCGAAUUCUGCAGUCGACGGUACCGCGGGCCCGGGAUCCA
CCGGUCGCCACCAUGGUGAGCAAGGGCGAGGAGGAUAACAUGGCCAUCAUCA
AGGAGUUCAUGCGCUUCAAGGUGCACAUGGAGGGCUCCGUGAACGGCCACG
AGUUCGAGAUCGAGGGCGAGGGCGAGGGCCGCCCCUACGAGGGCACCCAGA
CCGCCAAGCUGAAGGUGACCAAGGGUGGCCCCCUGCCCUUCGCCUGGGACA
UCCUGUCCCCUCAGUUCAUGUACGGCUCCAAGGCCUACGUGAAGCACCCCGC
CGACAUCCCCGACUACUUGAAGCUGUCCUUCCCCGAGGGCUUCAAGUGGGAG
CGCGUGAUGAACUUCGAGGACGGCGGCGUGGUGACCGUGACCCAGGACUCC
UCCCUGCAGGACGGCGAGUUCAUCUACAAGGUGAAGCUGCGCGGCACCAACU
UCCCCUCCGACGGCCCCGUAAUGCAGAAGAAGACCAUGGGCUGGGAGGCCU
CCUCCGAGCGGAUGUACCCCGAGGACGGCGCCCUGAAGGGCGAGAUCAAGC

AGAGGCUGAAGCUGAAGGACGGCGGCCACUACGACGCUGAGGUCAAGACCAC
CUACAAGGCCAAGAAGCCCGUGCAGCUGCCCGGCGCCUACAACGUCAACAUC
AAGUUGGACAUCACCUCCCACAACGAGGACUACACCAUCGUGGAACAGUACG
AACGCGCCGAGGGCCGCCACUCCACCGGCGGCAUGGACGAGCUGUACAAGU
AAAGCGGCCGCGACUCUAGAUCAUAAUCAGCCAUACCACAUUUGU

DNA template preparation

DNA template was prepared by PCR amplification from the *mCherry* coding region of plasmid *pmCherry-N1*, using one pair of primers. The forward primer holding the T7 promotor sequence as a 5'-overhang was utilized to introduce the sequence into the amplicon during PCR experiments. PCR amplification reaction for the **mCh_DNA** construct was carried out using 50 ng plasmid per 100 µL PCR reaction mixture containing 1 µM forward and reverse primers, 375 µM each natural dNTP (*Jena Bioscience*), 1X *OneTaq® Standard Reaction Buffer* (*New England Biolabs*) and 2.5 Units *OneTaq® DNA polymerase* (*New England Biolabs*). PCR was performed with an initial denaturing step at 94°C for 2 min, followed by 30 cycles of denaturing at 94°C for 30 s, annealing at 94°C for 40 s, elongation at 68 °C for 1 min and a final elongation step at 68 °C for 3 min. PCR products were analyzed by agarose gel electrophoresis and purified using the *NucleoSpin®* Gel and PCR Clean-Up Kit *(Macherey-Nagel)* according to the manufacturer’s protocol.

mRNA *in vitro* transcription

For *in vitro* transcription (IVT) of mRNA coding for *mCherry* protein (**mCh_mRNA**), the *HiScribe® T7 Quick High Yield RNA Synthesis Kit* (*New England Biolabs*) was used. According to the manufacturer’s protocol, differently capped mRNAs were transcribed from the double stranded DNA template (**mCh_DNA)**. Therefore 1 µg of purified DNA template was used per reaction (20 µL). Following DNase template digestion by addition of 4 U DNase I to the reaction mixture and incubation at 37°C for 15 min, the transcribed capped mRNA (**mCh_mRNA_Cap0, mCh_mRNA_ZipCap-1a** and **mCh_mRNA_WT** containing anti-reverse cap analog, ARCA.[5] as well as uncapped mRNA were purified via LiCl precipitation and subsequently polyadenylated using *E. coli* Poly(A) Polymerase (*New England Biolabs*) according to the manufacturer’s protocol.

After purification, the Cap0-, ARCA- and ZipCap **1a**-capped mRNAs were subjected to enzymatic digestion to remove uncapped mRNA. According to the manufacturer's protocols, the 5′-polyphosphatase (1 µL, 20 units; *Biosearch Technologies*) and Terminator 5′-phosphate-dependent exonuclease (1 µL, 1 U; *Biosearch Technologies*) reactions are recommended to be performed in total reaction volumes of 20 µL containing up to 5 µg and 10 µg mRNA, respectively. Since larger mRNA quantities were processed (Table S 1), both reactions were scaled up proportionally while maintaining the same reagent concentrations and reaction conditions. Following the manufacturer's protocol, the mRNA was purified prior to exonuclease digestion. Final purification was performed by LiCl precipitation.

Capping efficiencies of different cap analogues were evaluated by enzymatic digestion of uncapped mRNA followed by quantification of the remaining capped transcripts. The results indicate efficient incorporation of ZipCap **1a** during *in vitro* transcription.

	**Amount of mRNA obtained from the IVT reaction**	**Amount of mRNA taken before enzymatic digestion of uncapped mRNA**	**Amount of mRNA obtained after enzymatic digestion of uncapped mRNA**	**Calculated capping efficiency**
Cap0	40262 ng	15000 ng	11087 ng	73,91%
ZipCap **1a**	29078 ng	15000 ng	13026 ng (mean value from 2 different enzymatic reactions)	86,84%
ARCA	37500 ng	8400 ng	7243 ng	86,23%

Table S 1 Yield of the IVT reaction and determination of mRNA capping efficiency for different cap analogues.

Cell culture and mRNA transfection

Adherent HeLa cells (RRID:CVCL_0030) were obtained from ATCC (Manassas, VA, USA) and cultured in *Dulbecco's Modified Eagle Medium (1x DMEM) GlutaMAX™* (*gibco™, Thermo Fisher Scientific*) supplemented with 10 % Fetal Bovine Serum (FBS) (*Sigma*), 1 % Minimum Essential Medium (MEM) non-essential amino acids solution (100X, *gibco™, Thermo Fisher Scientific*) and 1 % sodium pyruvate (100 mM, *gibco™, Thermo Fisher Scientific*) in cell culture flasks T75 (*LABSOLUTE®*) at 37 °C and 5 % CO_2. Cell confluence was kept between 80-90 %, therefore cells were split at regular

intervals of two to three days. Therefore, cells were washed with 8 mL Dulbecco's Phosphate Buffered Saline (1X DPBS) (*gibco™, Thermo Fisher Scientific*), trypsinized with 2 mL Trypsin-EDTA (0.05%) (*gibco™, Thermo Fisher Scientific*) and incubated at 37 °C and 5 % CO_2 for 5 to 10 min, before partly transferred into a new cell culture flask with fresh DMEM medium.

For **mCh_mRNA** transfection experiments cells were seeded in a *TC Plate 24 well, Standard (Sarstedt)* to a density of 50.000 cells per well in 500 µL *DMEM* medium the day before transfection. HeLa cells were transfected using *Lipofectamine® MessengerMAX™ (Invitrogen)* transfection reagent. According to the manufacturer's protocol, for each well of cells to be transfected 0.75 µL *Lipofectamine® MessengerMAX™* was diluted in *Opti-MEM® Reduced-Serum Medium* (*gibco™, Thermo Fisher Scientific*) up to 25 µL, vortexed thoroughly and incubated at room temperature (RT) for 10 min. Likewise 500 ng **mCh_mRNA** (consisted for all different **mCh_mRNA** sequences) was diluted in *Opti-MEM®* up to 25 µL. Diluted mRNA and diluted *MessengerMAX™* were mixed in an 1:1 ratio, vortexed thoroughly and incubated at RT for 5 min. The mRNA-lipid complex was then added drop-wise to the cell medium and incubated at 37 °C and 5 % CO_2. The cells, that were transfected with **mCh_mRNA_Cap0,** were harvested after 24 h post-transfection. However, those cells that were transfected with **mCh_mRNA_ZipCap-1a** and uncapped mRNA, were incubated for 24 h post-tetrazine addition and then harvested. 4 h after transfection start the cell medium was exchanged for fresh DMEM medium and the Tz **8** was added onto cells drop-wise with different concentrations.

***mCherry* protein quantification**

mCherry protein quantification was performed using a *mCherry Quantification Kit* (*BioVision*) in combination with *RIPA Lysis and Extraction Buffer (Thermo Fisher Scientific™)*. After transfection of **mCh_mRNA** and subsequent incubation to allow for mRNA translation and *mCherry* reporter protein expression, the cell culture medium was removed and replaced with 130 µL of assay buffer (*RIPA Lysis and Extraction Buffer* supplemented with 1 mM phenylmethylsulfonyl fluoride (PMSF), *Thermo Fisher Scientific™*). After incubation on ice for 10-15 min, the samples were centrifuged at 10000 x g, 4°C for 5 min. The supernatants were directly taken for accomplishment of

the quantification assay. Each 100 µL of supernatant was transferred into the 96-well plate. Using a 10 ng/µL *mCherry* working solution a *mCherry* standard series of 0, 20, 40, 60, 80, 100 ng/well was prepared as duplicate in a 96-well plate and all volumes adjusted to 100 µL with assay buffer. *mCherry* fluorescence was measured at Ex/Em = 587/610 nm at 25 °C using a plate reader (*BioTek Synergy 2*). All measured fluorescence values were offset corrected and the *mCherry* standard curve was plotted. The fluorescence readings of samples were applied to the *mCherry* standard curve to calculate the amount of *mCherry* protein in the sample wells. Mean values of *mCherry* protein amounts were calculated for technical duplicates of each transfection condition (**mCh_mRNA_Cap0, mCh_mRNA_ZipCap-1a** and uncapped mRNA). Also, *mCherry* protein amounts for corresponding transfection conditions from biologically independent experiments were averaged (n=4 for **mCh_mRNA_Cap0** and **mCh_mRNA_ZipCap-1a;** n=1 for uncapped mCherry mRNA).

Raw data of fluorescence (RFU) and *mCherry* protein standard curves for quantification

Exp. #1

***mCherry* protein standard [ng/well]**	**0**	**20**	**40**	**60**	**80**	**100**
**Standard curve #1**	45	94	142	190	234	281
**Standard curve #2**	43	95	138	193	233	271
**mean value**	44,00	94,5	140	191,5	233,5	276

**Slope line of best fit**	2,3264
**Coefficient of determination R^2**	0,9987

	**Well #1**	**Well #2**	**mean**
**mCh_mRNA_Cap0**	460,0	409,0	434,5
**mCh_mRNA_Cap0 +Tz8_10µM**	383,0	368,0	375,5
**mCh_mRNA_ZipCap-1a-**	286,0	298,0	292,0
**mCh_mRNA_ZipCap-1a+Tz8_2µM**	335,0	329,0	332,0
**mCh_mRNA_ZipCap-1a+Tz8_5µM**	372,0	409,0	390,5
**mCh_mRNA_ZipCap-1a+Tz8_10µM**	401,0	384,0	392,5
**mCh_mRNA_ZipCap-1a+Tz8_20µM**	396	401	398,5

**H_2O**	49	49	49,0

Exp. #2

mCherry protein standard [ng/well]	0	20	40	60	80	100
**Standard curve #1**	43	111	175	214	273	334
**Standard curve #2**	38	111	162	209	256	340
**mean value**	40,50	111	168,5	211,5	264,5	337

**Slope line of best fit**	2,8371
**Coefficient of determination R^2**	0,9943

	Well #1	Well #2	mean
**mCh_mRNA_Cap0**	327,0	368,0	347,5
**mCh_mRNA_Cap0 +Tz8_10µM**	326,0	287,0	306,5
**mCh_mRNA_ZipCap-1a-**	242,0	244,0	243,0
**mCh_mRNA_ZipCap-1a+Tz8_2µM**	295,0	307,0	301,0
**mCh_mRNA_ZipCap-1a+Tz8_5µM**	328,0	319,0	323,5
**mCh_mRNA_ZipCap-1a+Tz8_10µM**	306,0	315,0	310,5
**mCh_mRNA_ZipCap-1a+Tz8_20µM**	273	285	279,0
**H_2O**	64	68	66,0

Exp. #3

mCherry protein standard [ng/well]	0	20	40	60	80	100
**Standard curve #1**	191	453	658	851	1051	1205
**Standard curve #2**	192	436	646	837	1048	1259
**mean value**	191,50	444,5	652	844	1049,5	1232

**Slope line of best fit**	10,2990
**Coefficient of determination R^2**	0,9974

	Well #1	Well #2	mean
**mCh_mRNA_Cap0**		1804,0	1804,0
**mCh_mRNA_Cap0 +Tz8_10µM**	1612,0	1671,0	1641,5

**mCh_mRNA_ZipCap-1a-**	1292,0	1220,0	1256,0
**mCh_mRNA_ZipCap-1a+Tz8_2µM**	1323,0	1340,0	1331,5
**mCh_mRNA_ZipCap-1a+Tz8_5µM**	1360,0	1445,0	1402,5
**mCh_mRNA_ZipCap-1a+Tz8_10µM**	1317,0	1460,0	1388,5
**mCh_mRNA_ZipCap-1a+Tz8_20µM**	1442	1538	1490,0
**H_2O**	218	204	211,0

Exp. #4

***mCherry* protein standard [ng/well]**	**0**	**20**	**40**	**60**	**80**	**100**
**Standard curve #1**	36,5	91	130	167	201	241
**Standard curve #2**	37,5	92	139	182,5	229	275
**mean value**	37,00	91,5	134,5	174,75	215	258

**Slope line of best fit**	2,1654
**Coefficient of determination R^2**	0,9972

	**Well #1**	**Well #2**	**mean**
**mCh_mRNA_Cap0**	314,0	352,0	333,0
**mCh_mRNA_Cap0 +Tz8_10µM**	278,0	287,0	282,5
**mCh_mRNA_ZipCap-1a-**	247	243	245,0
**mCh_mRNA_ZipCap-1a+Tz8_2µM**	264,0	277,0	270,5
**mCh_mRNA_ZipCap-1a+Tz8_5µM**	321,0	337,0	329,0
**mCh_mRNA_ZipCap-1a+Tz8_10µM**	276,0	267,0	271,5
**mCh_mRNA_ZipCap-1a+Tz8_20µM**	245	267	256,0
**mCh_mRNA_uncapped**	40	51	45,5
**H_2O**	37	38	37,5

Table S 2 RFU and *mCherry* protein standard curves for quantification.

Statistical analysis

**Comparison**	**Statistical test**	**n**	***P* value**	**Significance**
Cap0 vs Cap0 + Tz **8** (10 µM)	Paired two-tailed Student's *t*-test	4	0.0026	**
Cap0 vs ZipCap-1a			0.0039	**
Cap0 vs ZipCap-1a + Tz **8** (2 µM)			0.0372	*

Cap0 vs ZipCap-1a + Tz **8** (5 µM)			0.0456	*
Cap0 vs ZipCap-1a + Tz **8** (10 µM)			0.2685	ns
Cap0 vs ZipCap-1a + Tz **8** (20 µM)			0.6556	ns

Table S 3 Statistical analysis of *mCherry* fluorescence measurements (calculated protein concentrations).

Data are presented as mean ± standard deviation (SD) of biological replicates (n = 4), unless otherwise stated. Fluorescence values were converted to protein concentrations using the corresponding standard curve and normalized to the corresponding **mCh_mRNA_Cap0** control within each independent experiment. Statistical analyses were performed using Microsoft Excel (*Microsoft Corporation*). Comparisons between conditions and the matched **mCh_mRNA_Cap0** control were conducted using paired two-tailed Student's *t*-tests. Statistical significance was defined as $p < 0.05$ ($\alpha = 0.05$). The uncapped mRNA sample was measured once (n = 1) and was therefore used only as a qualitative negative control and excluded from statistical analysis.

In vitro click reaction

8 pmol each of **mCh_mRNA_CP** as a positive control that was synthesized previously in our research group following the procedure according to Bornewasser *et al.*.[4],[5] **mCh_mRNA_WT** as a negative control, **mCh_mRNA_ZipCap-1a** and **mCh_mRNA_ZipCap-1e** were incubated with 10 µM tetrazine-conjugated fluorophore solution (BDP® FL tetrazine, **Tz-FL**, *Lumiprobe Corporation,* Cas: 2042193-77-9) for 1 h in the dark. Then 100 µL of each reaction solution was transferred into the 96-well plate and the fluorescence was measured at Ex/Em = 485/528 nm at 25 °C using a plate reader (*BioTek Synergy 2*).

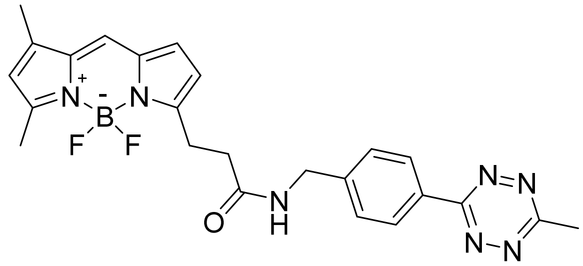

Tz-FL

Analytical agarose gel electrophoresis

For analytical agarose gel electrophoresis, 2 % (w/v) (for **mCh_DNA** and **mCh_mRNA** sequences) solution of agarose high resolution (*Carl Roth*).

For **mCh_DNA** the agarose solution was prepared by dissolving agarose in 0.5 % Tris-borate-EDTA buffer (0.5X TBE, Tris Base, *Fisher Scientific*, Boric acid, *Sigma-Aldrich*, EDTA, *Fisher Scientific*) supplemented with 0.0001% SYBR® Safe DNA stain (*Life Technologies*). Samples were prepared using 6X DNA loading buffer (*Thermo Fisher Scientific*) and subsequently loaded on the gel. 0.5X TBE was employed as running buffer. Gel electrophoresis was performed at 130 V const. for 20 min. Gene Ruler 100 bp DNA Ladder (*Thermo Fisher Scientific*) was used as dsDNA standard for **mCh_DNA** sequence.

For **mCh_mRNA** the agarose solution was prepared by dissolving agarose in 1 % Tris-acetate-EDTA buffer (0.5X TBE, Tris Base, *Fisher Scientific*, Acetic acid, *Fisher Scientific*, EDTA, *Fisher Scientific*) supplemented with 0.0001% SYBR® Safe DNA stain (*Life Technologies*). Samples (500 ng mRNA each) were prepared using 2X RNA Loading Dye (*Thermo Fisher Scientific*) and subsequently loaded on the gel. 1X TBE was employed as running buffer. Gel electrophoresis was performed at 130 V const. for 25 min. RiboRuler Low Range RNA Ladder (*Thermo Fisher Scientific*) was used as ssRNA standard for **mCh_mRNA** sequences.

Analytical agarose gels were visualized by UV illumination using a CHEMOSTAR PC ECL & Fluorescence Imager (*INTAS Science Imaging Instruments*).

PAGE gel electrophoresis and in-gel click reaction

A 4% denaturing PAGE gel solution was prepared by combining 0.8 mL of 25% bisacrylamide/acrylamide (Bis/AA) in 8.3 M urea, 2.92 mL of 8.3 M urea in H_2O, 500 µL of 8.3 M urea in 10X TBE, 725 µL H_2O, 50 µL of 10% ammonium persulfate (APS), and 5 µL tetramethylethylenediamine (TEMED). The solution was mixed thoroughly, carefully poured into a slab gel sandwich to the top, and allowed to polymerize at room temperature for 40-60 min. Samples were prepared by mixing 2,5-3,5 µg of each mRNA (5 µL) with 5 µL of formamide/ethylenediaminetetraacetic acid (FA/EDTA) loading buffer containing urea. Before sample loading, the gel was prerun in 1X TBE buffer at 130-150 V for 20 min. Samples were then run under the same conditions for 60 min. The gel was subsequently incubated in a 3 µM **Tz-FL** for 1 h. The resulting

mRNA bands were first analyzed by fluorescence scanning using the CHEMOSTAR PC ECL & Fluorescence Imager (*INTAS Science Imaging*) with an excitation wavelength of λ_ex = 475 nm and an emission wavelength of λ_em = 520 nm, followed by post-staining with SYBR® Safe DNA stain (*Life Technologies*) and visualization under UV illumination.

Nucleic acid concentration determination

Concentrations of DNA and RNA samples were determined by absorption at 260 nm (A_{260}) using a DeNovix DS-11 Series Spectrophotometer/Fluorometer (*DeNovix Inc.).* Concentrations were obtained from the A_{260} value and software assisted calculation.

1.3. Uncropped gel images

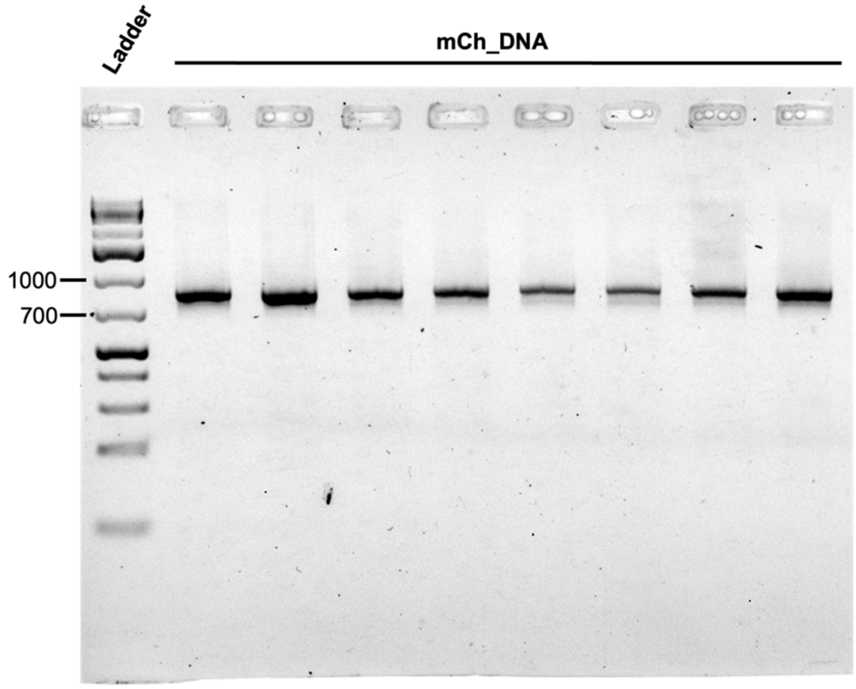

Figure S 3 2% Agarose gel of the PCR products for the **mCh_DNA** template preparation. GeneRuler 1 kb DNA Plus (*Thermofisher*) was used as marker (Ladder).

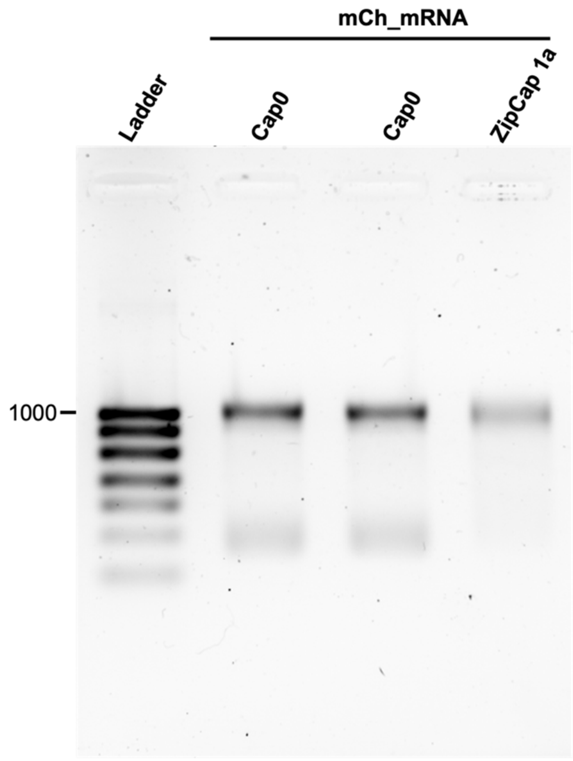

Figure S 4. 2% Agarose gel of the differently capped **mCh_mRNA**. RiboRuler™ Low Range RNA Ladder (*Thermofisher*) was used as marker (Ladder).

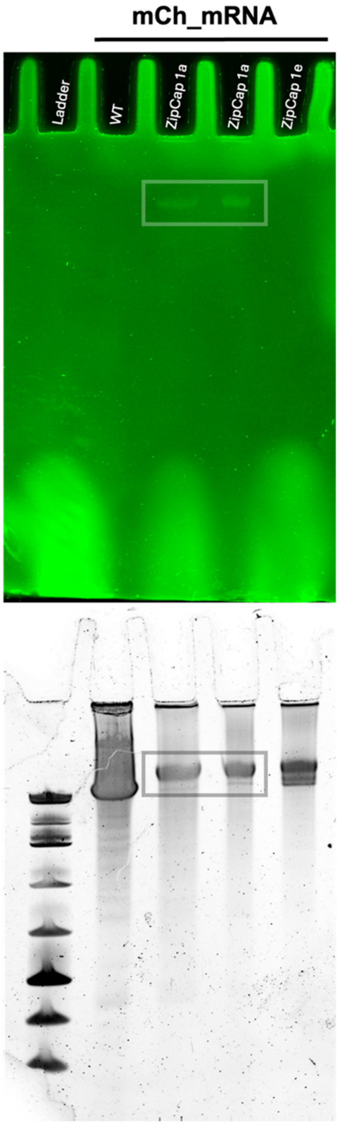

Figure S 3. 4% denaturing PAGE followed by in-gel click reaction with **Tz-FL**. From left to right: **mCh_mRNA_WT** as negative control; **mCh_mRNA_ZipCap-1a** two samples from two different syntheses and **mRNA_ZipCap-1e**. GeneRuler 1 kb DNA Plus (*Thermofisher*) was used as marker (Ladder). The upper panel shows fluorescence detection, while the lower panel displays SYBR® Safe staining after UV illumination. Segments and explanations are shown in Figure 2.

1.4 Structural Models of TCO-modified 5’mRNA Caps

A B

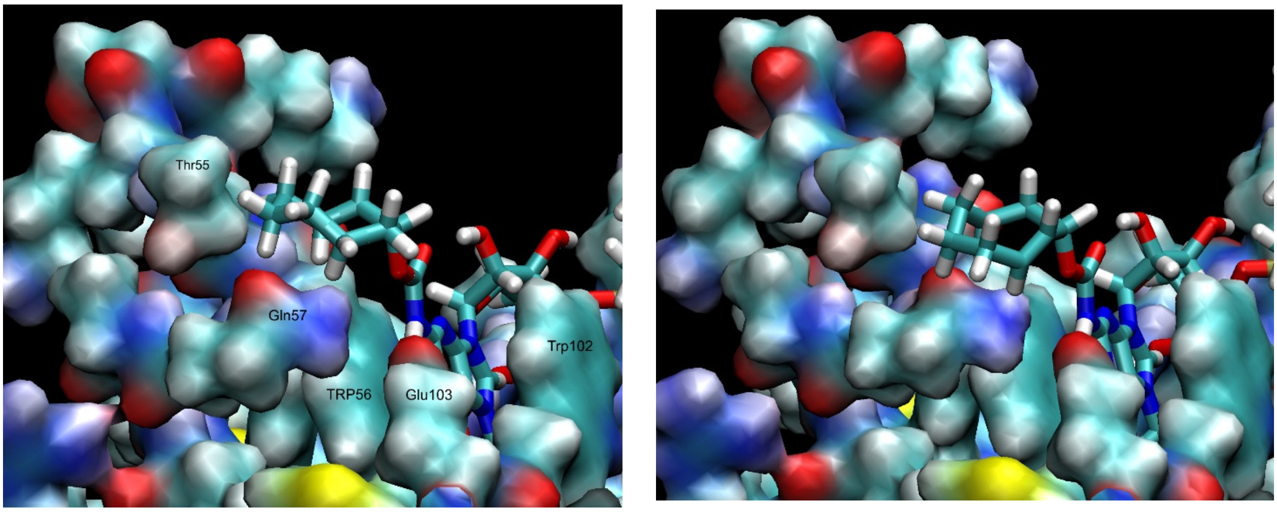

Structure models of the TCO modified 5’ mRNA Caps complexed by the eukaryotic initiation factor 4E (eIF4E). Structures with the TCO enantiomer R (A, left) and S (B, right) are shown.

The basis of the model structures is the crystal structure 1IPB (Tomoo et al., *Biochem. J.*, 362, 539-544, 2002) of the Cap-model 7-Me-GPPA in eIF4E. R- and S-TCO (**2a**) were connected by modelling to N2 of G via the carbamate linker. The structures are enclosed in a cubic box filled by 27770 water molecules and 80 sodium- and 79 chloride-ions followed by an energy minimization Gromacs using the Charmm force field (Charmm36, CgGenFF 5.0, Wacha et al. J. Chem Inf. Model. 2023. 63, 4246-4252).

The 7-Me-G of the 5’ mRNA Cap is seen in the center, embedded between the aromatic pi-faces of Trp102 and Trp56. The Cap is further fixed by hydrogen bonding of the N2-H- and N1-H hydrogens of the 7-Me-G group to the carboxylate of Glu103 of eIF4E (residue numbering in 1IPB). The R- and S-TCO groups are located in a molecular cleft (or valley) occupying some free space. The TCO groups come in (weak) contact to the residues Gln 57 and Thr55 (this is more pronounced with R-TCO). In summary, the presented energy minimized model structures do not exclude binding of R- and S-TCO modified 5’ mRNA caps to eIF4E.

2 Syntheses towards TCO-ZipCaps

2.1. Synthesis of TBS-protected guanosine **4**

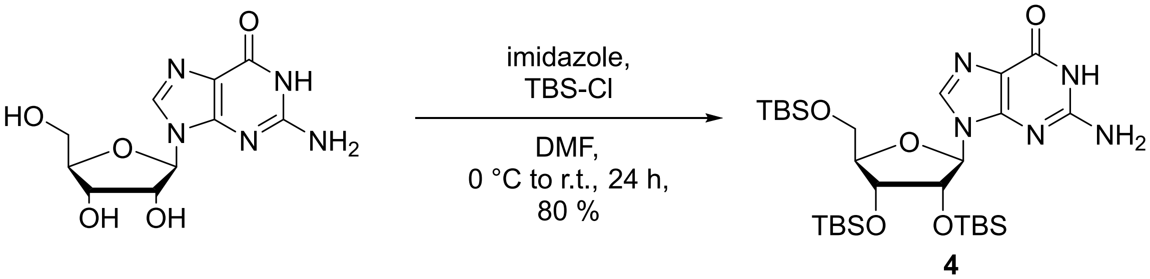

Guanosine (10 g, 35 mmol, 1.0 equiv.) and imidazole (24 g, 353 mmol, 10.0 equiv.) were dissolved in DMF (100 mL). The solution was cooled to 0 °C and TBS-Cl (32 g, 212 mmol, 6.0 equiv.) was added in small portions. After complete addition, the ice bath was removed and the reaction mixture was stirred at r.t. for 18 h. Subsequently, TBS-Cl (11 g, 71 mmol, 2.0 equiv.) was added and stirring was continued for an additional 6 h.

The volatiles were removed *in vacuo*, and CH_2Cl_2 (200 mL) and H_2O (200 mL) were added. The layers were separated, and the aqueous layer was extracted with CH_2Cl_2 (3 × 200 mL). The combined organic layers were washed with H_2O (4 × 200 mL) and dried over $MgSO_4$. The solvent was removed *in vacuo*, and the crude product was filtered over a pad of silica (SiO_2, *c*-Hex:EtOAc = 4:1 to 1:4). TBS-protected guanosine **4** (18 g, 28 mmol, 80%) was obtained as a colorless solid.

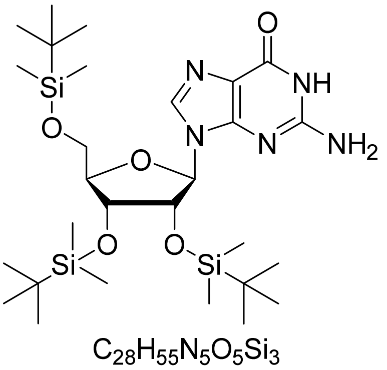

$C_{28}H_{55}N_5O_5Si_3$
626.03

TLC: R_f = 0.1 (SiO_2, *c*-Hex:EtOAc = 1:2).

^{1}H NMR (500 MHz, DMSO-*d*$_6$) δ [ppm] = 10.61 (br. s, 1H), 7.89 (s, 1H), 6.45 (br. s, 2H), 5.74 (d, *J* = 7.1 Hz, 1H), 4.59 (dd, *J* = 7.1 Hz, 4.6 Hz, 1H), 4.19–4.16 (m, 1H), 3.95 (t, *J* = 3.9 Hz, 1H), 3.85 (dd, *J* = 11.2 Hz, 5.5 Hz, 1H), 3.71 (dd, *J* = 11.2 Hz, 3.7 Hz, 1H), 0.91–0.90 (m, 18H), 0.73 (s, 9H), 0.12 (s, 3H), 0.10–0.09 (m, 9H), −0.08 (s, 3H), −0.28 (s, 3H).

^{13}C NMR (126 MHz, DMSO-d_6) δ [ppm] = 156.7, 153.8, 151.6, 135.1, 116.5, 85.7, 85.2, 75.2, 72.8, 63.0, 25.8, 25.70, 25.5, 18.0, 17.8, 17.5, −4.7, −4.8, −4.8, −5.5, −5.5, −5.5.
HR MS (ESI): calculated for $[M+H]^+$ = 626.3583758; found: m/z = 626.35790.

2.2. Synthesis of TCO **2a** and **2e**

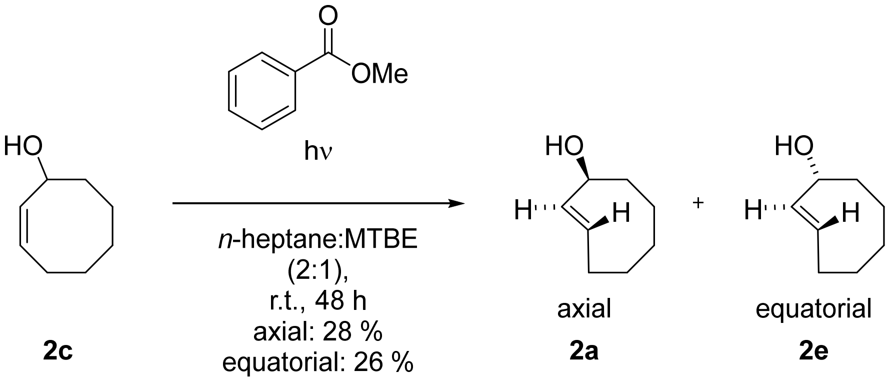

Alcohol **2c** (2.5 g, 19.8 mmol, 1.0 equiv.) and methyl benzoate (8.1 g, 7.4 mL, 59.4 mmol, 3.0 equiv.) were dissolved in *n*-heptane:MTBE (2:1, 350 mL). The solution was irradiated for 48 h while continuously passed through a column filled with silica/$AgNO_3$ (10:1, 25 g). The column was kept in the dark during irradiation. Subsequently, the column was rinsed with *n*-pentane: Et_2O (1:1, 700 mL) and CH_2Cl_2 (700 mL).

The silica/$AgNO_3$/*trans*-cyclooctene mixture was transferred into an Erlenmeyer flask and stirred with CH_2Cl_2 (250 mL) and aqueous ammonia (conc., 50 mL) at room temperature for 15 min. The solids were filtered off and the layers were separated. The aqueous layer was extracted with CH_2Cl_2 (3 × 150 mL), and the combined organic layers were dried over $MgSO_4$. The solvent was removed *in vacuo*, and the crude product was purified by column chromatography (*n*-pentane:Et_2O = 10:1 to 3:2).

2a (axial isomer, 700 mg, 5.5 mmol, 28%) and **2e** (equatorial isomer, 661 mg, 5.2 mmol, 26%) were obtained as colorless oils.

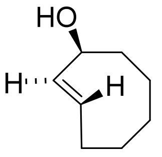

(±)
axial
$C_8H_{14}O$
126.20
2a

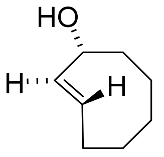

(±)
equatorial
$C_8H_{14}O$
126.20
2e

Axial isomer:

TLC: R_f = 0.37 (SiO_2, *c*-Hex:EtOAc = 4:1).

¹H NMR (400 MHz, $CDCl_3$) δ [ppm] = 5.96 (ddd, *J* = 16.5 Hz, 11.1 Hz, 3.7 Hz, 1H), 5.58 (dd, *J* = 16.5 Hz, 2.2 Hz, 1H), 4.61 (br. s, 1H), 2.52–2.46 (m, 1H), 2.08–1.92 (m, 3H), 1.90–1.81 (m, 1H), 1.72–1.45 (m, 4H), 1.16–1.07 (m, 1H), 0.81–0.71 (m, 1H).

¹³C NMR (101 MHz, $CDCl_3$) δ [ppm] = 135.3, 130.7, 71.4, 43.4, 36.2, 36.0, 29.4, 23.3.

Equatorial isomer:

TLC: R_f = 0.30 (SiO_2, *c*-Hex:EtOAc = 4:1).

¹H NMR (400 MHz, $CDCl_3$) δ [ppm] = 5.70–5.62 (m, 1H), 5.57–5.51 (m, 1H), 4.29–4.23 (m, 1H), 2.42–2.37 (m, 1H), 2.18–2.12 (m, 1H), 2.00–1.73 (m, 5H), 1.54–1.34 (m, 2H), 0.93–0.85 (m, 1H), 0.80–0.71 (m, 1H).

¹³C NMR (101 MHz, $CDCl_3$) δ [ppm] = 135.7, 132.2, 77.2, 44.5, 36.0, 35.6, 29.2, 27.8.

HR MS (EI): calculated for $[M]^+$ = 126.10392; found: m/z = 126.1039. The analytical data are in accordance with literature.[8]

2.3. Synthesis of TBS-protected N^2-TCO_{ax}-guanosine **5a**

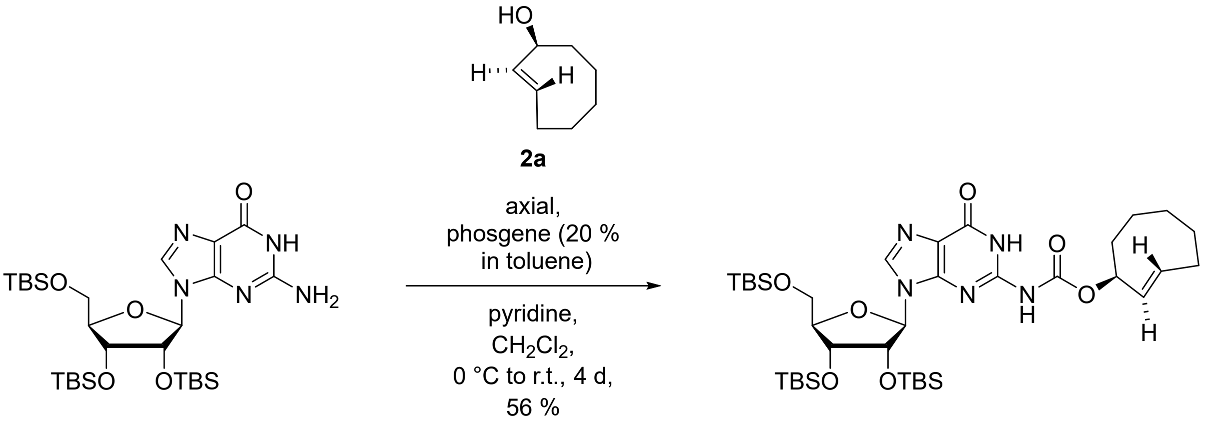

4 **5a**

In an inert atmosphere, protected guanosine **4** (4.5 g, 7.0 mmol, 1.0 equiv.) and pyridine (2.8 mL, 2.8 g, 35 mmol, 5.0 equiv.) were dissolved in CH_2Cl_2 (40 mL). The solution was cooled to 0 °C, and phosgene (20% in toluene, 1.4 g, 14.0 mmol, 2.0 equiv., corresponding to a total amount of 7.4 mL, 6.9 g) was added dropwise. The reaction mixture was stirred at 0 °C for 30 min.

Subsequently, a solution of axial TCO **2a** (1.3 g, 10.6 mmol, 1.5 equiv.) in CH_2Cl_2 (20 mL) was added dropwise, and the mixture was stirred for 4 days while gradually

warming to room temperature. The volatiles were removed *in vacuo*, and the crude product was purified by column chromatography (SiO_2, *c*-Hex:EtOAc = 1:1).

TCO_{ax}-functionalized guanosine **5a** (3.1 g, 4.0 mmol, 56%) was obtained as a colorless solid.

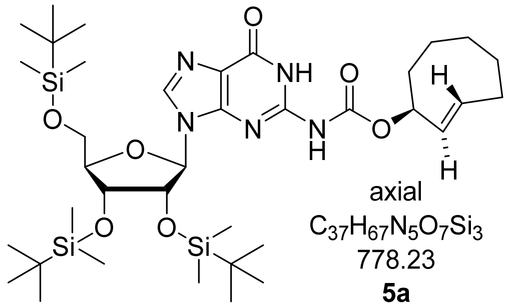

axial
$C_{37}H_{67}N_5O_7Si_3$
778.23
5a

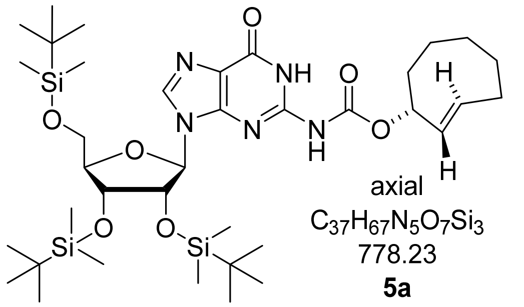

axial
$C_{37}H_{67}N_5O_7Si_3$
778.23
5a

TLC: R_f = 0.40 (SiO_2, *c*-Hex:EtOAc = 1:1).

^{1}H NMR (400 MHz, $CDCl_3$) δ [ppm] = 11.24 (s, 1H), 8.00 (s, 1H), 7.46–7.45 (m, 1H), 5.89–5.80 (m, 2H), 5.56–5.50 (m, 2H), 4.41–4.39 (m, 1H), 4.26–4.24 (m, 1H), 4.09–4.08 (m, 1H), 3.90 (dd, *J* = 11.4, 3.5 Hz, 1H), 3.77 (dd, *J* = 11.4, 2.2 Hz, 1H), 2.51–2.49 (m, 1H), 2.16–1.67 (m, 8H), 1.58–1.43 (m, 1H), 1.12–1.01 (m, 1H), 0.95–0.94 (m, 18H), 0.80–0.79 (m, 9H), 0.13–0.11 (m, 12H), −0.04 (s, 3H), −0.24 (s, 3H).

^{13}C NMR (101 MHz, $CDCl_3$) δ [ppm] = 155.6, 152.6, 148.6, 146.5, 136.9, 133.1, 133.1, 129.6, 129.6, 121.2, 121.1, 87.3, 87.3, 86.2, 86.2, 77.0, 77.0, 76.8, 76.8, 72.6, 72.6, 63.0, 40.6, 40.6, 36.1, 36.1, 36.1, 36.1, 29.0, 26.2, 26.0, 25.8, 25.8, 24.2, 24.2, 18.7, 18.7, 18.2, 18.0, 18.0, −4.3, −4.3, −4.4, −4.4, −4.5, −5.0, −5.0, −5.2, −5.3).

Multiple overlaps in ^{1}H and ^{13}C NMR are observed due to diastereomer formation.

HR MS (ESI): calculated for $[M+Na]^+$ = 800.4241; found: m/z = 800.4240.

2.4. Synthesis of Monophosphate **7a**

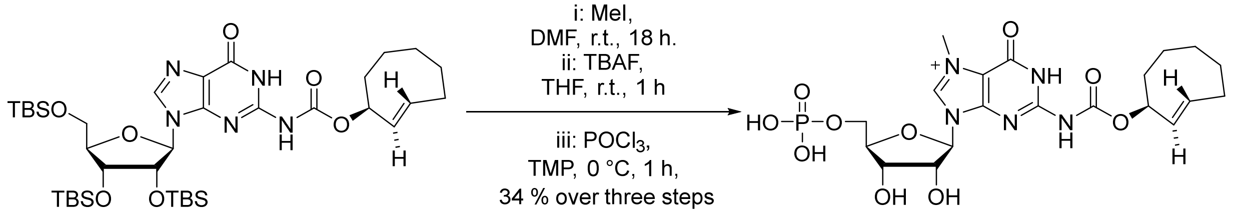

5a **7a**

TCO-functionalized guanosine **5a** (1.1 g, 1.4 mmol, 1.0 equiv.) was dissolved in DMF (40 mL). MeI (5.3 mL, 12 g, 85 mmol, 60 equiv.) was added, and the reaction mixture was stirred at r.t. for 18 h. The volatiles were removed *in vacuo*.

Subsequently, THF (21.5 mL) and TBAF (1 M in THF, 3.5 mL, 3.5 mmol, 2.5 equiv.) were added, and the mixture was stirred at r.t. for 1 h. The volatiles were removed *in vacuo*, and the crude product was purified by RP flash chromatography (C18, 40 g, H_2O + 0.1% FA:ACN = 10–50% over 20 column volumes). The fractions containing the product were combined (LC–MS: calculated for $[M]^+$ = 450.20; found: m/z = 450.20), and the solvent was removed *in vacuo* to dryness to afford the product (470 mg, 1.1 mmol, 75%).

Subsequently, TMP (2.0 mL) was added, and the suspension was cooled to 0 °C. $POCl_3$ (131 µL, 219 mg, 1.4 mmol, 1.3 equiv.) was added, and the mixture was stirred at 0 °C for 1 h. Cooled TEAB (1 M in H_2O, 2 mL) was added slowly, and the mixture was stirred for 5 min. The crude mixture was directly subjected to RP flash chromatography (C18, 25 g, H_2O + 0.1 M TEAB:ACN = 0–50% over 20 column volumes).

Monophosphate **7a** (253 mg, 0.48 mmol, 43%, 34% over three steps) was obtained as a colorless solid, likely as the triethylammonium salt.

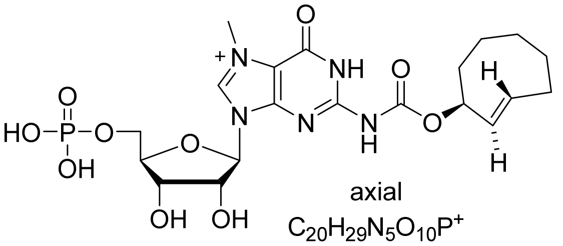

axial
$C_{20}H_{29}N_5O_{10}P^+$
530.45
7a

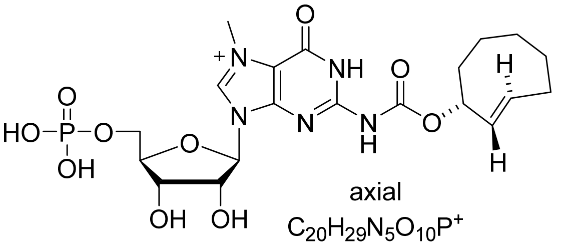

axial
$C_{20}H_{29}N_5O_{10}P^+$
530.45
7a

^{1}H NMR (600 MHz, D_2O) δ [ppm] = 6.15–6.13 (m, 1H), 5.88–5.82 (m, 1H), 5.63–5.60 (m, 1H), 5.35–5.33 (m, 1H), 4.65–4.63 (m, 1H), 4.45–4.43 (m, 1H), 4.39–4.27 (m, 1H), 4.20–4.18 (m, 1H), 4.15 (m, 3H), 2.41–2.39 (m, 2H), 2.08–2.06 (m, 1H), 2.03–1.96 (m, 1H), 1.95–1.90 (m, 1H), 1.85–1.79 (m, 2H), 1.66–1.61 (m, 1H), 1.49–1.43 (m, 1H), 1.03–0.99 (m, 1H), 0.82–0.76 (m, 1H).

^{13}C NMR (151 MHz, D_2O) δ [ppm] = 156.6, 154.0, 152.3, 152.2, 147.9, 137.5, 133.0, 129.9, 111.6, 90.1, 84.6, 84.6, 76.9, 75.2, 75.2, 69.5, 69.5, 63.1, 63.1, 39.6, 36.0, 35.4, 35.4, 28.2, 23.5.

Multiple overlaps in ^{1}H and ^{13}C NMR are observed due to diastereomer formation.

^{31}P NMR (243 MHz, D_2O, ^{1}H-decoupled) δ [ppm] = 0.98.

HR MS (ESI): calculated for $[M]^+$ = 530.1647; found: m/z = 530.1650.

2.5. Synthesis of TBS-protected N²-TCO$_{eq}$-guanosine **5e**

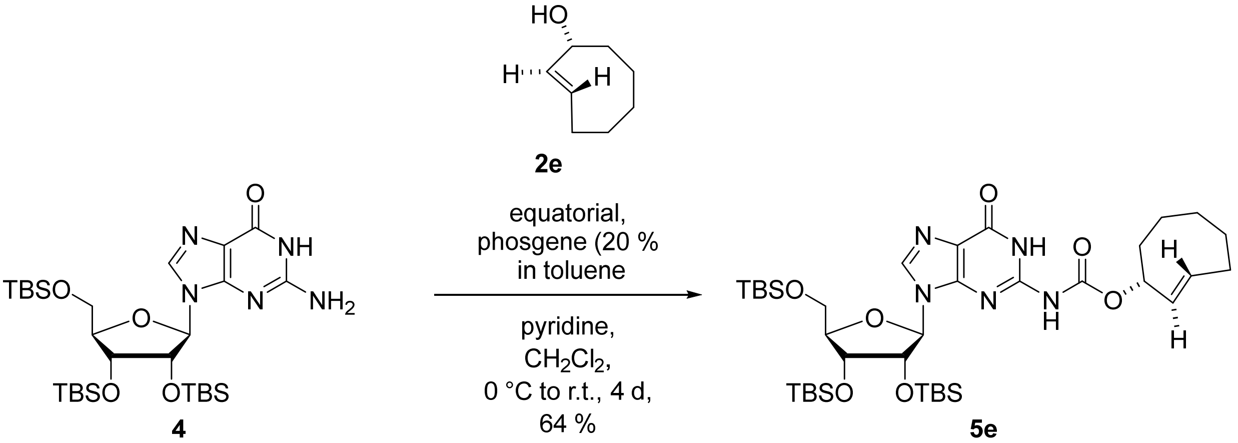

Under an inert atmosphere, protected guanosine TBS-G **4a** (4.2 g, 6.6 mmol, 1.0 equiv.) and pyridine (2.6 mL, 2.6 g, 33 mmol, 5.0 equiv.) were dissolved in CH_2Cl_2 (50 mL). The solution was cooled to 0 °C, and phosgene (20% in toluene, 1.3 g, 13.2 mmol, 2.0 equiv., corresponding to a total amount of 7.0 mL, 6.6 g) was added dropwise. The reaction mixture was stirred at 0 °C for 30 min.

Subsequently, a solution of **2e** (1.26 g, 10.0 mmol, 1.5 equiv.) in CH_2Cl_2 (10 mL) was added dropwise, and the mixture was stirred for 4 days while gradually warming to room temperature. The volatiles were removed *in vacuo*, and the crude product was purified by column chromatography (SiO_2, *c*-Hex:EtOAc = 1:1).

TCO-functionalized guanosine **5e** (3.3 g, 4.2 mmol, 64%) was obtained as a colorless solid.

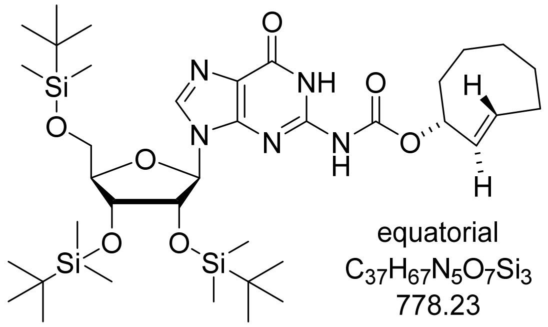

equatorial
$C_{37}H_{67}N_5O_7Si_3$
778.23
5e

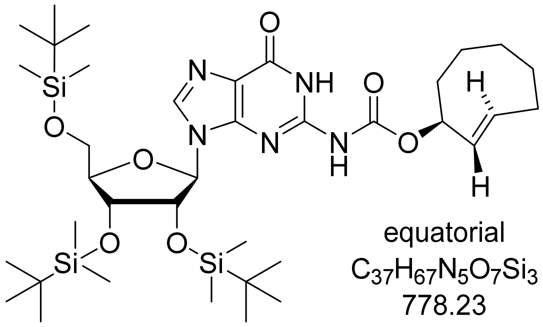

equatorial
$C_{37}H_{67}N_5O_7Si_3$
778.23
5e

TLC: R_f = 0.40 (SiO_2, *c*-Hex:EtOAc = 1:1).

¹H NMR (400 MHz, $CDCl_3$) δ [ppm] = 11.25 (br. s, 1H), 8.03–8.01 (m, 1H), 7.42 (br. s, 1H), 5.87–5.79 (m, 2H), 5.66 (ddd, *J* = 16.3, 9.6, 3.2 Hz, 1H), 5.27–5.20 (m, 1H), 4.36–4.32 (m, 1H), 4.25–4.23 (m, 1H), 4.08–4.06 (m, 1H), 3.91 (dd, *J* = 11.4, 3.4 Hz, 1H), 3.76 (dd, *J* = 11.4, 2.3 Hz, 1H), 2.48–2.42 (m, 1H), 2.30–2.22 (m, 1H), 2.03–1.93 (m, 2H), 1.83–1.80 (m, 2H), 1.64–1.54 (m, 1H), 1.50–1.38 (m, 1H), 0.95–0.88 (m, 19H), 0.80 (s, 9H), 0.13–0.12 (m, 6H), 0.10–0.08 (m, 6H), −0.04 (s, 3H), −0.21 (s, 3H).

¹³C NMR (101 MHz, $CDCl_3$) δ [ppm] = 155.6, 153.0, 148.6, 148.6, 146.6, 136.7, 134.9, 134.8, 130.1, 121.1, 121.0, 87.4, 85.8, 81.3, 77.1, 77.0, 72.2, 72.2, 62.8, 40.8, 40.7, 35.7, 35.6, 28.6, 27.5, 26.2, 26.0, 25.8, 18.7, 18.2, 17.6, −4.3, −4.3, −4.5, −4.6, −4.6, −4.9, −4.9, −5.3, −5.3.

Multiple overlaps in ¹H and ¹³C NMR are observed due to diastereomer formation.

HR MS (ESI): calculated for $[M+Na]^+$ = 800.4241; found: m/z = 800.4240.

2.6. Synthesis of Monophosphate **7e**

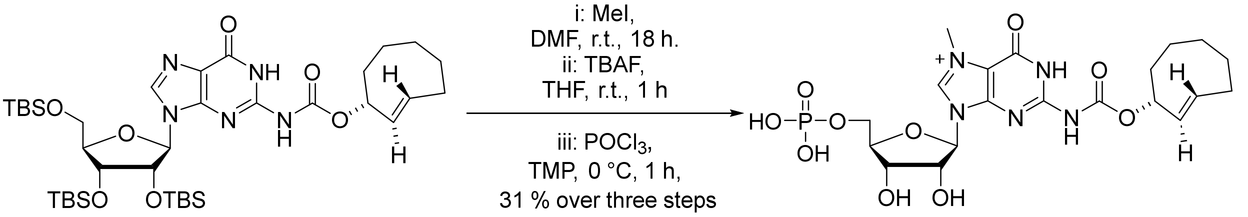

5e **7e**

TCO$_{eq}$-functionalized guanosine **5e** (1.1 g, 1.4 mmol, 1.0 equiv.) was dissolved in DMF (40 mL). MeI (5.3 mL, 12 g, 85 mmol, 60 equiv.) was added, and the reaction mixture was stirred at r.t. for 18 h. The volatiles were removed *in vacuo*.

Subsequently, THF (21.5 mL) and TBAF (1 M in THF, 3.5 mL, 3.5 mmol, 2.5 equiv.) were added, and the mixture was stirred at r.t. for 1 h. The volatiles were removed *in vacuo*, and the crude product was purified by flash chromatography (C18, 40 g, H_2O +

0.1% FA:ACN = 10–50% over 20 column volumes). The fractions containing the product were combined (LC–MS: calculated for $[M]^+$ = 450.20; found: m/z = 450.20), and the solvent was removed *in vacuo* to dryness to afford the product (524 mg, 1.2 mmol, 82%).

A portion of this material (400 mg, 0.89 mmol, 1.0 equiv.) was used for the subsequent step. The material was dissolved in TMP (1.5 mL), and the suspension was cooled to 0 °C. $POCl_3$ (106 µL, 178 mg, 1.2 mmol, 1.3 equiv.) was added, and the mixture was stirred at 0 °C for 1 h. Cooled TEAB (1 M in H_2O, 2 mL) was added slowly, and the mixture was stirred for 5 min. The crude mixture was directly subjected to RP flash chromatography (C18, 25 g, H_2O + 0.1 M TEAB:ACN = 0–50% over 20 column volumes).

Monophosphate **7e** (180 mg, 0.34 mmol, 38%, 31% over three steps) was obtained as a colorless solid, likely as the triethylammonium salt.

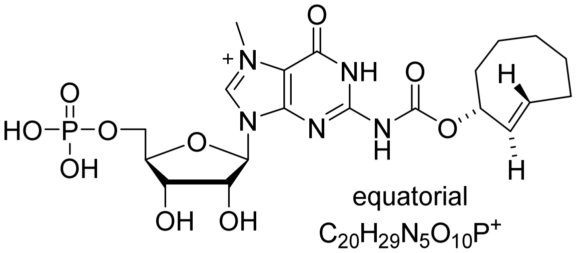

equatorial
$C_{20}H_{29}N_5O_{10}P^+$
530.45
7e

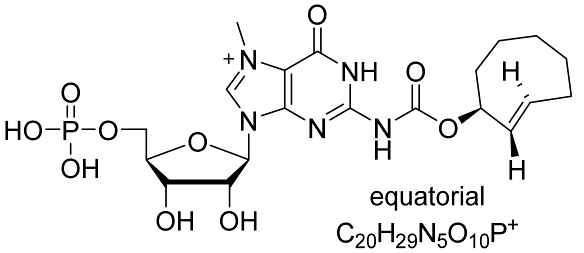

equatorial
$C_{20}H_{29}N_5O_{10}P^+$
530.45
7e

Note: During NMR measurements in D_2O, ^1H NMR spectra was recorded at different time points (time = 0 and 3 days later). Analysis indicated a gradual release of the TCO moiety from the guanosine scaffold.

LC–MS (ESI): calculated for $[M]^+$ = 530.16; found: m/z = 530.17.

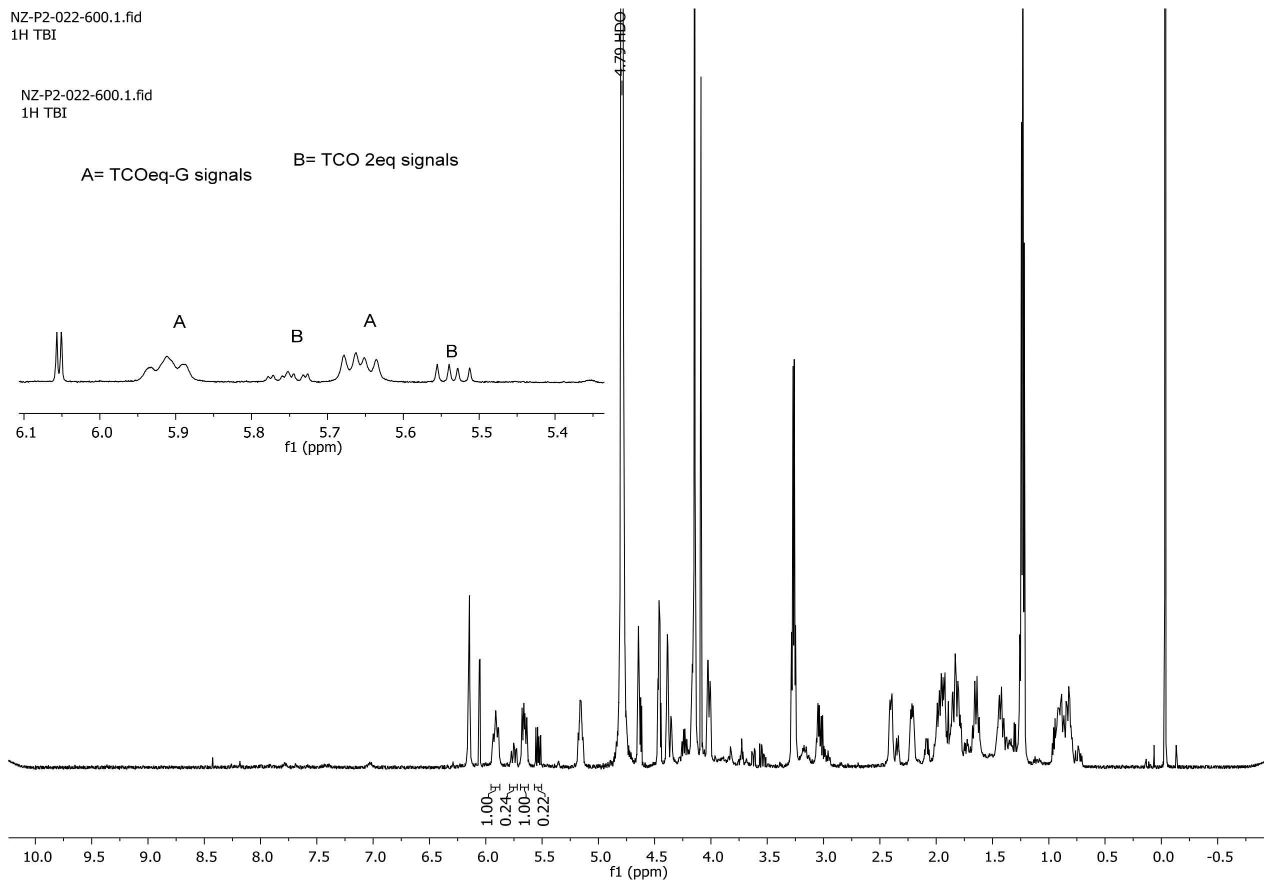

Figure S 4 ^{1}H-NMR (600 MHz, D_2O) spectra of TCOeq-MP **7e**. The zoomed area shows the relevant peaks to support the claim of instability in aqueous media.

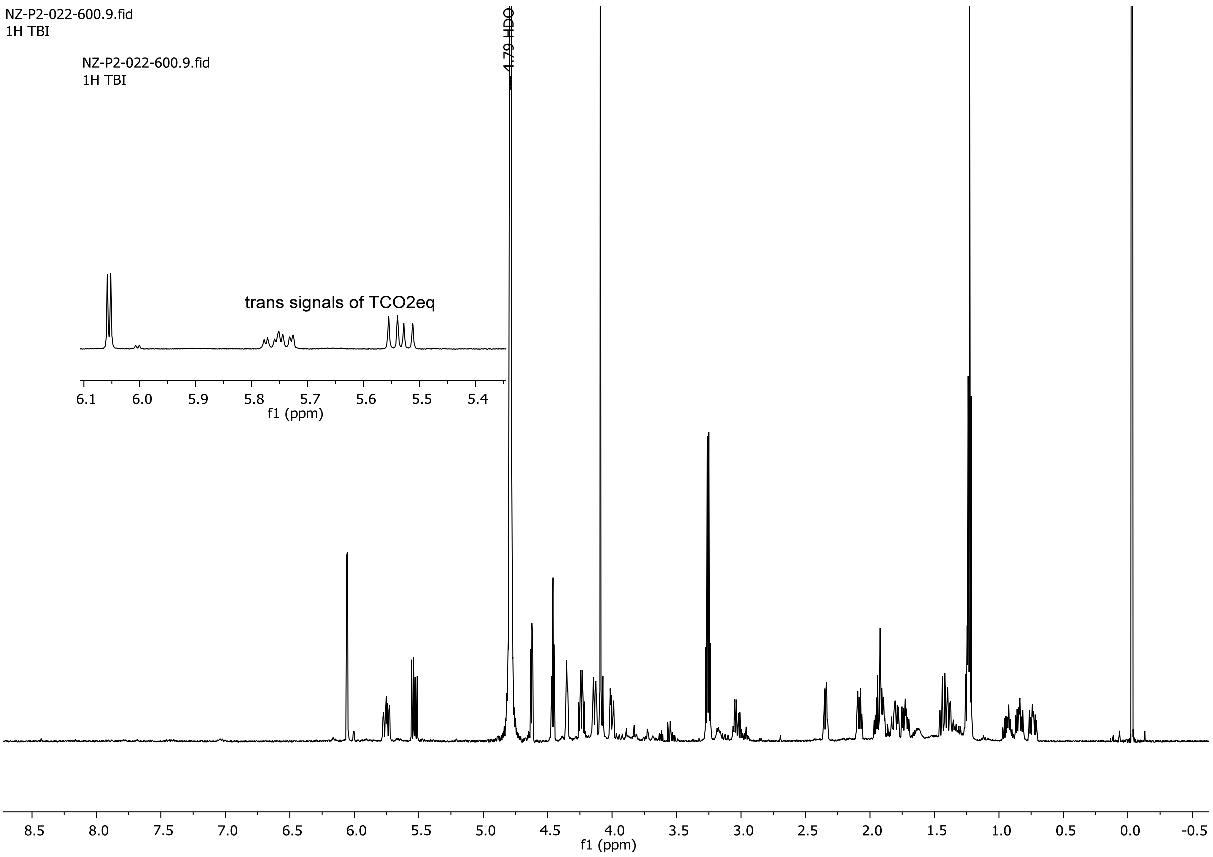

Figure S 5 ^{1}H-NMR (600 MHz, D_2O) of TCOeq-MP **7e** three days later. The signals belonging to the TCOeq attached to the guanosine scaffold disappeared, indicating instability.

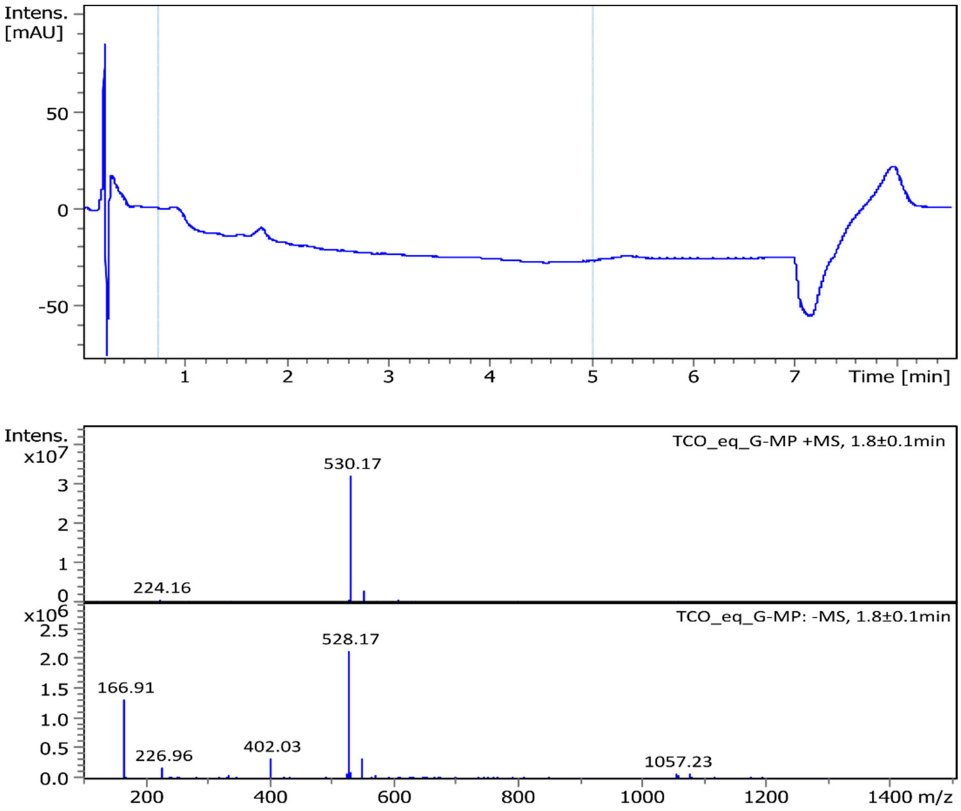

Figure S 6 LC-MS analysis of **7e**.

2.7. Synthesis of guanosine 5'-diphosphate imidazolide

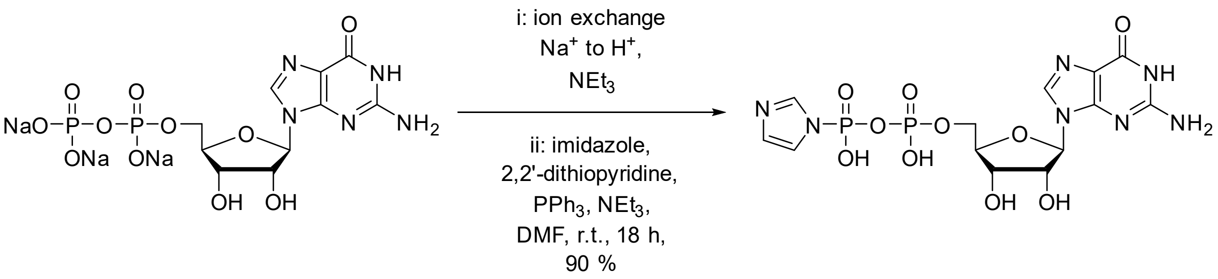

The reaction was performed following a procedure by Jemielity *et al.*.[24]

Guanosine diphosphate sodium salt (2.00 g, 4.00 mmol, 1.0 equiv.) was dissolved in H_2O (10.0 mL) and converted to the corresponding free acid using an ion-exchange resin (Dowex 50 WX8, 200–400 mesh, H^+ form). NEt_3 (868 µL, 626 mg, 12.4 mmol, 3.10 equiv.) was added, and the solvent was removed *in vacuo* to dryness.

The resulting ammonium salt was dissolved in DMF (30.0 mL), and imidazole (1.36 g, 20.0 mmol, 5.0 equiv.), 2,2′-dithiopyridine (1.76 g, 8.00 mmol, 2.0 equiv.), PPh_3 (2.10 g, 8.00 mmol, 2.0 equiv.), and NEt_3 (554 µL, 404 mg, 4.00 mmol, 1.0 equiv.) were added. The reaction mixture was stirred at r.t. for 18 h.

The mixture was then added to a solution of $NaClO_4$ (2.20 g, 18.0 mmol, 4.5 equiv.) in dry acetone (100 mL, 0 °C). The mixture was stirred for 15 min at r.t. and subsequently stored at 4 °C for 2 h. The precipitate was collected by filtration and washed with dry acetone (2 × 30 mL). Guanosine 5′-diphosphate imidazolide(1.80 g, 3.60 mmol, 90 %) was obtained as a yellow solid.

LC–MS analysis indicated a diphosphate-to-monophosphate ratio of approximately 5:1 (area integration: 83:17).

HR MS (ESI): calculated for $[M-H]^-$ = 492.0439359; found: m/z = 492.04409.

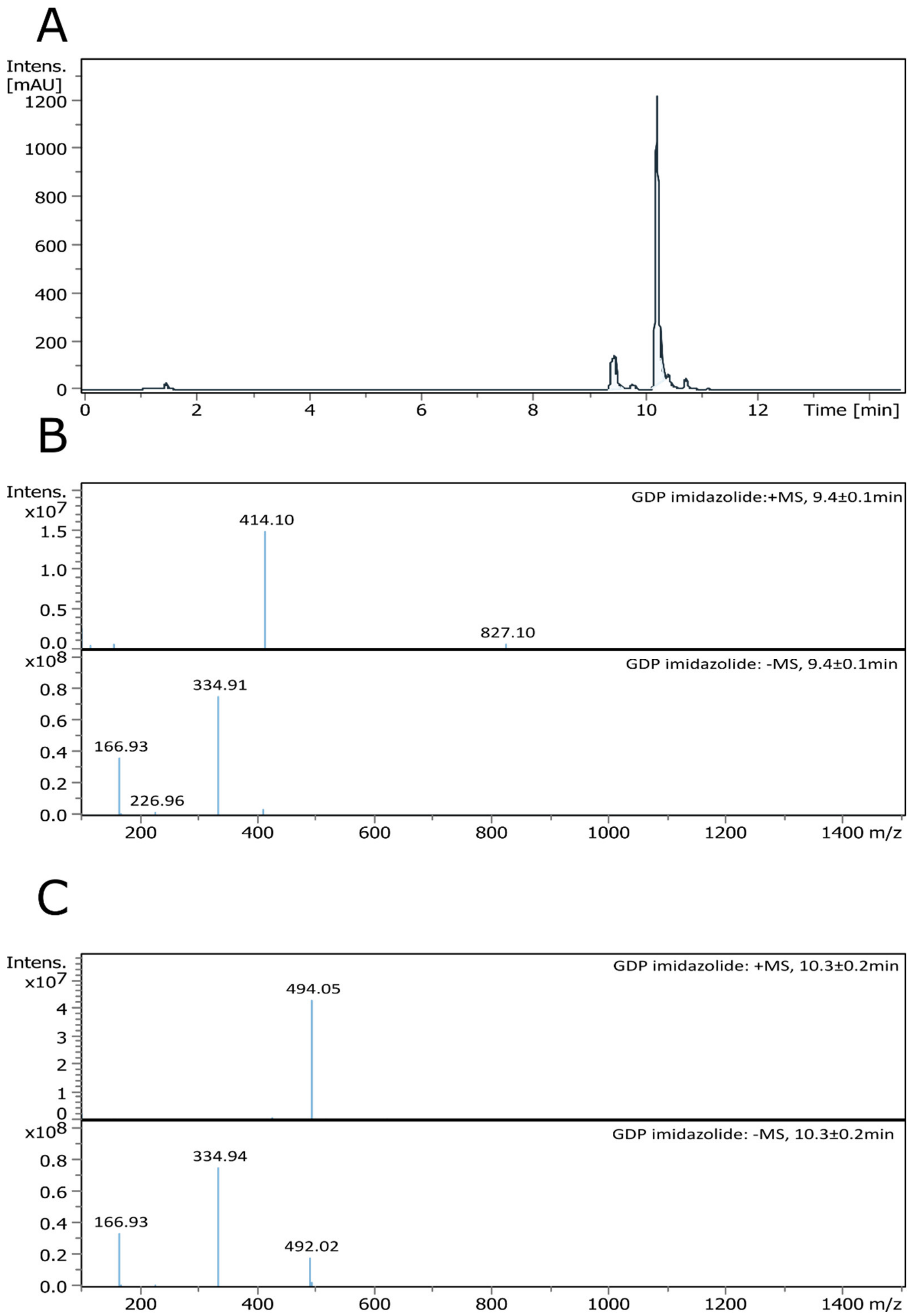

Figure S 7 LC-MS Chromatogram and Mass Spectra of GDP-imidazolide A: Chromatogramm, B: Mass spectra at min 9 C: Mass spectra at min 10. The $[M+H]^+$ = 494.05 corresponds to the diphosphate, $[M+H]^+$ = 414.40 to the monophosphate.

2.8. Synthesis of ZipCap **1a**

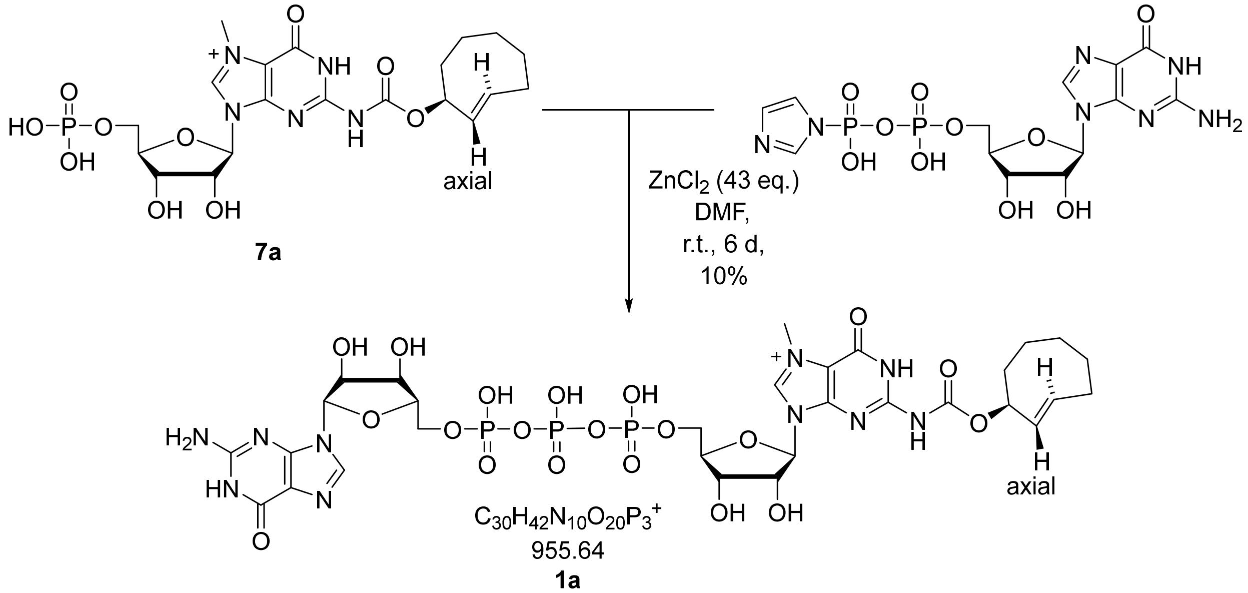

$ZnCl_2$ (3.80 g, 28.4 mmol, 43 equiv.) was heated *in vacuo* to 180 °C for 6 h. After cooling to r.t. under argon, GDP imidazolide (977 mg, 1.98 mmol, 3.0 equiv.) and monophosphate **7a** (350 mg, 0.660 mmol, 1.0 equiv.) were added, followed by DMF (10.0 mL). The reaction mixture was stirred at r.t. for 6 d.

The suspension was cooled to 0 °C, and a solution of TEAB (1 M in H_2O, 10.0 mL) and EDTA (2 M in H_2O, 10.0 mL, pH = 8) were added simultaneously. After stirring for 5 min, the mixture was directly subjected to reversed-phase flash chromatography (C18, 25 g, H_2O + 0.1 M TEAB:ACN = 0–50% over 20 column volumes). The fractions containing the product were combined, and the solvent was removed *in vacuo*.

The product was further purified by preparative HPLC (BEH HILIC column, H_2O + 5 mM TEAA:ACN = 90–50% over 30 column volumes). **1a** (66 mg, 0.070 mmol, 10%) was obtained as a colorless solid.

LC–MS (ESI): calculated for $[M]^+$ = 955.18; found: m/z = 955.18.

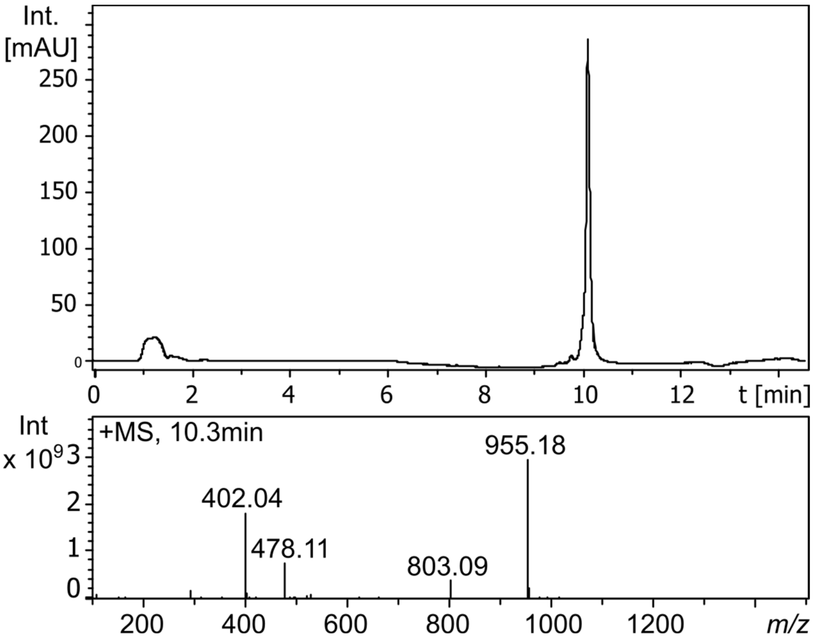

Figure S 8 LC-MS analysis of **1a.**

2.9. Synthesis of ZipCap **1e**

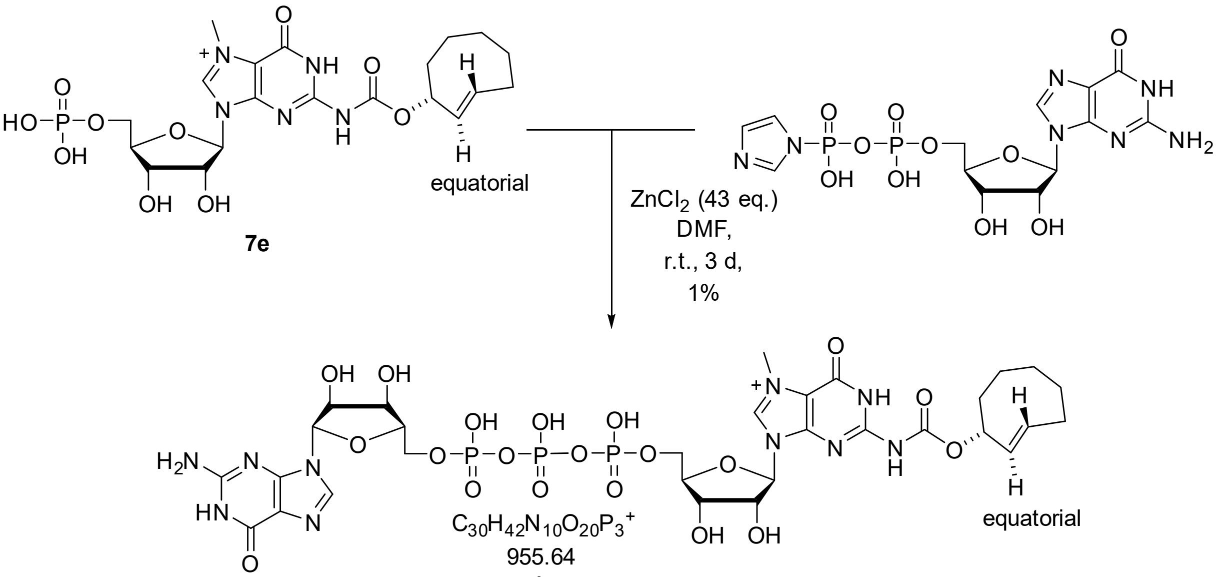

$ZnCl_2$ (545 mg, 5.20 mmol, 43.0 equiv.) was heated *in vacuo* to 180 °C for 6 h. After cooling to r.t. under argon, GDP imidazolide (138 mg, 0.280 mmol, 3.0 equiv.) was added. In a separate flask, monophosphate **7e** (50 mg, 0.093 mmol, 1.0 equiv.) was suspended in DMF (3.0 mL) and transferred to the GDP imidazolide/$ZnCl_2$ mixture. The reaction mixture was stirred at r.t. for 3 d.

The suspension was cooled to 0 °C, and TEAB (1 M, 2 mL) and a solution of EDTA (2 M in H_2O, 2.0 mL, pH = 8) were added simultaneously. After stirring for 5 min, the

mixture was directly subjected to reversed-phase flash chromatography (C18, 25 g, H_2O + 0.1 M TEAB:ACN = 0–50% over 20 column volumes). The fractions containing the product were combined, and the solvent was removed *in vacuo*.

The product was further purified by preparative HPLC (C18, H_2O + 0.1 M TEAB:ACN = 10–20% over 50 column volumes), followed by a second preparative HPLC purification (BEH HILIC column, H_2O + 5 mM TEAA:ACN = 90–50% over 30 column volumes). **1e** (1 mg, 1.05 µmol, 1%) was obtained as a white solid.

HR MS (ESI): calculated for $[M]^+$ = 955.1784194; found: m/z = 955.17899.

2.10. Synthesis of TCOs **3a** and **3e**

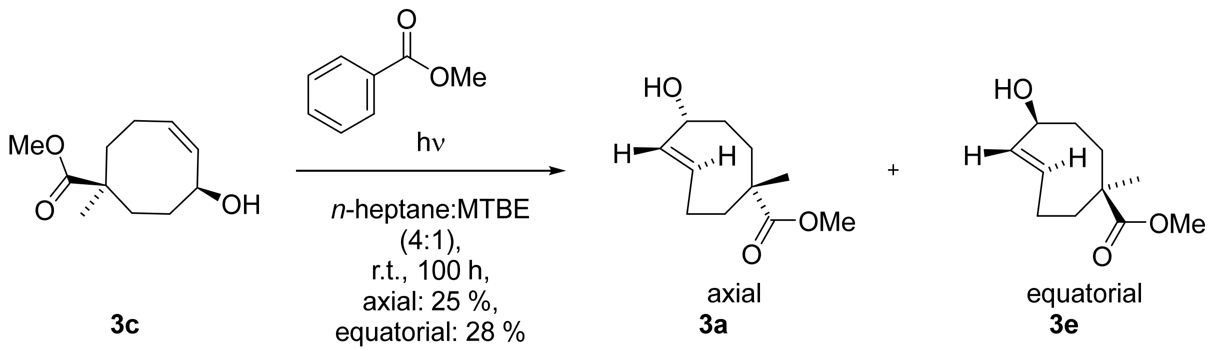

3c was synthesized according to Thurecht *et al.*.[25]

Ester **3c** (1.2 g, 6.1 mmol, 1.0 equiv.) and methyl benzoate (2.2 mL, 2.5 g, 18.2 mmol, 3.0 equiv.) were dissolved in *n*-heptane:MTBE (4:1, 400 mL). The solution was irradiated for 100 h while continuously passed through a column filled with silica/$AgNO_3$ (10:1, 25 g). The column was kept in the dark during irradiation. Subsequently, the column was eluted with CH_2Cl_2 (400 mL) to recover unreacted starting material.

The silica/$AgNO_3$/*trans*-cyclooctene mixture was transferred into an Erlenmeyer flask and stirred with CH_2Cl_2 (250 mL) and aqueous ammonia (conc., 50 mL) at room temperature for 15 min. The solids were filtered off and the layers were separated. The aqueous layer was extracted with CH_2Cl_2 (3 × 150 mL), and the combined organic layers were dried over $MgSO_4$. The solvent was removed *in vacuo*, and the crude product was purified by column chromatography (SiO_2, *c*-Hex:EtOAc = 4:1).

3a (axial isomer, 300 mg, 1.5 mmol, 25 %) and **3e** (equatorial isomer, 347 mg, 1.7 mmol, 28 %) were obtained as colorless oils.

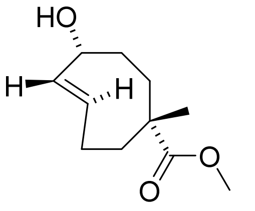

(±)
axial
$C_{11}H_{18}O_3$
198.26
3a

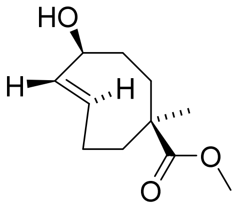

(±)
equatorial
$C_{11}H_{18}O_3$
198.26
3e

The axial/equatorial assignment is based on the stereochemistry of the hydroxy group.

Axial isomer:

TLC: R_f = 0.33 (SiO_2, *c*-Hex:EtOAc = 4:1).

^{1}H NMR (500 MHz, $CDCl_3$) δ [ppm] = 6.06 (ddd, *J* = 15.9, 11.1, 3.4 Hz, 1H), 5.63 (dd, *J* = 16.6, 2.1 Hz, 1H), 4.47 (br. s, 1H), 3.63 (s, 3H), 2.33–2.28 (m, 1H), 2.26–2.20 (m, 1H), 2.18–2.11 (m, 1H), 1.98–1.79 (m, 2H), 1.60–1.52 (m, 2H), 1.51 (br. s, 1H), 1.11 (s, 3H).

^{13}C NMR (126 MHz, $CDCl_3$) δ [ppm] = 180.5, 135.1, 130.6, 69.8, 52.1, 45.0, 44.7, 38.3, 31.0, 29.8, 18.3.

Equatorial isomer:

TLC: R_f = 0.30 (SiO_2, *c*-Hex:EtOAc = 4:1).

^{1}H NMR (500 MHz, $CDCl_3$) δ [ppm] = 5.79 (ddd, *J* = 15.9, 11.6, 4.0 Hz, 1H), 5.36 (dd, *J* = 16.2, 9.5 Hz, 1H), 4.23–4.18 (m, 1H), 3.73 (s, 3H), 2.70–2.68 (m, 1H), 2.28–2.21 (m, 1H), 2.15–2.07 (m, 2H), 1.57–1.45 (m, 1H), 1.78 (dd, *J* = 6.5, 4.1 Hz, 1H), 1.52 (ddd, *J* = 14.2, 12.6, 6.8 Hz, 1H), 1.34–1.28 (m, 1H), 1.20 (s, 3H).

^{13}C NMR (126 MHz, $CDCl_3$) δ [ppm] = 177.4, 135.0, 132.8, 75.1, 51.5, 47.6, 46.1, 40.0, 38.5, 34.8, 31.1.

HR MS (PCI): calculated for $[M-H_2O+H]^+$ = 181.12231; found: m/z = 181.1222.

2.11. Synthesis of TBS-protected N^2-TCO_{eq}-guanosine **6e**

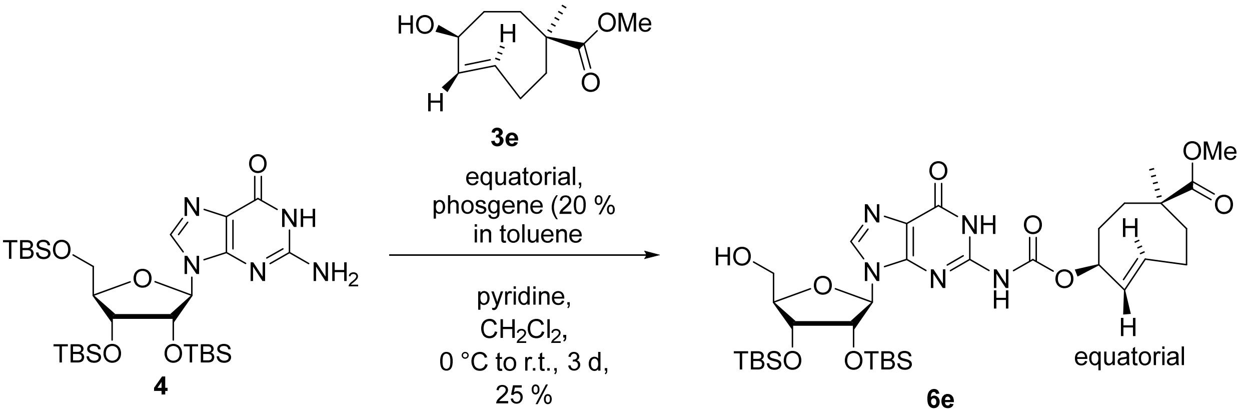

In an inert atmosphere, protected guanosine TBS-G **4** (626 mg, 1.00 mmol, 1.0 equiv.) and pyridine (169 µL, 166 mg, 16.5 mmol, 2.1 equiv.) were dissolved in CH_2Cl_2 (7.5 mL). The solution was cooled to 0 °C and phosgene (20% in toluene, 198 mg, 2.0 mmol, 2.0 equiv., corresponding to a total amount of 1.1 mL, 989 mg) was added dropwise. The solution was stirred for 30 min at 0 °C. Afterwards, a solution of ester **3e** (300 mg, 1.50 mmol, 1.5 equiv.) in CH_2Cl_2 (2.5 mL) was added dropwise. The solution was stirred for 3 d to gradually warm up to room temperature. The volatiles were removed *in vacuo* and the crude product was purified by flash-RP (C18, H_2O + 0.1% FA:ACN = 0–100% in 20 column volumes). The fractions containing the product were combined and the solvent was removed *in vacuo*. TCO-functionalized guanosine **6e** (180 mg, 0.250 mmol, 25%) was obtained as a colorless solid.

Note: There was no evidence in 2D NMR that the location of TBS-cleavage is the 5' position of the ribose. However, due to increased steric accessibility of the 5' position, it was assigned as presented here.

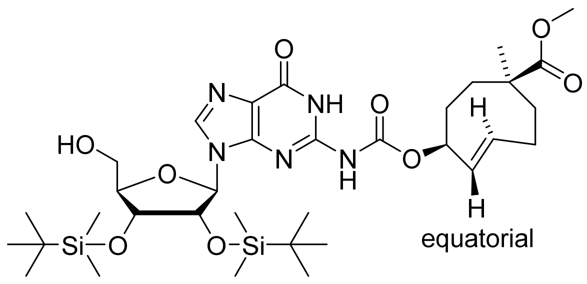

$C_{34}H_{57}N_5O_9Si_2$
736.03
6e

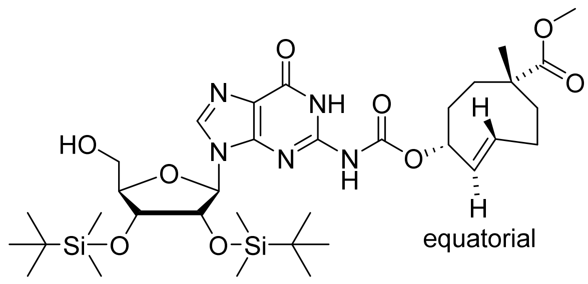

$C_{34}H_{57}N_5O_9Si_2$
736.03
6e

^{1}H NMR (500 MHz, $CDCl_3$) δ [ppm] = 11.42 (br. s, 1H), 7.69–7.68 (m, 1H), 7.63 (br. s, 1H), 5.98 (ddd, *J* = 15.9, 11.6, 4.1 Hz, 1H), 5.69 (d, *J* = 7.8 Hz, 1H), 5.37 (dd, *J* = 16.2, 9.7 Hz, 1H), 5.23–5.17 (m, 1H), 4.73–4.68 (m, 1H), 4.23 (m, 1H), 4.14 (m, 1H) 3.92 (dd, *J* = 12.7, 2.3 Hz, 1H), 3.76 (s, 3H), 3.70–3.66 (m, 1H), 2.76–2.69 (m, 1H), 2.32–2.26 (m, 1H), 2.22–2.14 (m, 3H), 1.84 (dd, *J* = 15.5, 5.8 Hz, 1H), 1.61–1.53 (m, 2H), 1.40–1.33 (m, 1H), 1.22 (s, 3H), 0.93 (s, 9H), 0.78–0.76 (m, 9H), 0.12–0.10 (m, 6H) −0.10– −0.11 (m, 3H), −0.47– −0.59 (m, 3H).

^{13}C NMR (126 MHz, $CDCl_3$) δ [ppm] = 177.1, 155.1, 152.9, 152.9, 147.0, 146, 139.2, 135.7, 135.7, 130.2, 130.2, 122.9, 122.9, 90.7, 90.7, 88.7, 79.8, 74.4, 74.3, 73.9, 63.0, 51.6, 47.3, 45.9, 38.5, 38.5, 36.0, 34.8, 31.2, 25.9, 25.8, 18.2, 17.9, −4.4, −4.5, −5.6.

Multiple overlaps in ^{1}H and ^{13}C NMR due to diastereomer formation.

HR MS (ESI): calculated for $[M+Na]^+$ = 758.3587024; found: m/z = 758.35883.

2.12. Synthesis of TBS-protected N^2-TCO$_{ax}$-guanosine **6a**

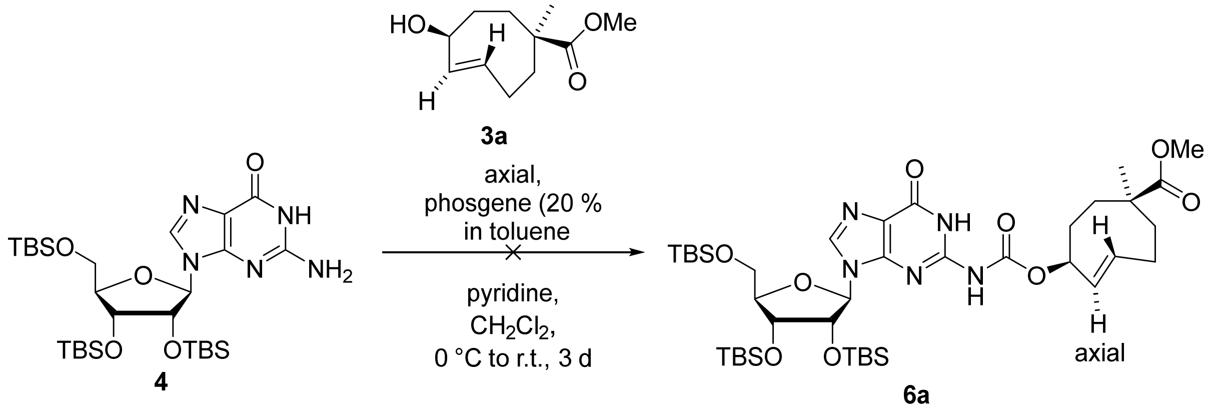

The reaction was performed analogously to 2.11.

The zoomed area (Figure S 9: 1H NMR spectrum of 6a. The zoomed area shows the cis signals of the double bond.) shows the relevant area of the signals of the double bond of the eight membered ring.

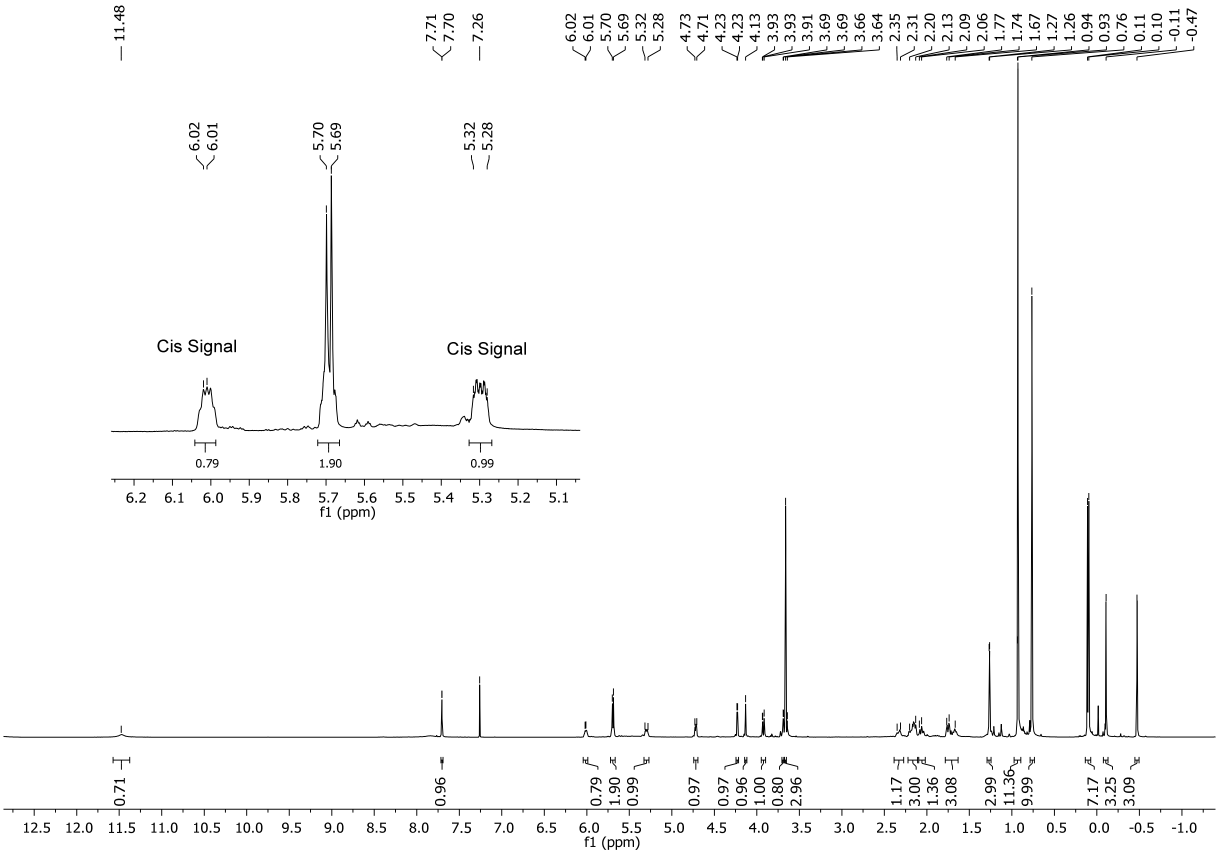

Figure S 9: ^{1}H NMR spectrum of **6a**. The zoomed area shows the *cis* signals of the double bond.

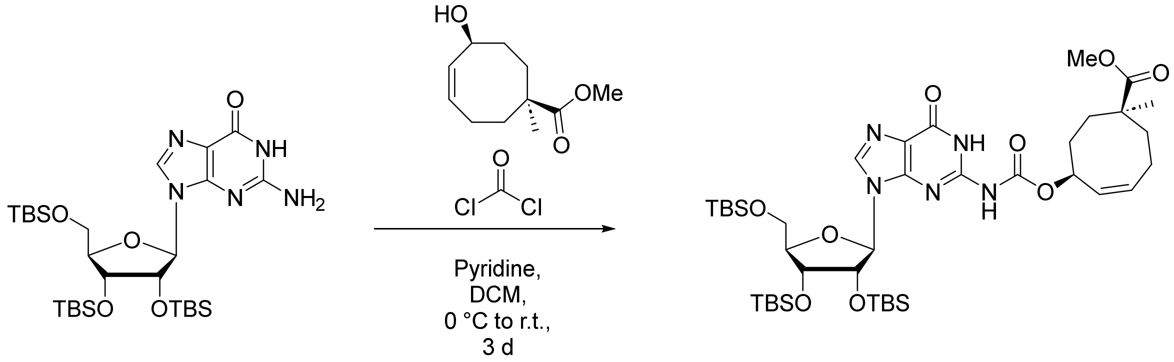

This reaction was performed during a preliminary screening reaction in analogy to 2.11. The corresponding NMR is presented in Figure S 10. The zoomed area presents the *cis* signals of the double bond of the eight-membered ring (CCO).

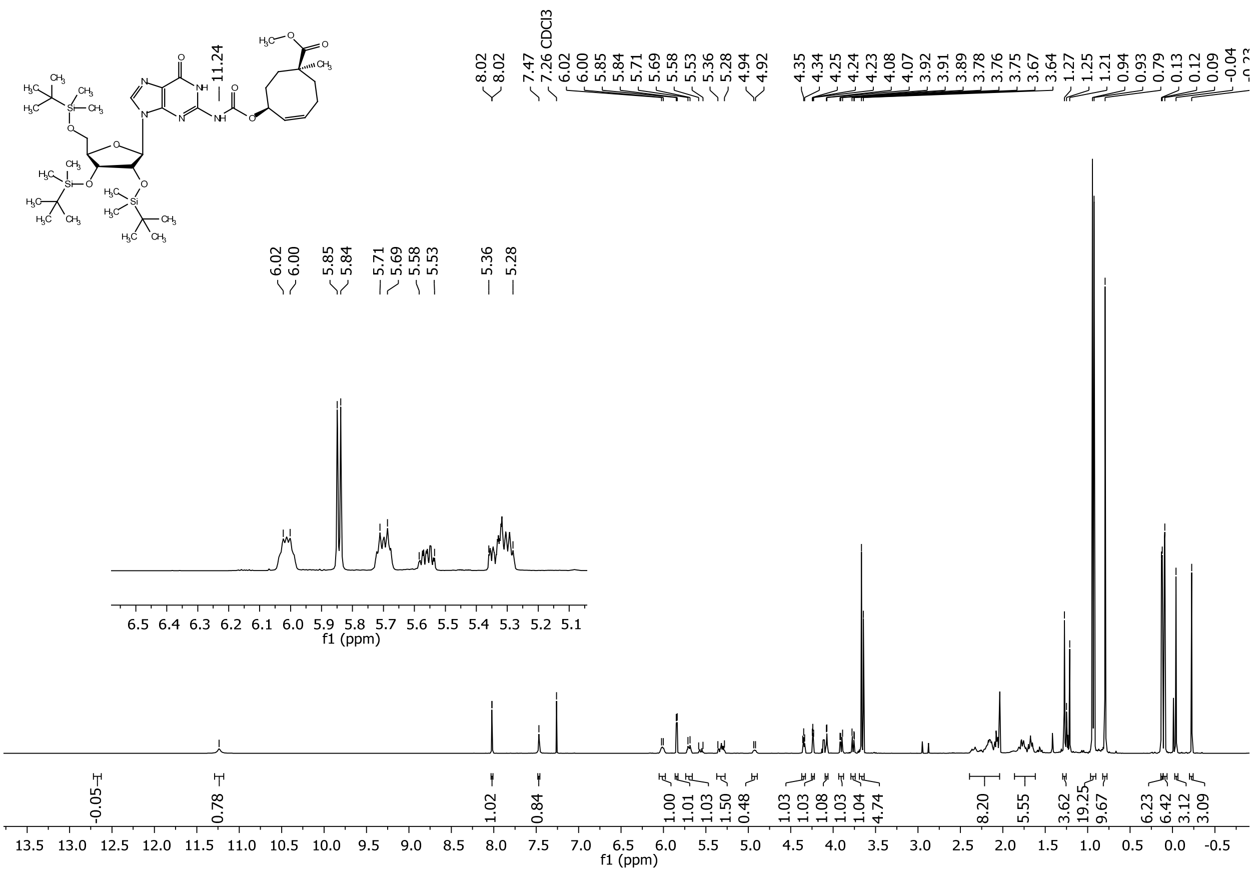

Figure S 10 ^{1}H-NMR of the addition of **3c** to **4** during a preliminary screening reaction.

2.13. Synthesis of Tetrazine **8**

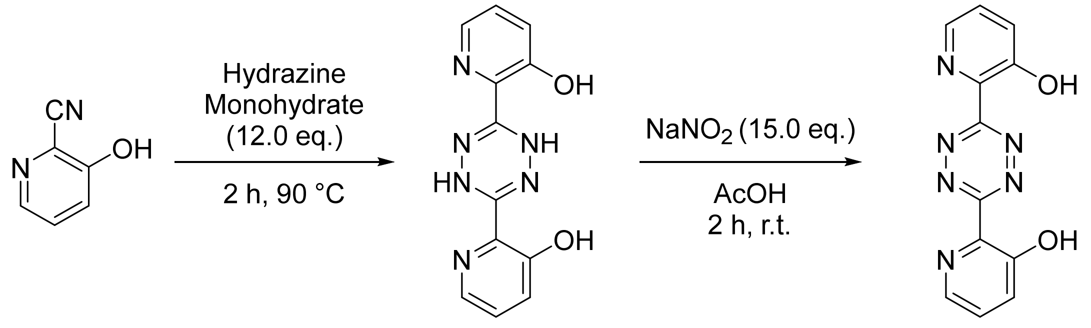

dihydro-Tz **8**,Tz

Aromatic alcohol (1.0 g, 8.3 mmol 1.0 equiv.) and hydrazine monohydrate (5.6 mL, 98 mmol, 12.0 equiv.) were stirred for 2 h at 90 °C. After allowing the mixture to cool to room temperature, EtOH (20 mL) was added and the mixture was stirred for 5 min, after which the suspension was filtered and washed with EtOH (3 x 20 mL). The residue was dried *in vacuo*. The intermediate dihydro-Tz (400 mg, 1.5 mmol) was suspended in AcOH (40 mL, excess) and $NaNO_2$ (0.5 g, 7.4 mmol, 5.0 eq.) in H_2O (10 mL) was added in small portions. After 1 h, $NaNO_2$ (1.0 g, 14.8 mmol, 10.0 eq.) was added in small portions and the mixture was stirred for another 2 h at room temperature. $CHCl_3$ (100 mL) and H_2O (80 mL) were added and the layers were separated. The aqueous layer was extracted with $CHCl_3$ (2 × 100 mL) was the combined organic layers dried over $MgSO_4$. The solvent was removed in vacuo and the residue purified *via* column chromatography (SiO_2, CH_2Cl_2:MeOH = 10:1). Tetrazine **8** (292 mg, 1.1 mmol, 27 %) was obtained as a red solid.

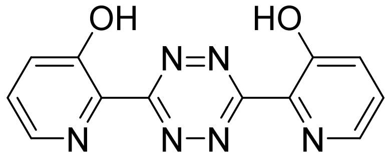

$C_{12}H_8N_6O_2$
268.24

^{1}H NMR (400 MHz, DMSO-d_6) δ [ppm] = 10.75 (br. S, 2H), 8.39-8.37 (m, 2H), 7.58-7.57 (m, 4H).

^{13}C NMR (101 MHz, DMSO-d_6) δ [ppm] = 164.3 (2C), 154.3 (2C), 141.3 (2C), 137.4 (2C), 127.6 (2C), 125.4 (2C).

HR MS (EI): calculated for $[M]^+$ = 268.0703; found: m/z = 268.0705. The analytical data are in accordance with literature.[21]

3 Prediction of physicochemical properties

Property prediction was performed using the Maestro 14.6.125 (2025-4) software by Schrödinger. Input structures for the literature-known tetrazines were generated using the built-in 2D-Skecher. The structures were pre-optimized using the ligand preparation tool with the OPLS_2005 force field without changing the ionization state.[26] The final predictions of physicochemical properties were performed using the QikProp module with default settings.[27]. The molecules were introduced by Mikula and Vrabel.[21],[22]

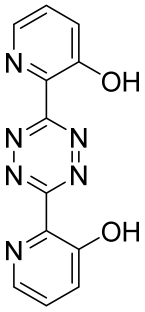

8
predicted Caco-2 permeability: 95,956

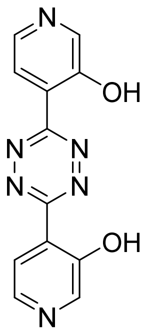

predicted Caco-2 permeability: 89,531

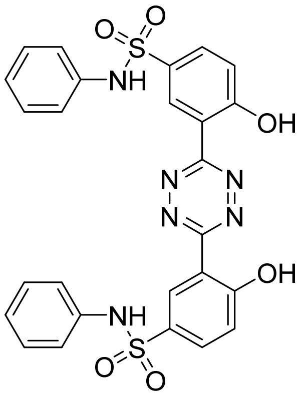

predicted Caco-2 permeability: 9,483

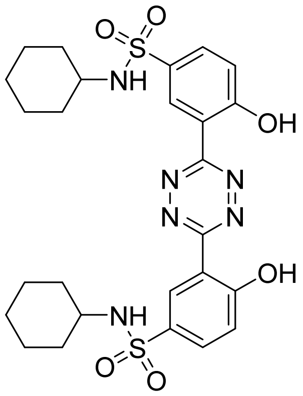

predicted Caco-2 permeability: 15,652

4 Release and stability assay **1a**

4.1. Release Assay of ZipCap **1a**

GDP imidazolide was obtained as a mixture of diphosphate and monophosphate species. Consequently, a minor fraction of the target structure was isolated as a mixture containing both diphosphate and triphosphate linkage. This fraction was used for the Click-to-Release assay as the phosphate linkage was not anticipated to influence the reactions outcome. The assay was performed on a 1 µmol scale with equimolar addition of the reactants and a total concentration of 10 mmol/L (1 µmol in 100 µL). Samples were drawn every 15 minutes over a course of 60 minutes.

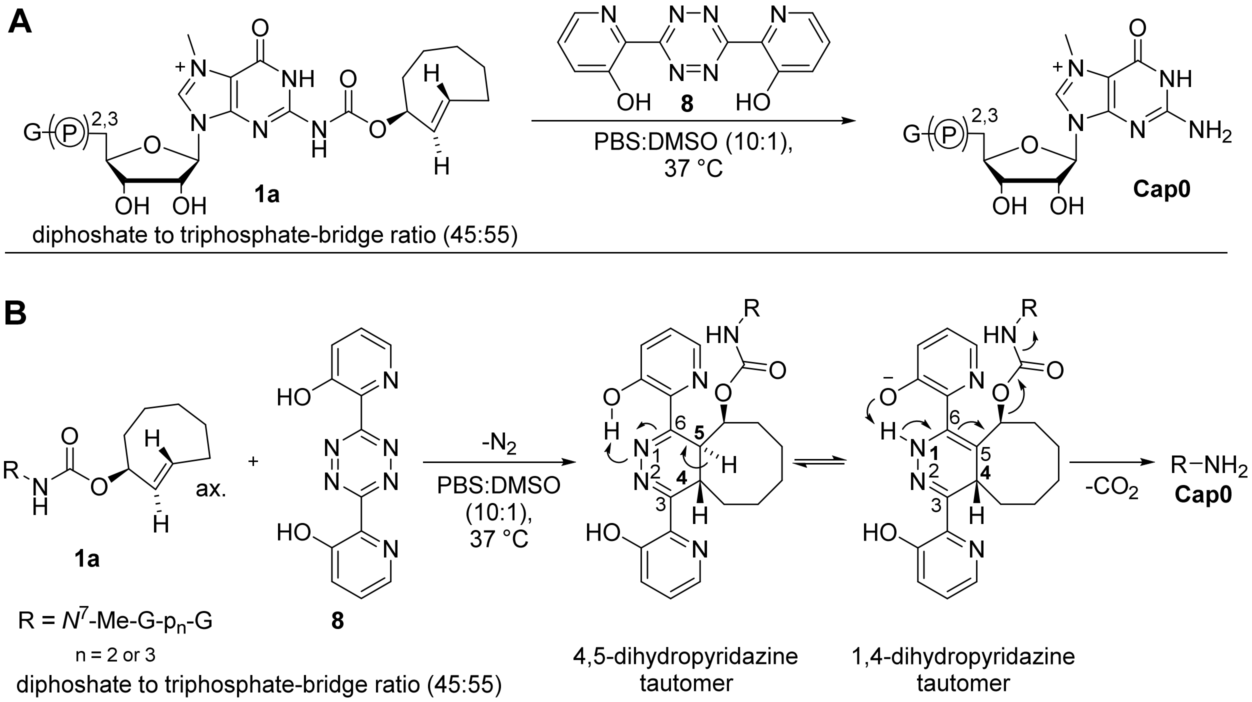

Scheme S 1 A: General depiction of the conditions of the Click-to-Release Assay. B: Mechanism of the Click-to-Release Reaction.

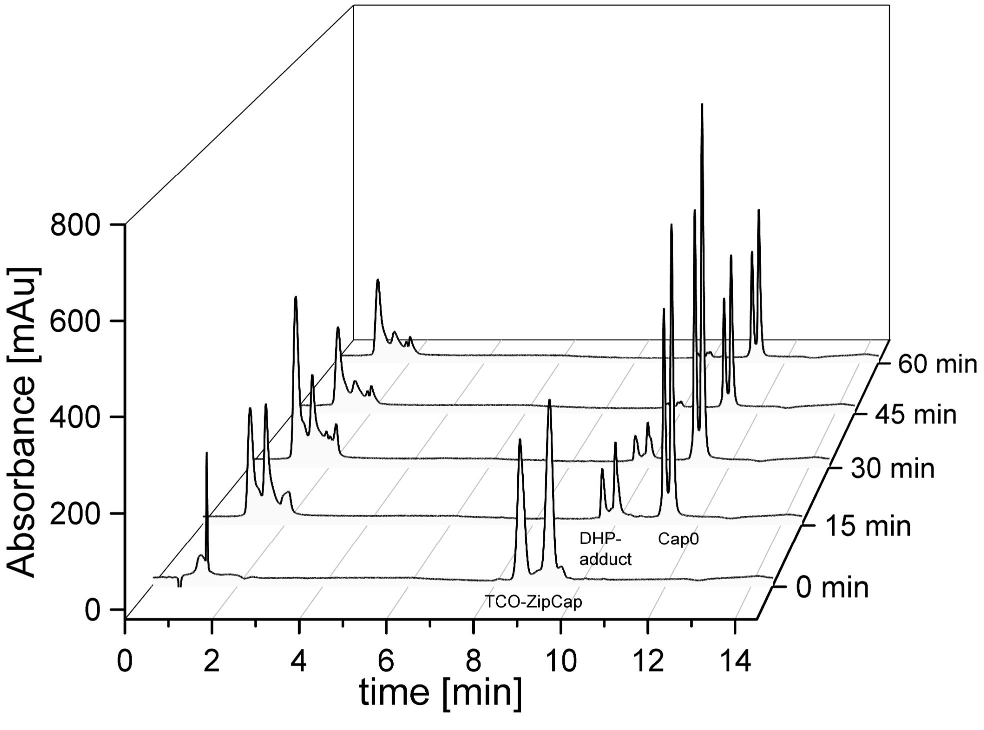

Figure S 11 Combined Waterfall Plot of the Release Assay

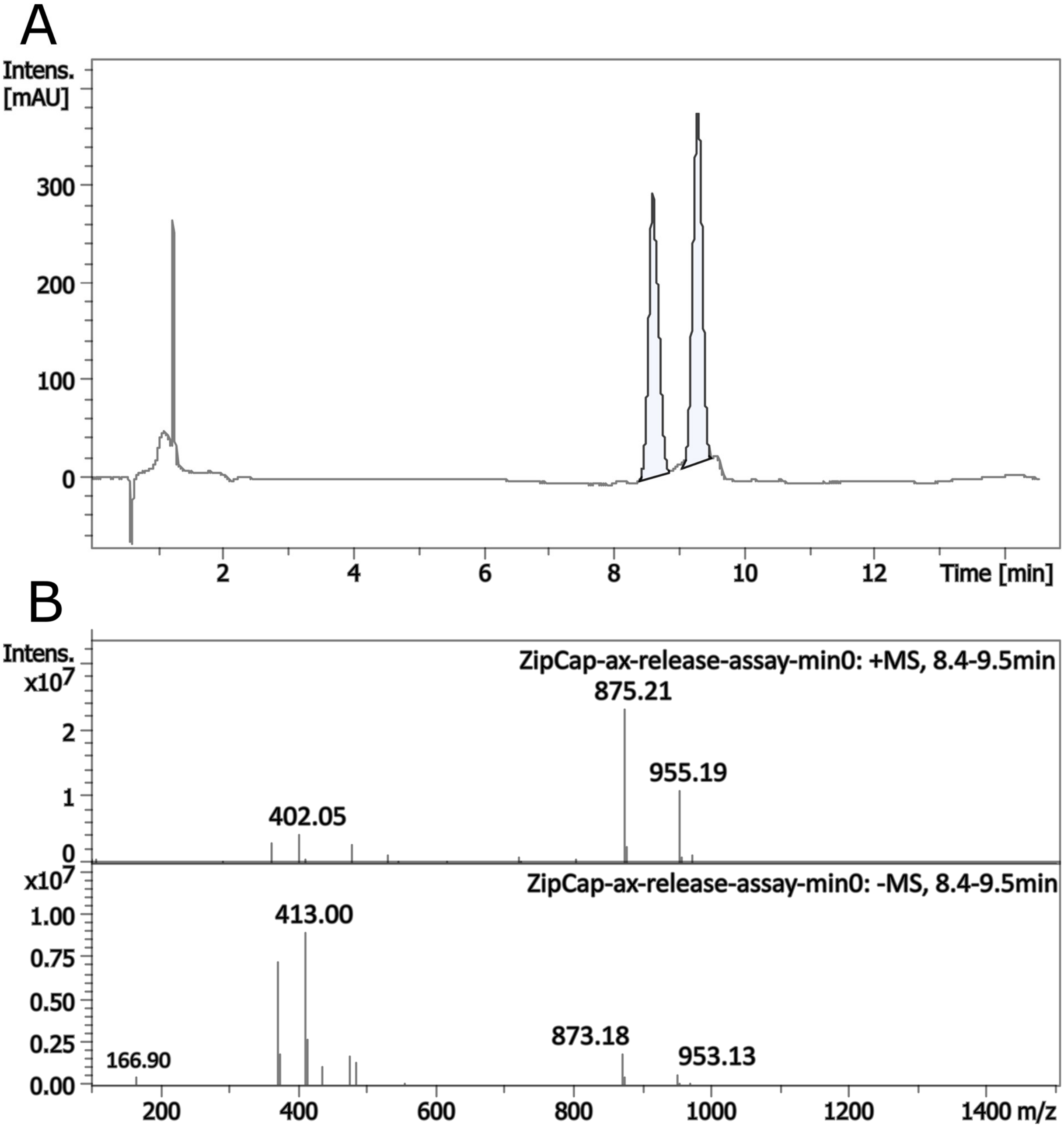

Figure S 12 LC-MS Chromatogram and Mass Spectra of ZipCap **1a** at min0. The mass 955.19 (pos. mode) and 953.13 (neg. mode) correspond to the ZipCap 1**a** containing a triphosphate linkage, whereas the masses 875.21 (pos. mode) and 873.18 (neg. mode) correspond to the diphosphate linkage.

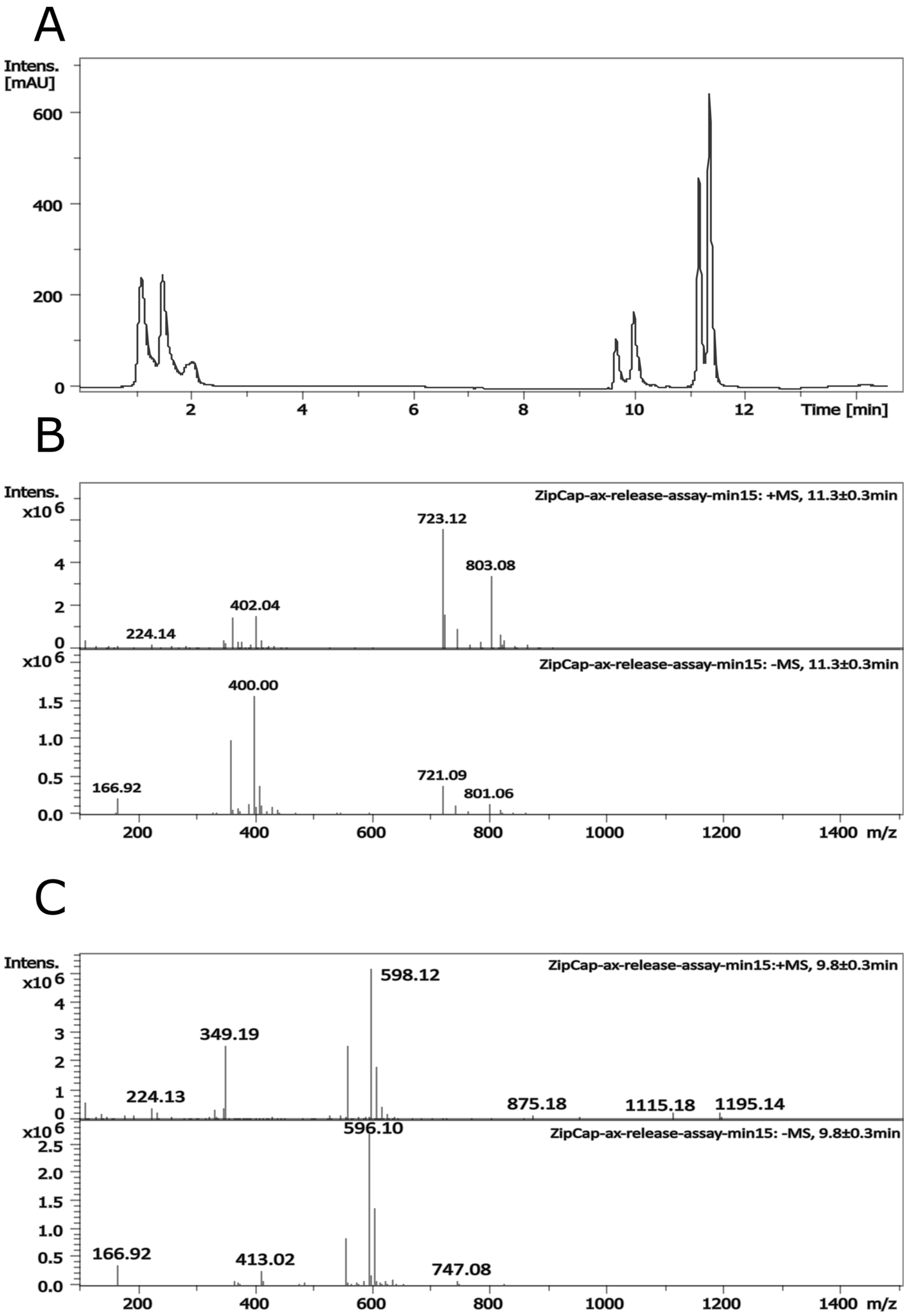

Figure S 13 A: Chromatogram of the Release Assay at min 15. B: Mass spectra (pos. and neg.) at min 11. Pos. mode: 803.08 corresponds to triphosphate linkage, 723.12 to the diphosphate linkage C: Mass spectra (pos. and neg.) at min 10. Pos mode: 1195.14 corresponds to the DHP adduct with triphosphate linkage, 1115.18 to the DHP adduct with diphosphate linkage.

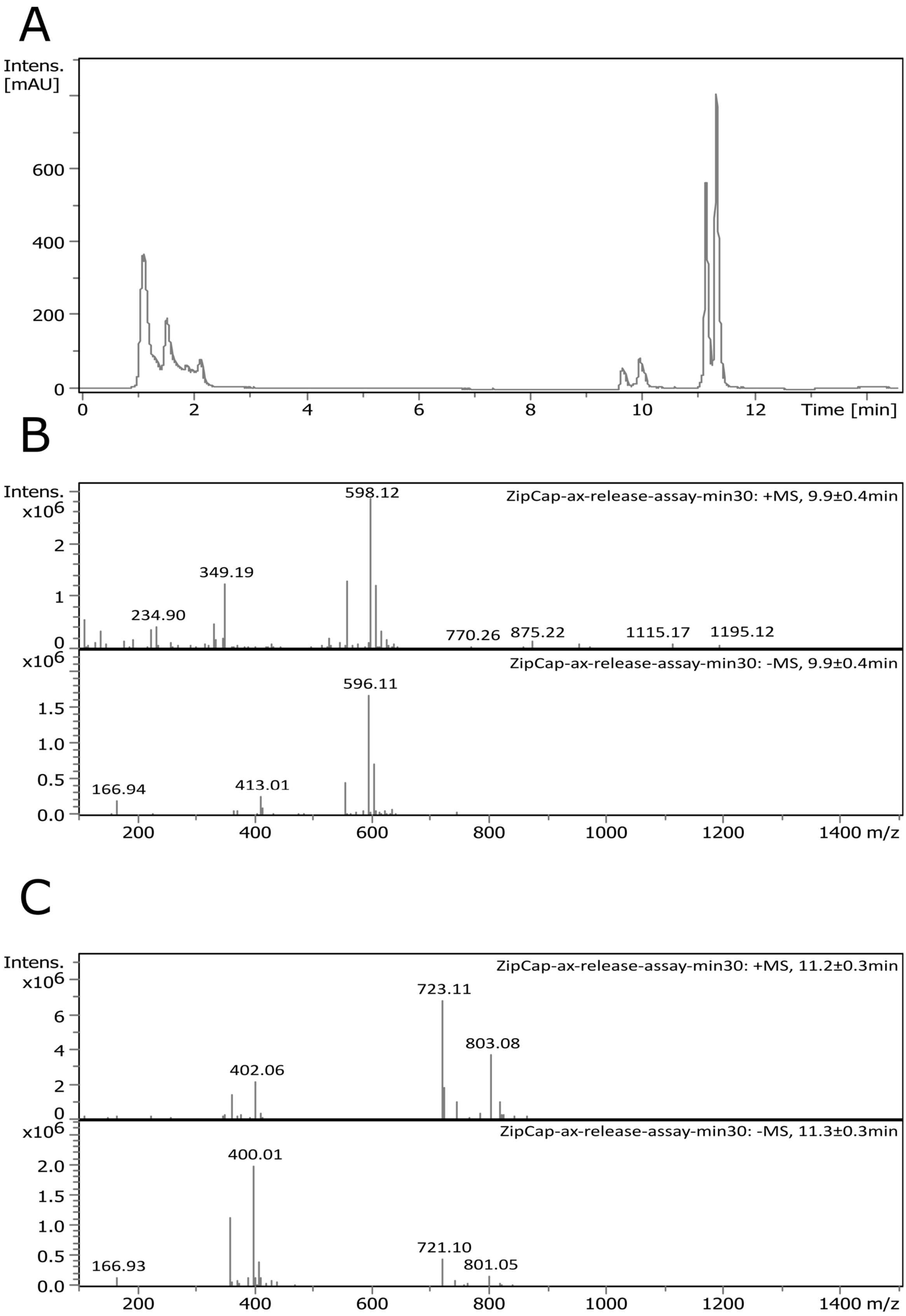

Figure S 14 A: Chromatogram of the Release Assay at min 30. B: Mass spectra (pos. and neg.) at min 10. Pos mode: 1195.12 corresponds to the DHP adduct with triphosphate linkage, 1115.17 to the DHP adduct with diphosphate linkage. C: Mass spectra (pos. and neg.) at min 11. Pos. mode: 803.08 corresponds to triphosphate linkage, 723.11 to the diphosphate linkage.

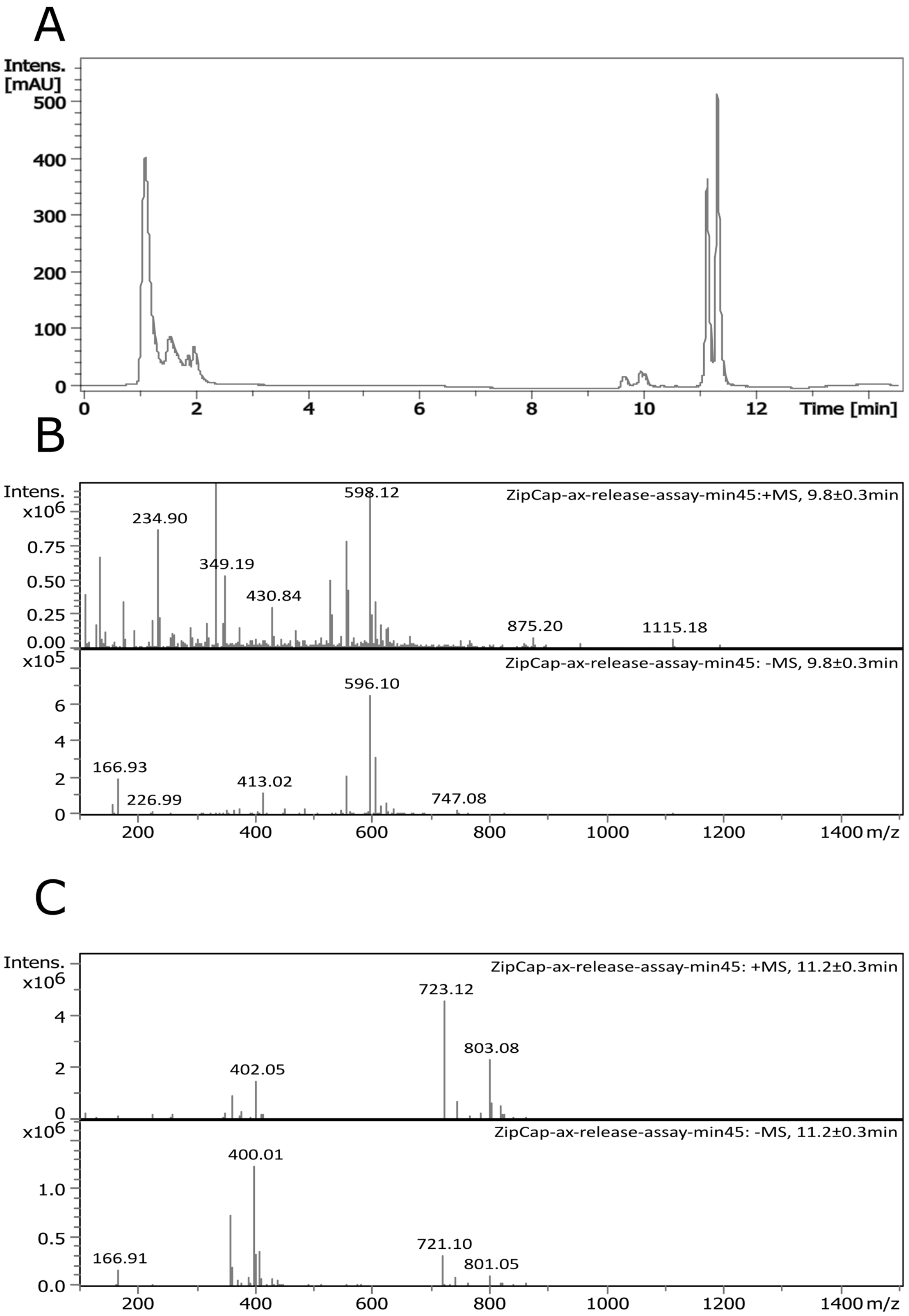

Figure S 15 A: Chromatogram of the Release Assay at min 45. B: Mass spectra (pos. and neg.) at min 10. Pos mode: 598.23 corresponds to the DHP adduct with triphosphate linkage $[m/2]^{2+}$, 1115.18 to the DHP adduct with diphosphate linkage. C: Mass spectra (pos. and neg.) at min 11. Pos. mode: 803.08 corresponds to triphosphate linkage, 723.12 to the diphosphate linkage.

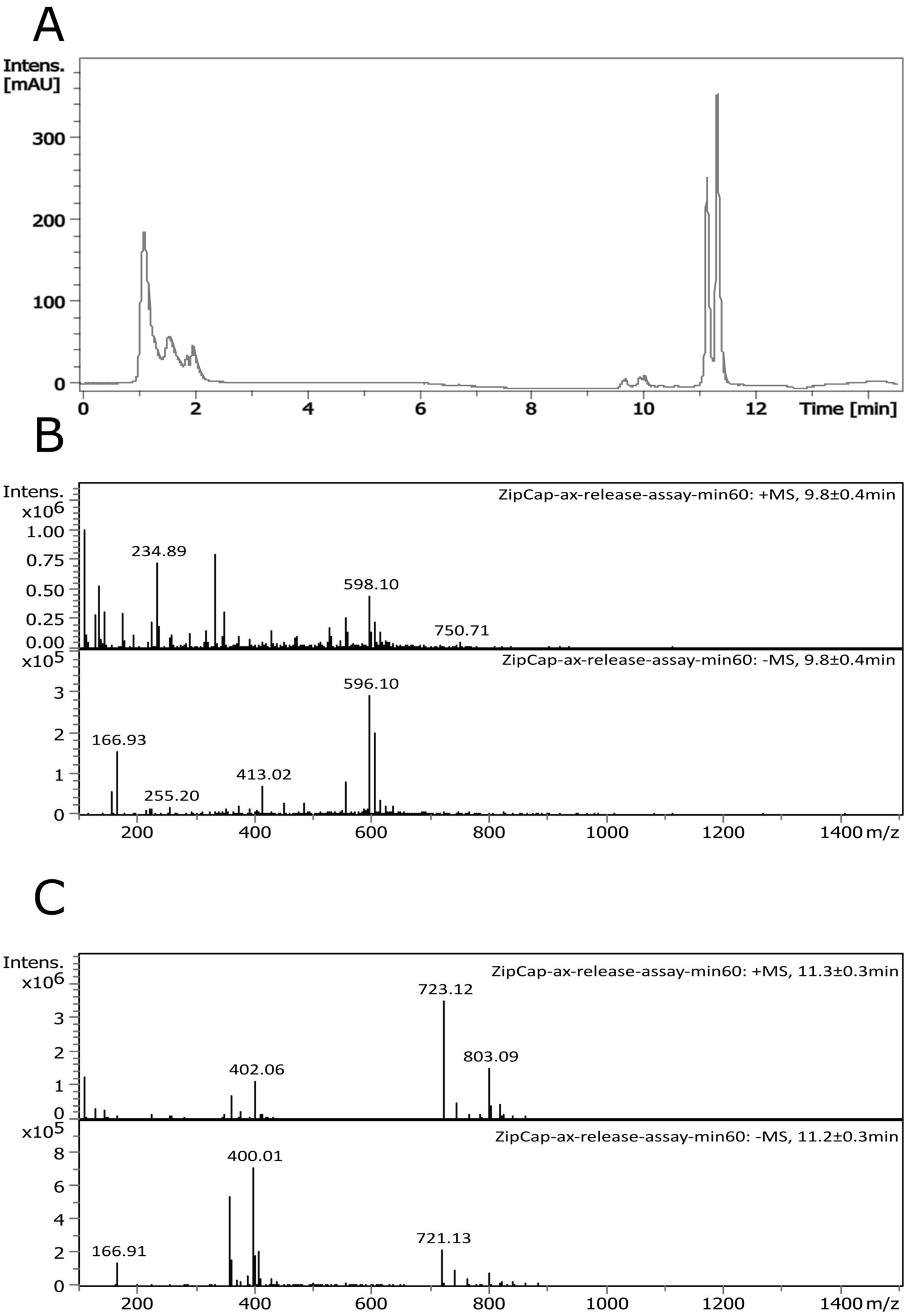

Figure S 16 A: Chromatogram of the Release Assay at min 60. B: Mass spectra (pos. and neg.) at min 10. Pos mode: 598.23 corresponds to the DHP adduct with triphosphate linkage $[m/2]^{2+}$ C: Mass spectra (pos. and neg.) at min 11. Pos. mode: 803.09 corresponds to triphosphate linkage, 723.12 to the diphosphate linkage.

4.2. Stability Assay of ZipCap **1a**

For the stability assay, 4 µL of a 40 mM stock solution of ZipCap **1a** (corresponding to 160 nmol or approximately 153 µg) were diluted in 10XT7 reaction buffer (composition not disclosed by the manufacturer, *New England Biolabs*, B0658AVIAL). For the +DTT condition, the reaction mixture additionally contained DTT at a final concentration of 5 mM (Figure S 18 Stability assay of **1a** including DTT, whereas the other assay (−DTT) was performed without DTT (Figure S 17 Stability assay of 1a without the addition of DTT). Incubation was carried out for a total of 240 min, and samples were drawn every 30 min for subsequent analysis by LC–MS.

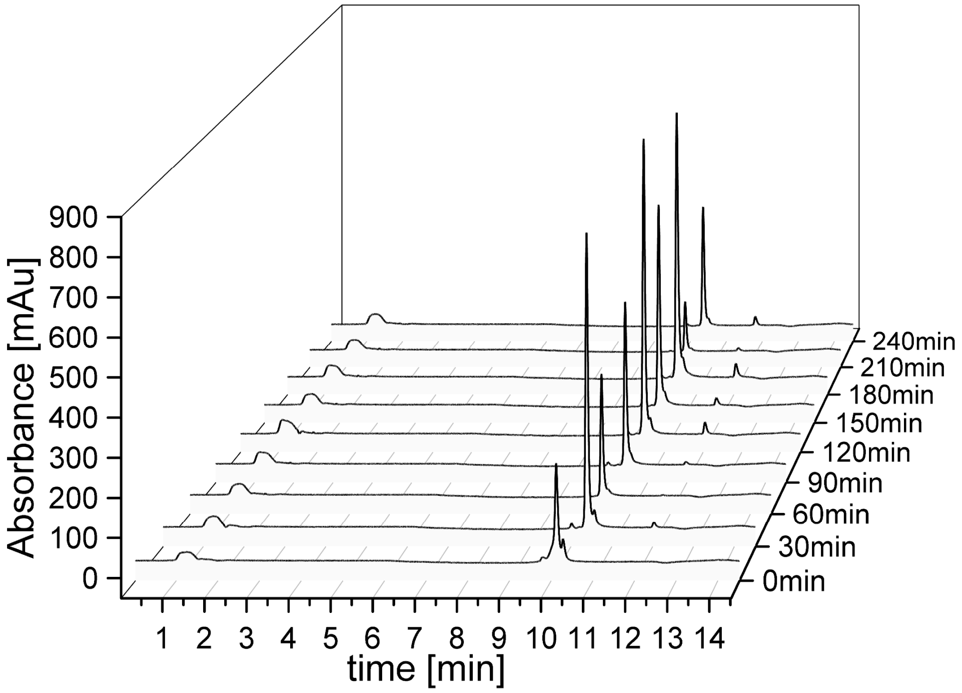

Figure S 17 Stability assay of **1a** without the addition of DTT. The minor additional signal at 12 min could not be unambiguously assigned based on its mass.

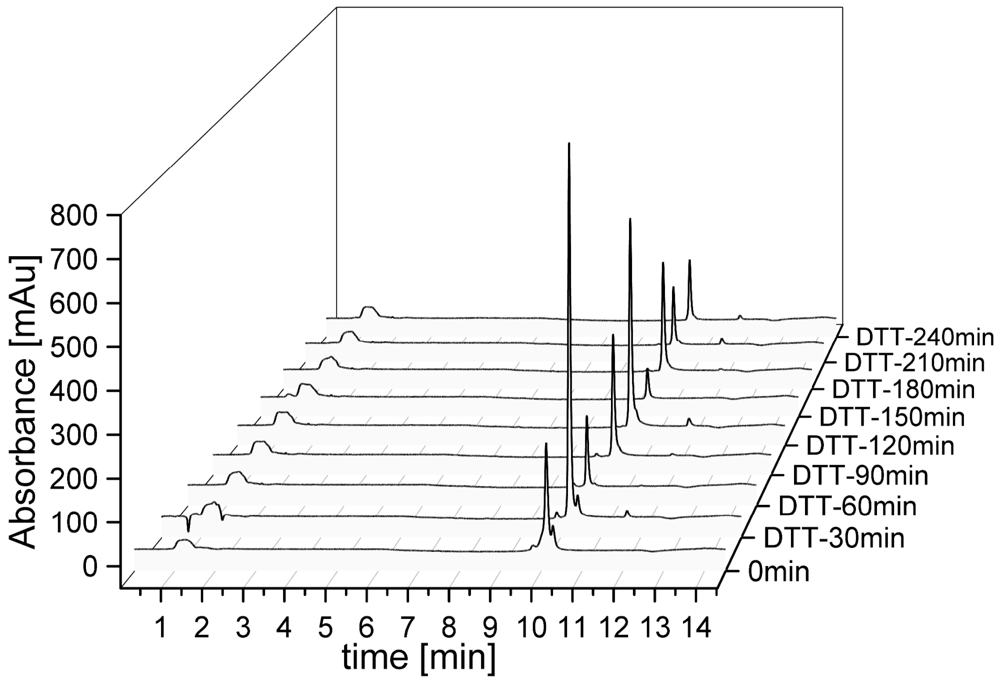

Figure S 18 Stability assay of 1**a** including DTT

5 NMR Spectra

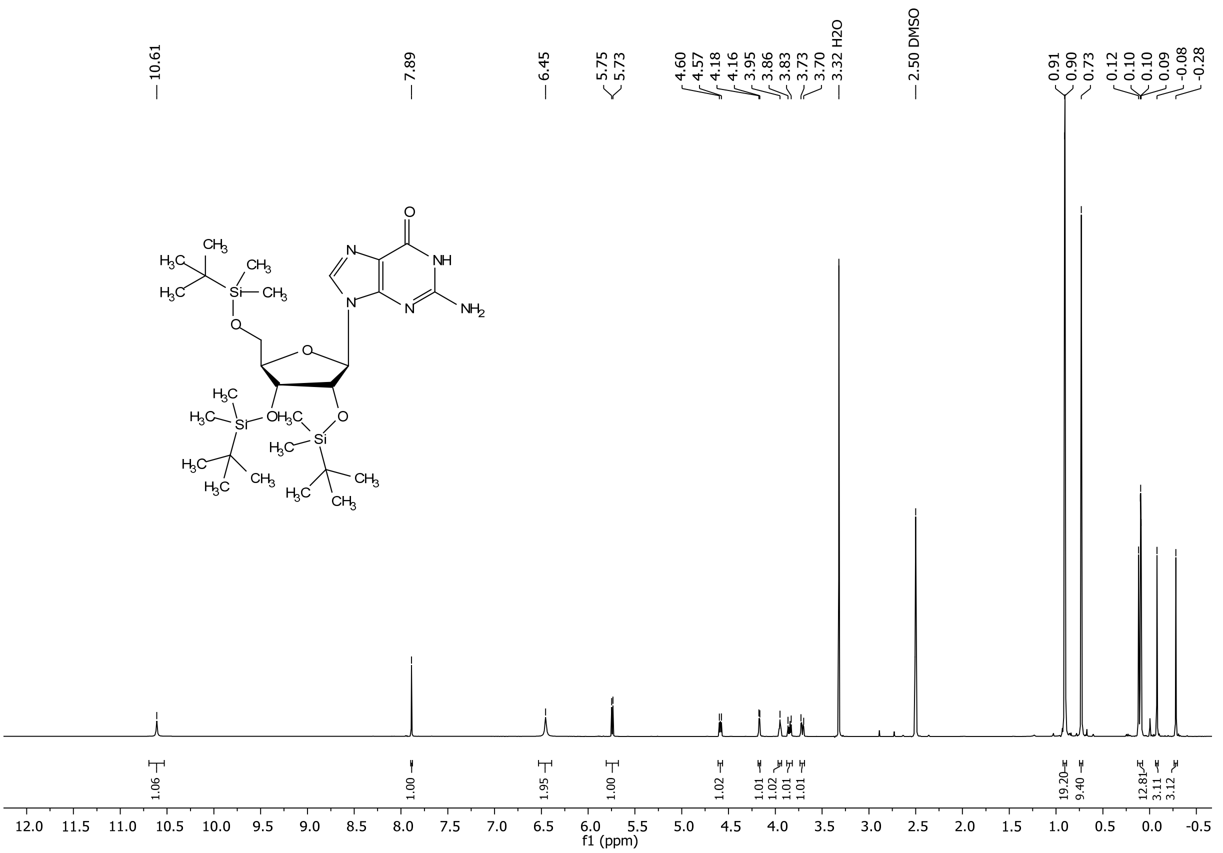

Figure S 19 ^{1}H NMR-spectrum (500 MHz, DMSO-d_6) of **4.**

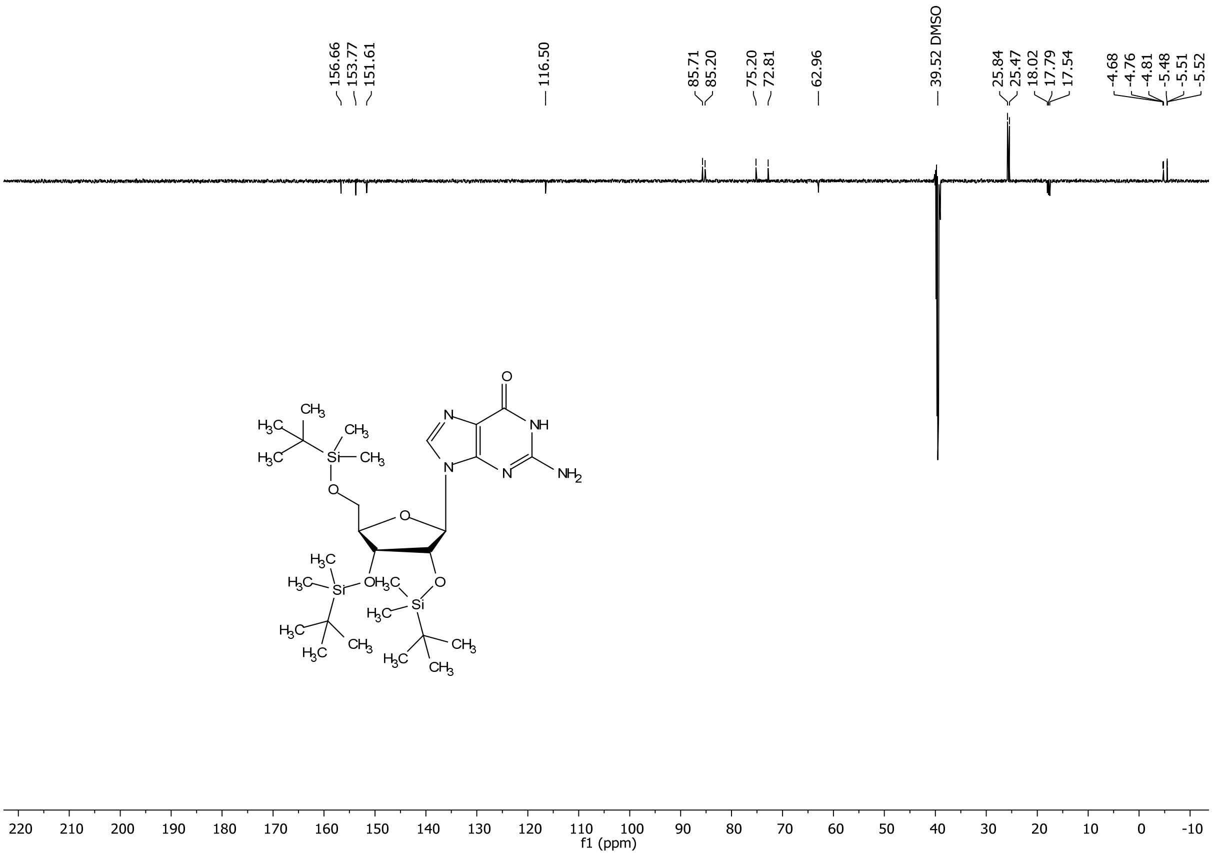

Figure S 20 ^{13}C NMR-spectrum (126 MHz, DMSO- d_6) of **4.**

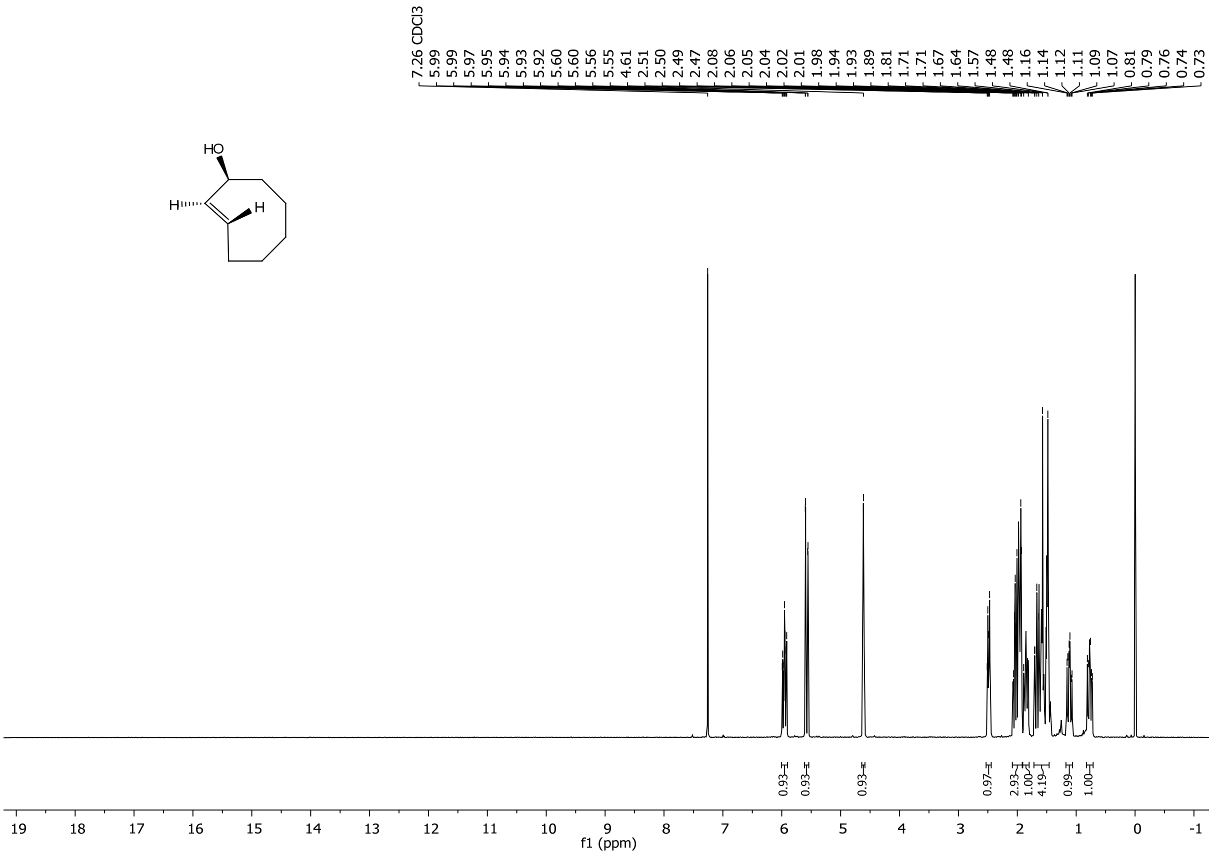

Figure S 21 ^{1}H NMR-spectrum (400 MHz, $CDCl_3$) of **2a.**

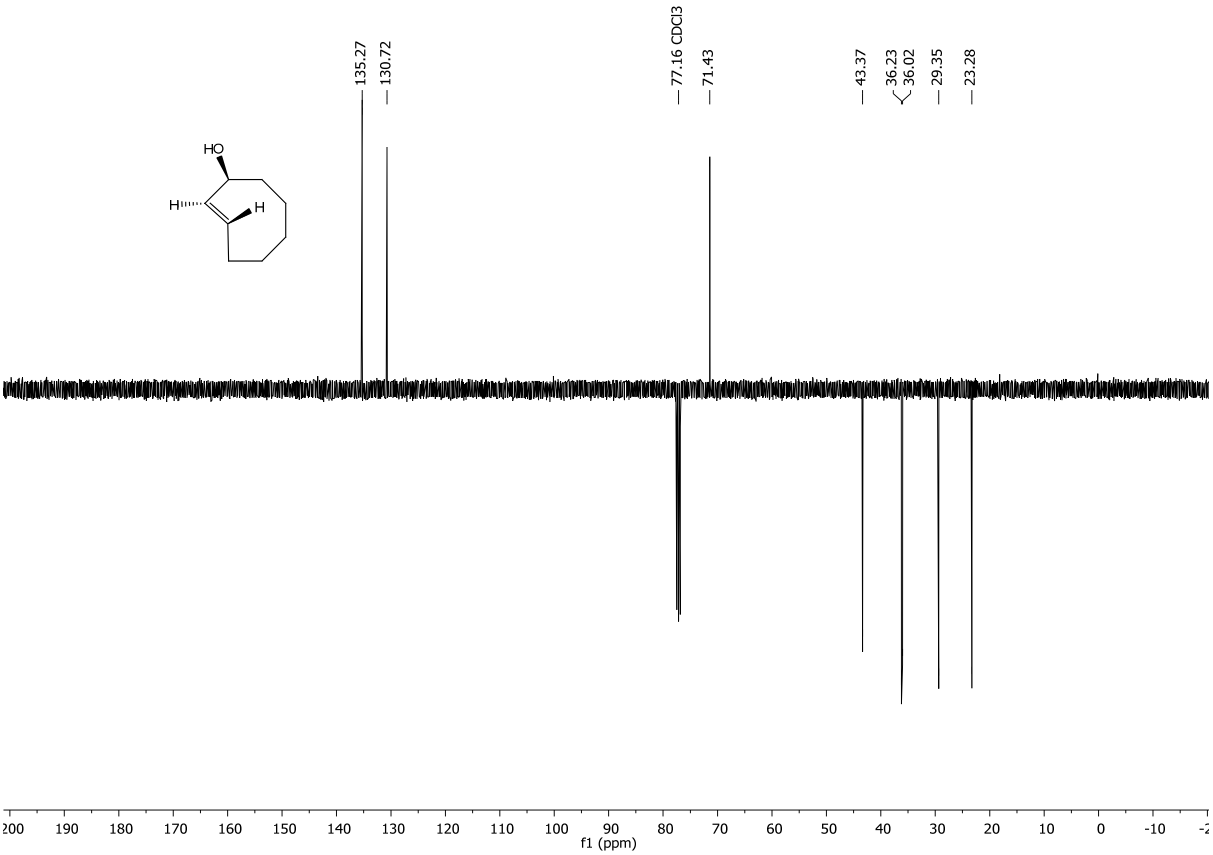

Figure S 22 ^{13}C NMR-spectrum (101 MHz, $CDCl_3$) of **2a.**

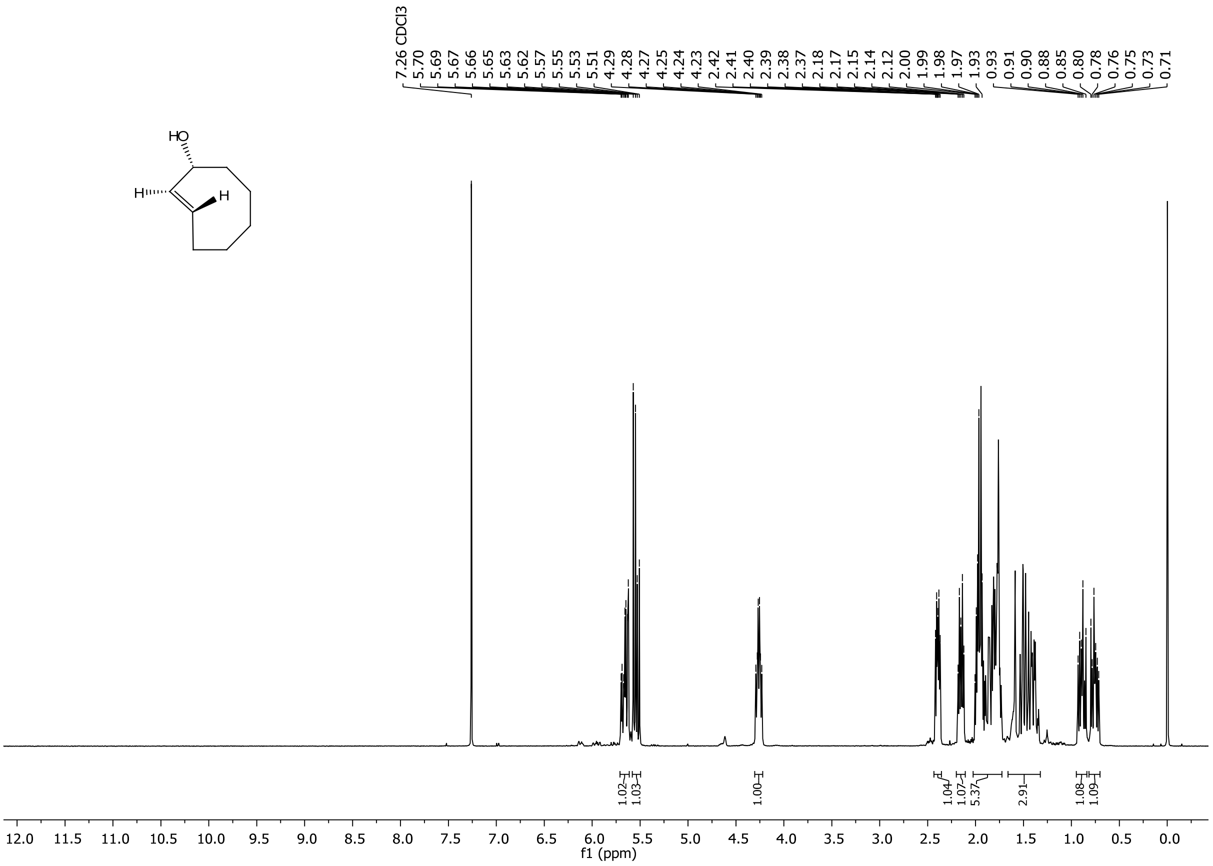

Figure S 23 ^{1}H NMR-spectrum (400 MHz, $CDCl_3$) of **2e.**

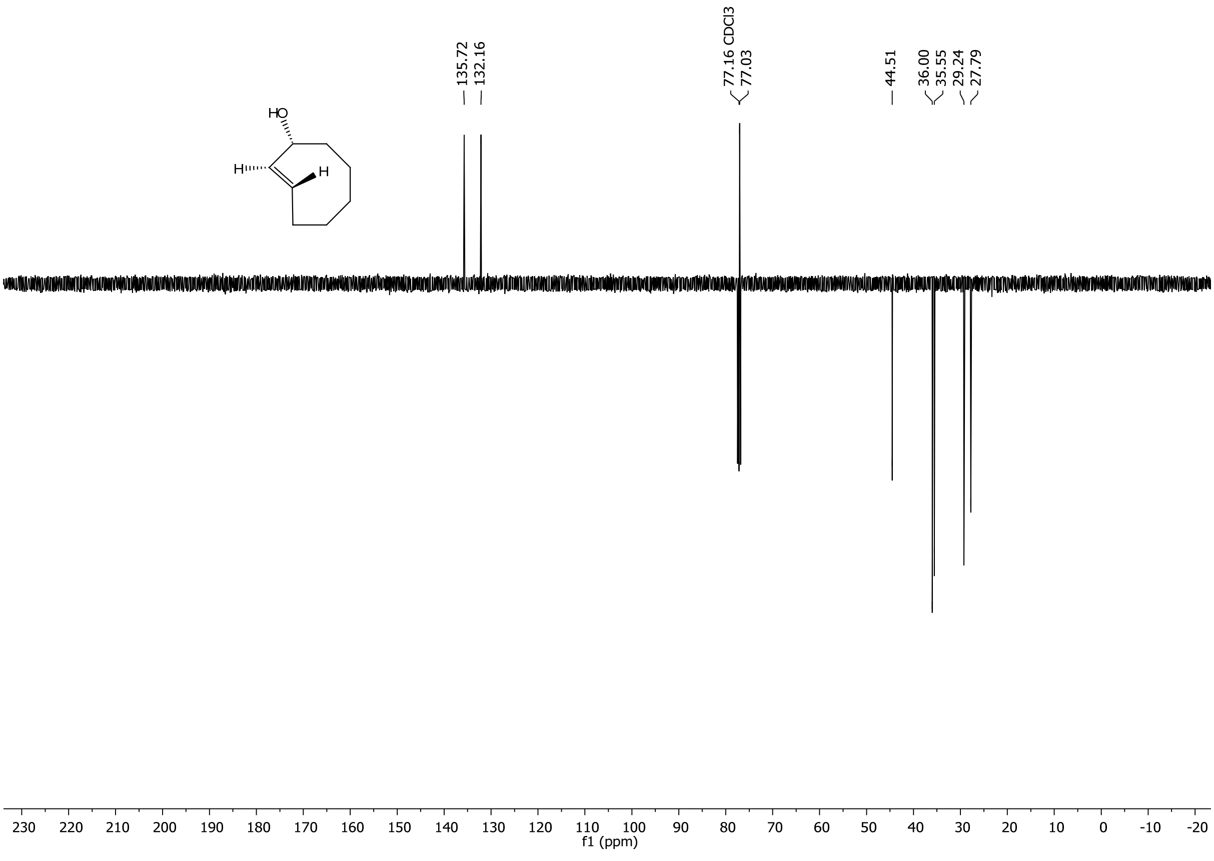

Figure S 24 ^{13}C NMR-spectrum (101 MHz, $CDCl_3$) of **2e.**

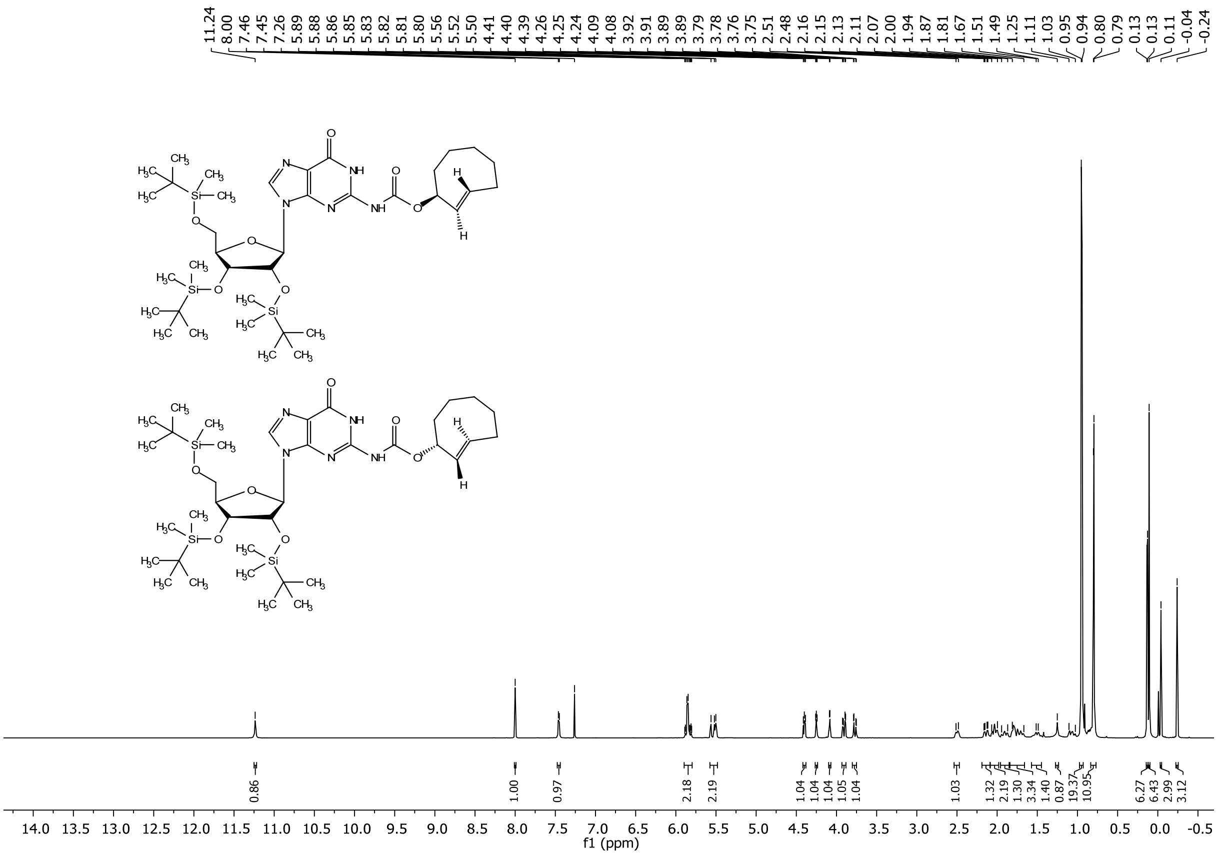

Figure S 25 ^{1}H NMR-spectrum (400 MHz, $CDCl_3$) of **5a.**

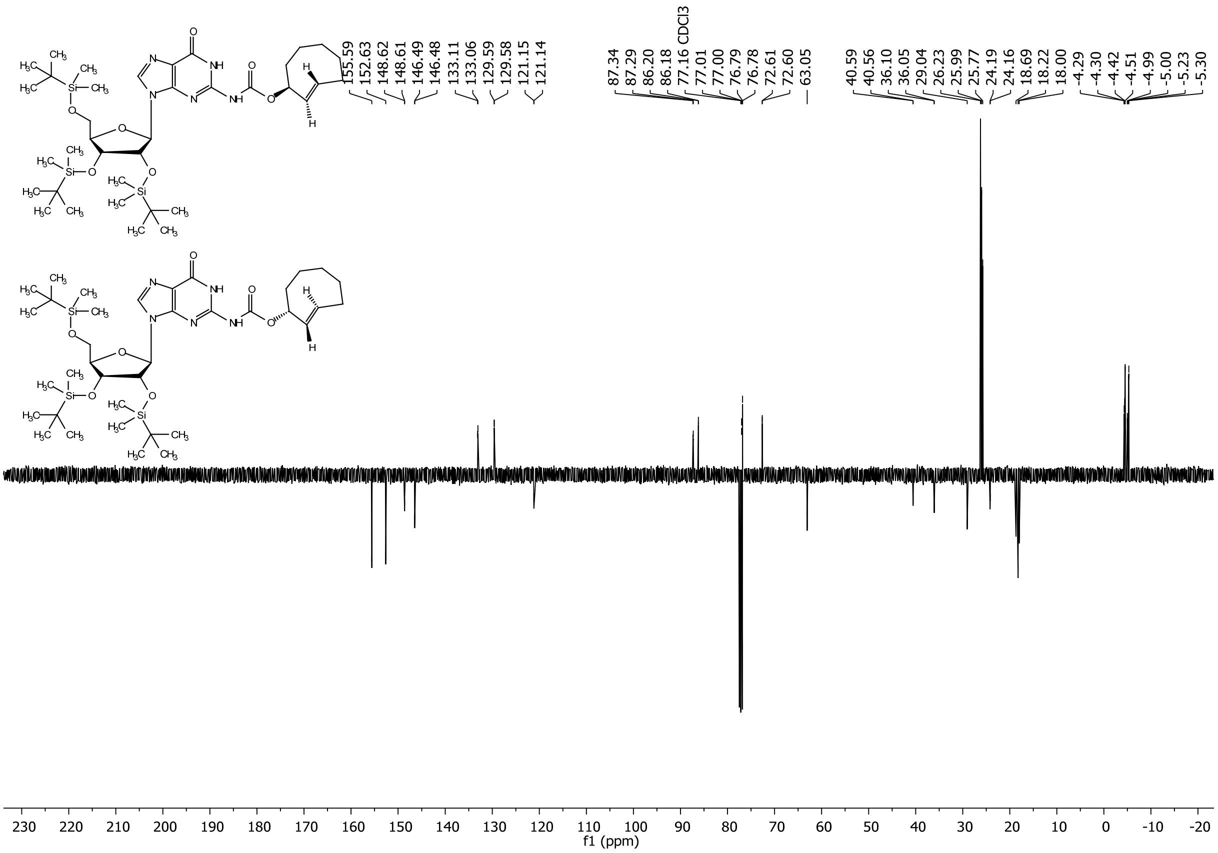

Figure S 26 ^{13}C NMR-spectrum (101 MHz, $CDCl_3$) of **5a**.

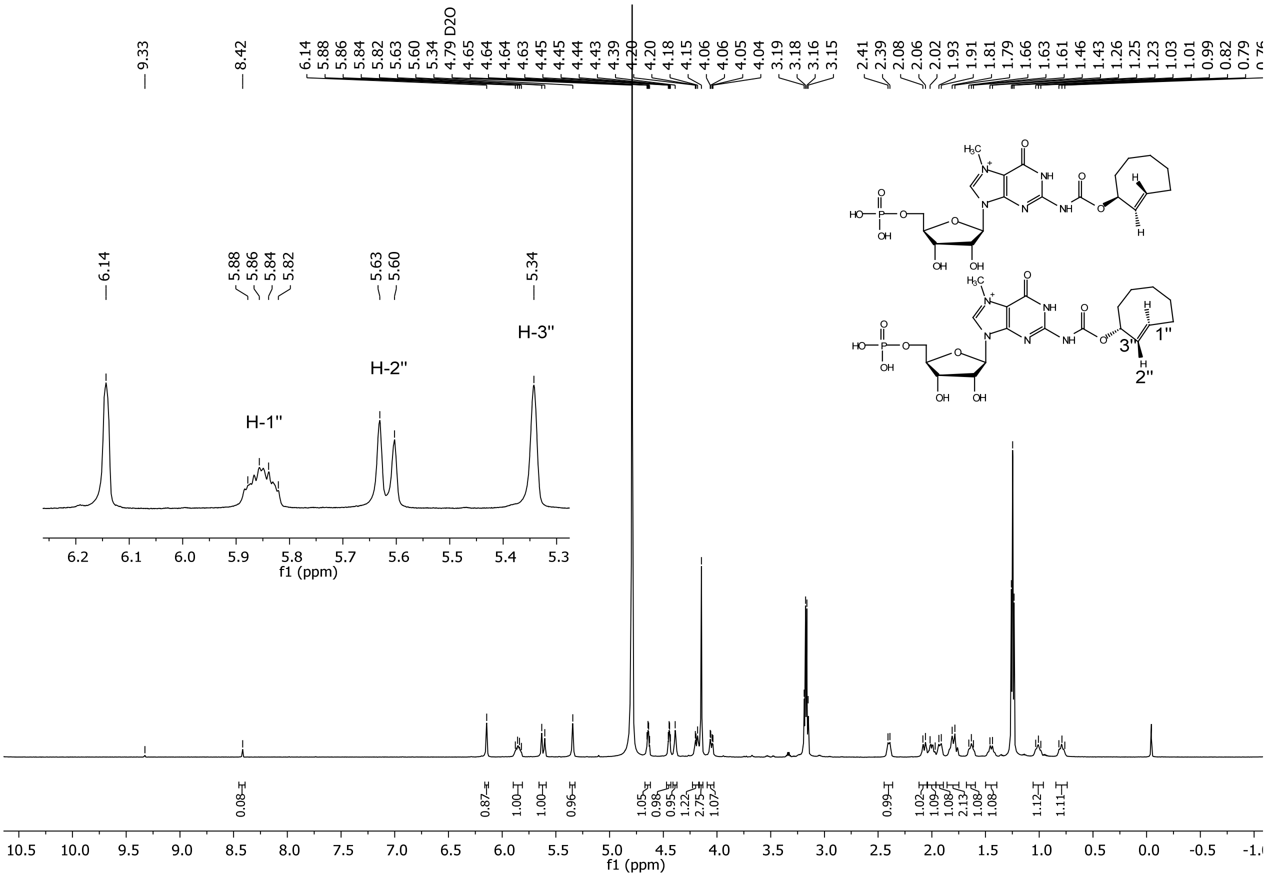

Figure S 27 ^{1}H NMR-spectrum (600 MHz, D_2O) of **7a**.

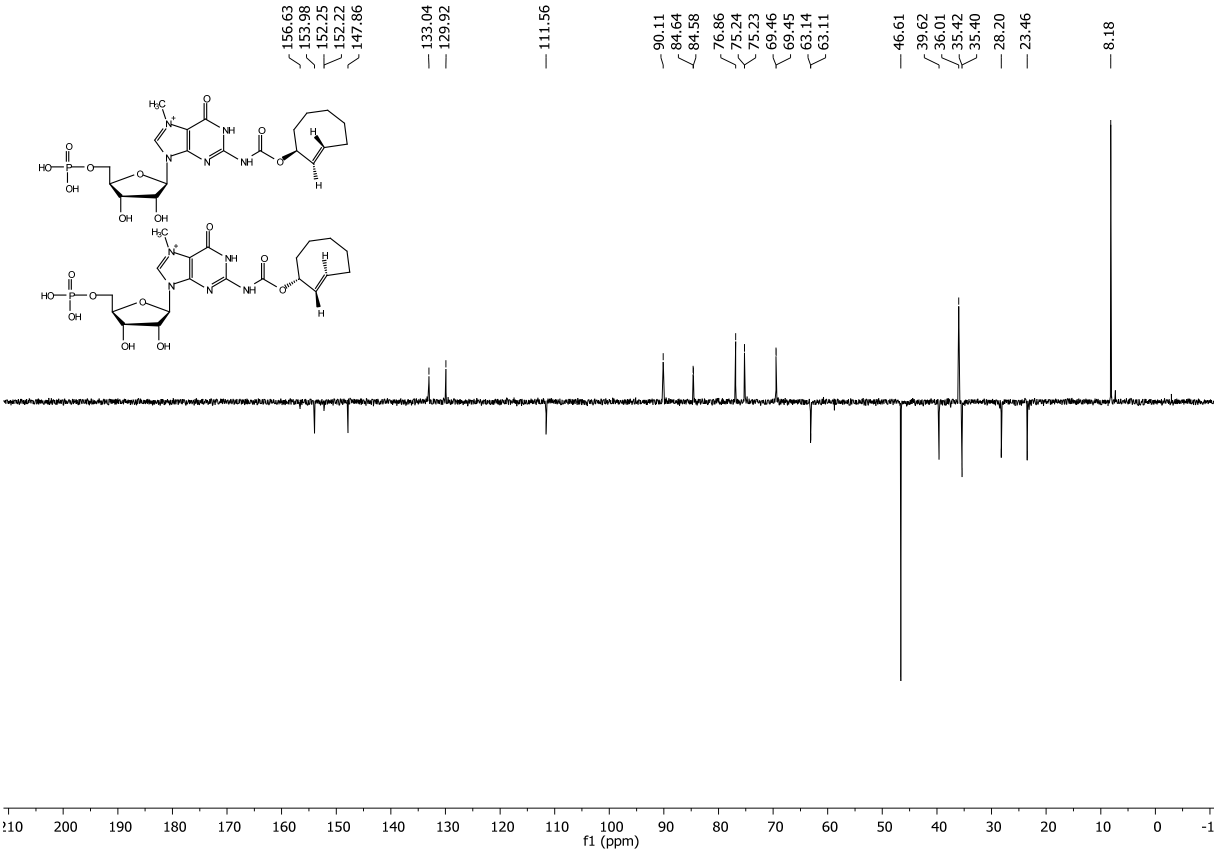

Figure S 28 ^{13}C NMR-spectrum (151 MHz, D_2O) of **7a.**

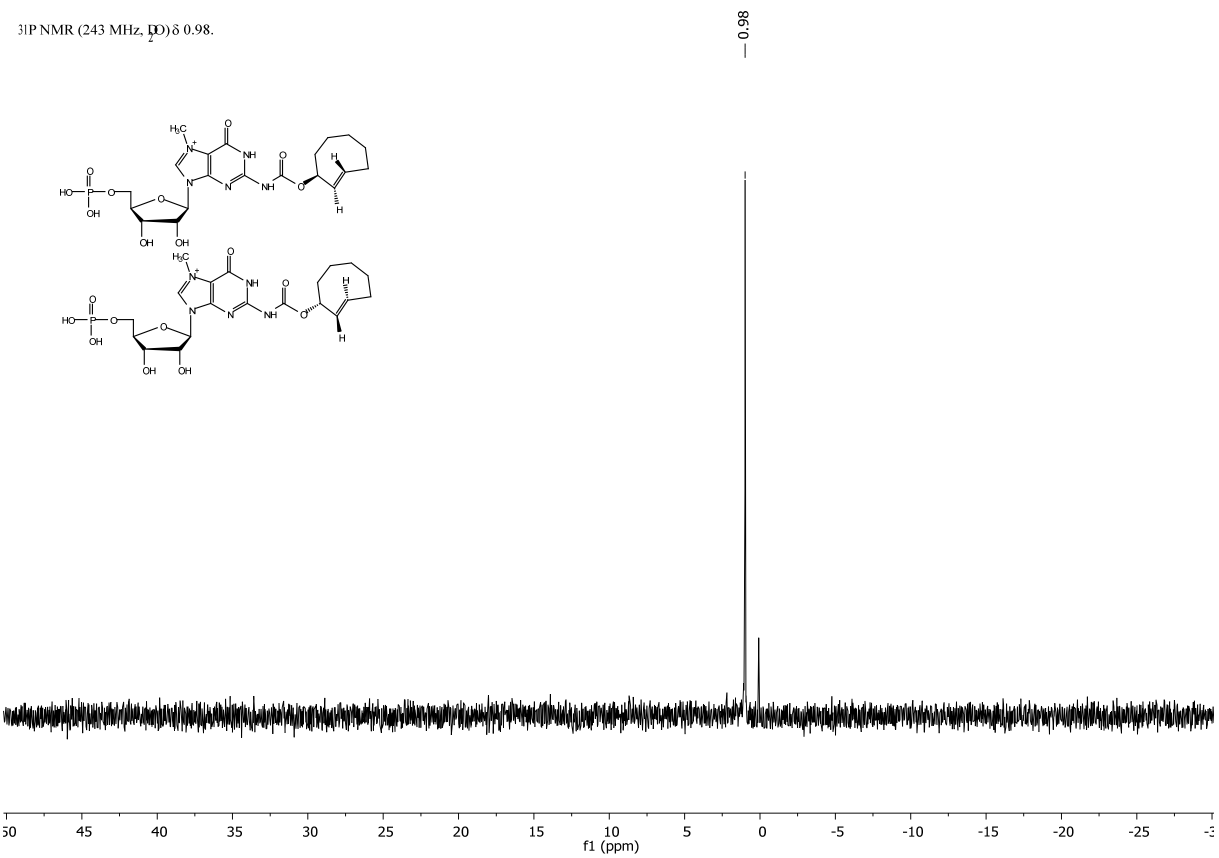

Figure S 29 ^{31}P NMR-spectrum (243 MHz, D_2O, ^{1}H-decoupled) of **7a.**

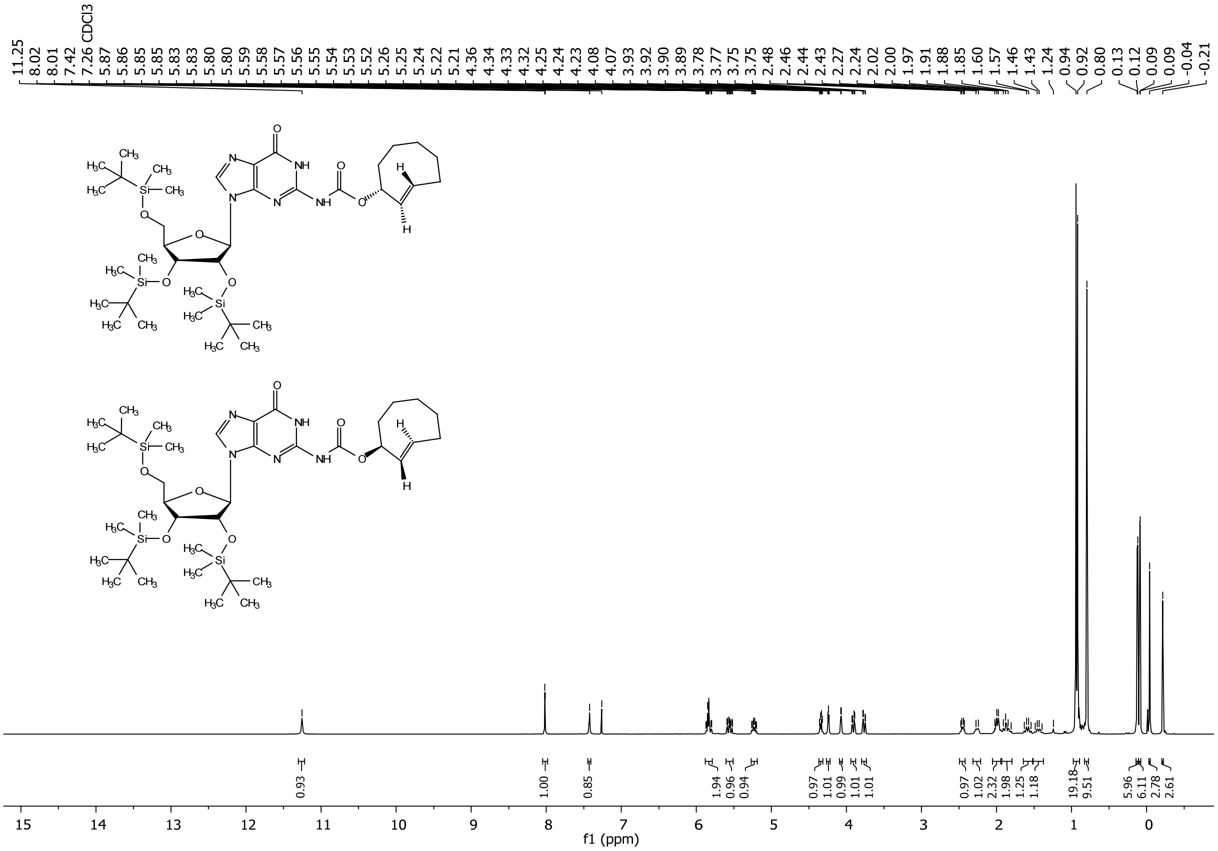

Figure S 30 ^{1}H NMR-spectrum (400 MHz, $CDCl_3$) of **5e**.

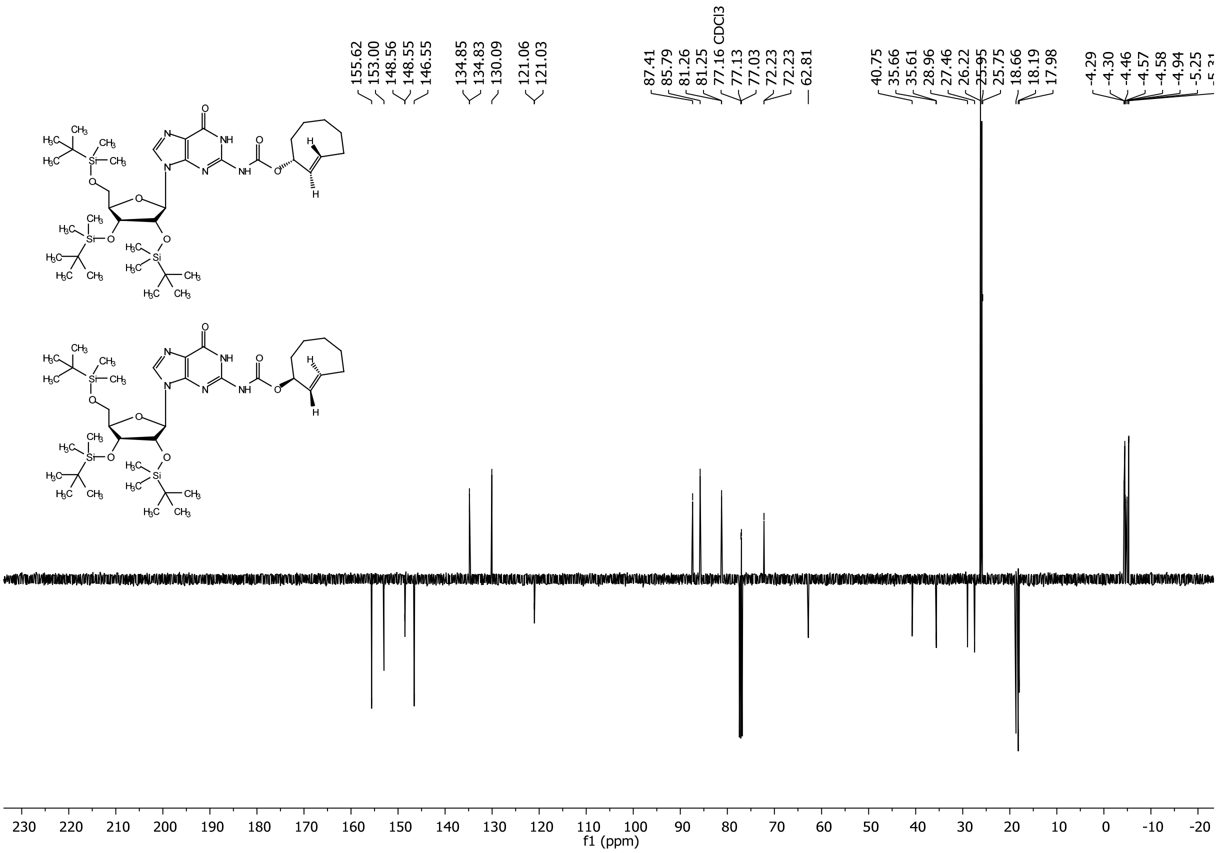

Figure S 31 ^{13}C NMR-spectrum (101 MHz, $CDCl_3$) of **5e**.

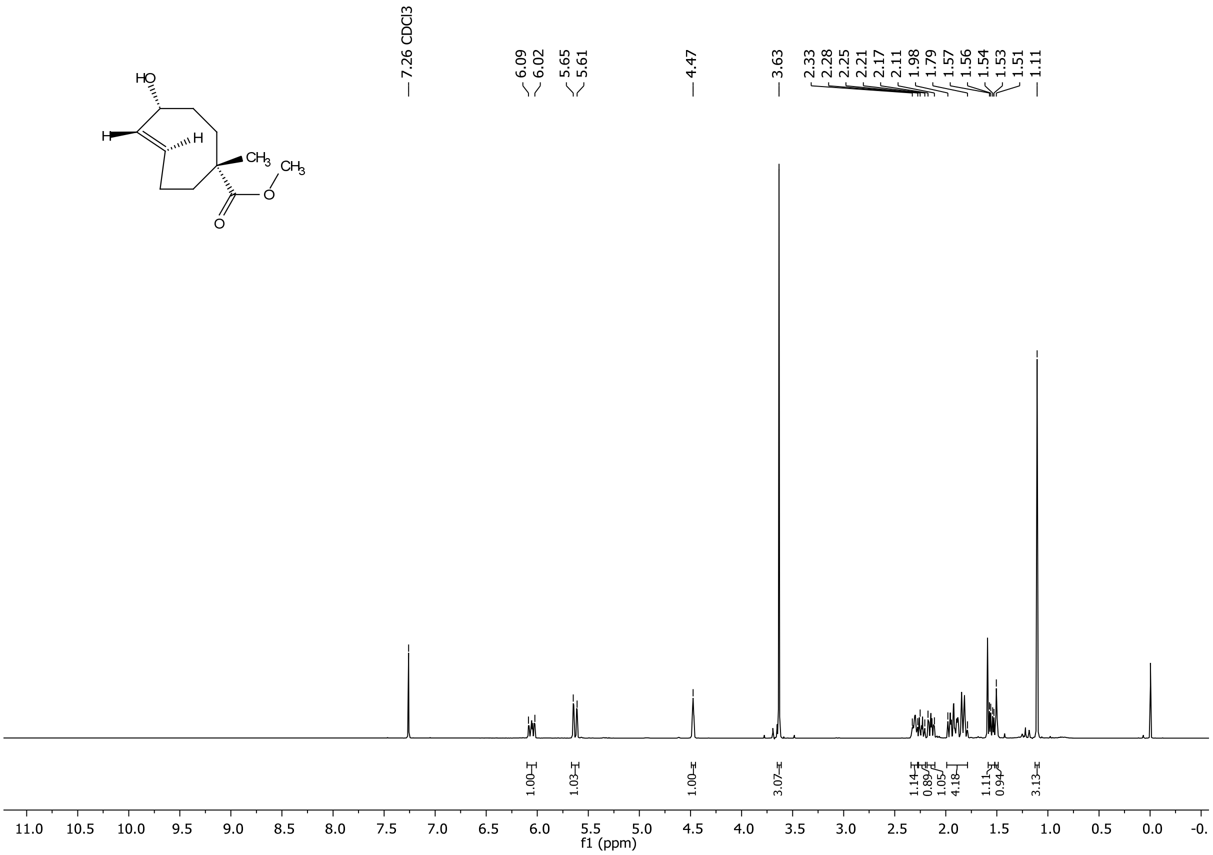

Figure S 32: ^{1}H NMR-spectrum (500 MHz, $CDCl_3$) of **3a.**

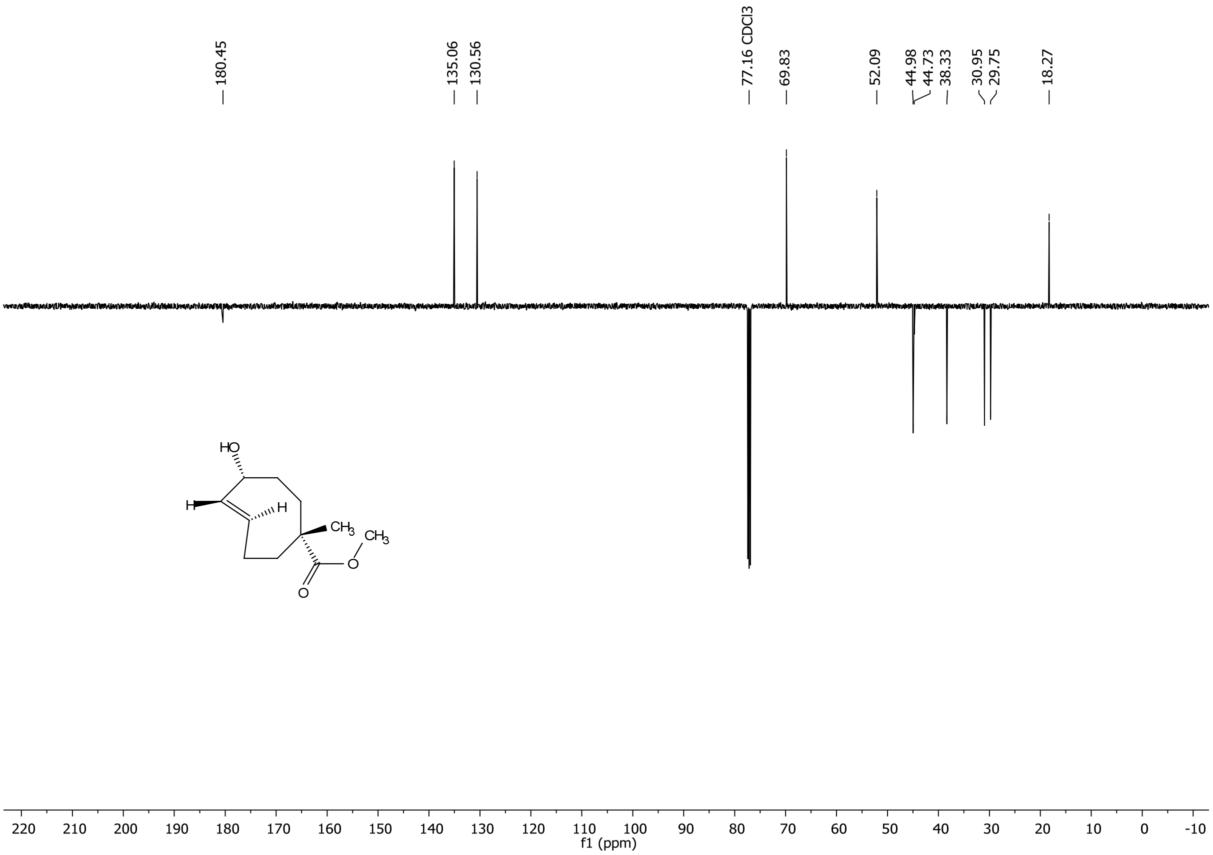

Figure S 33 ^{13}C NMR-spectrum (126 MHz, $CDCl_3$) of **3a.**

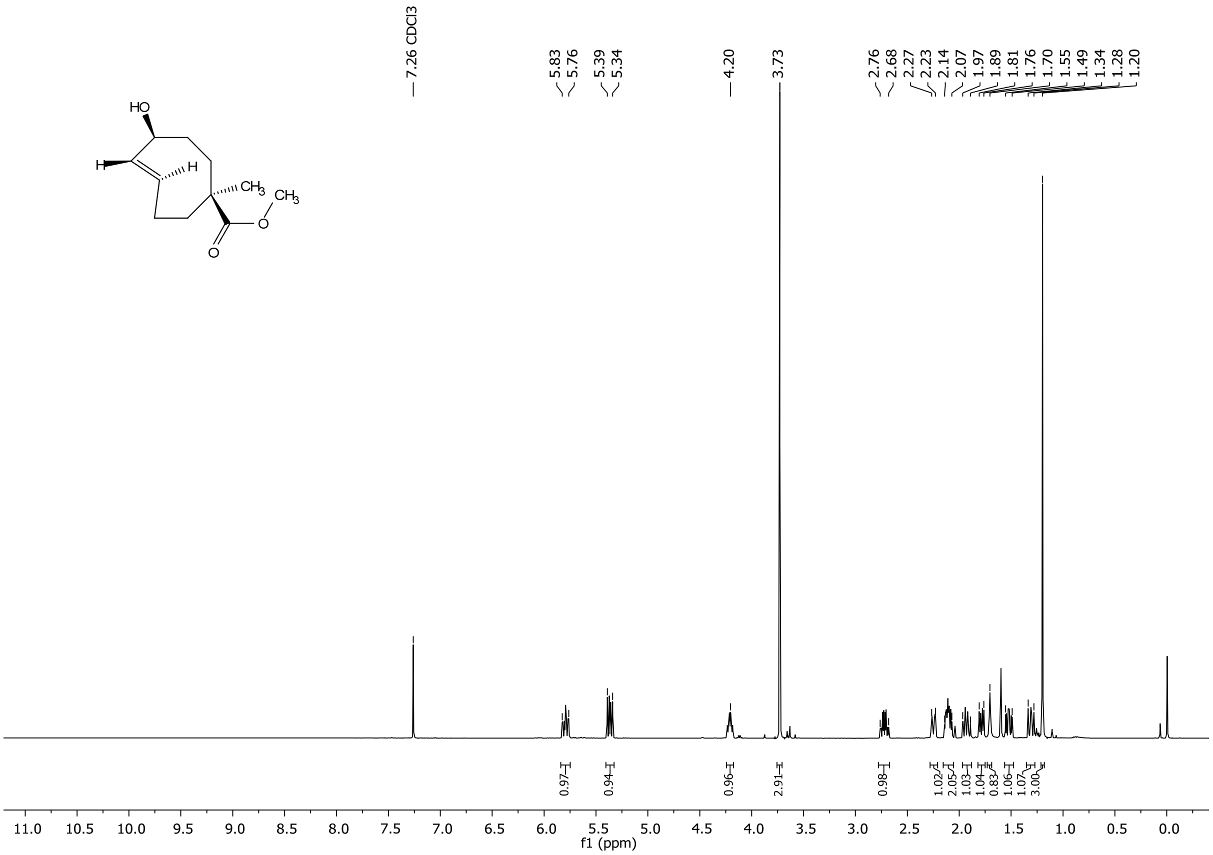

Figure S 34 ^{1}H NMR-spectrum (500 MHz, $CDCl_3$) of **3e**.

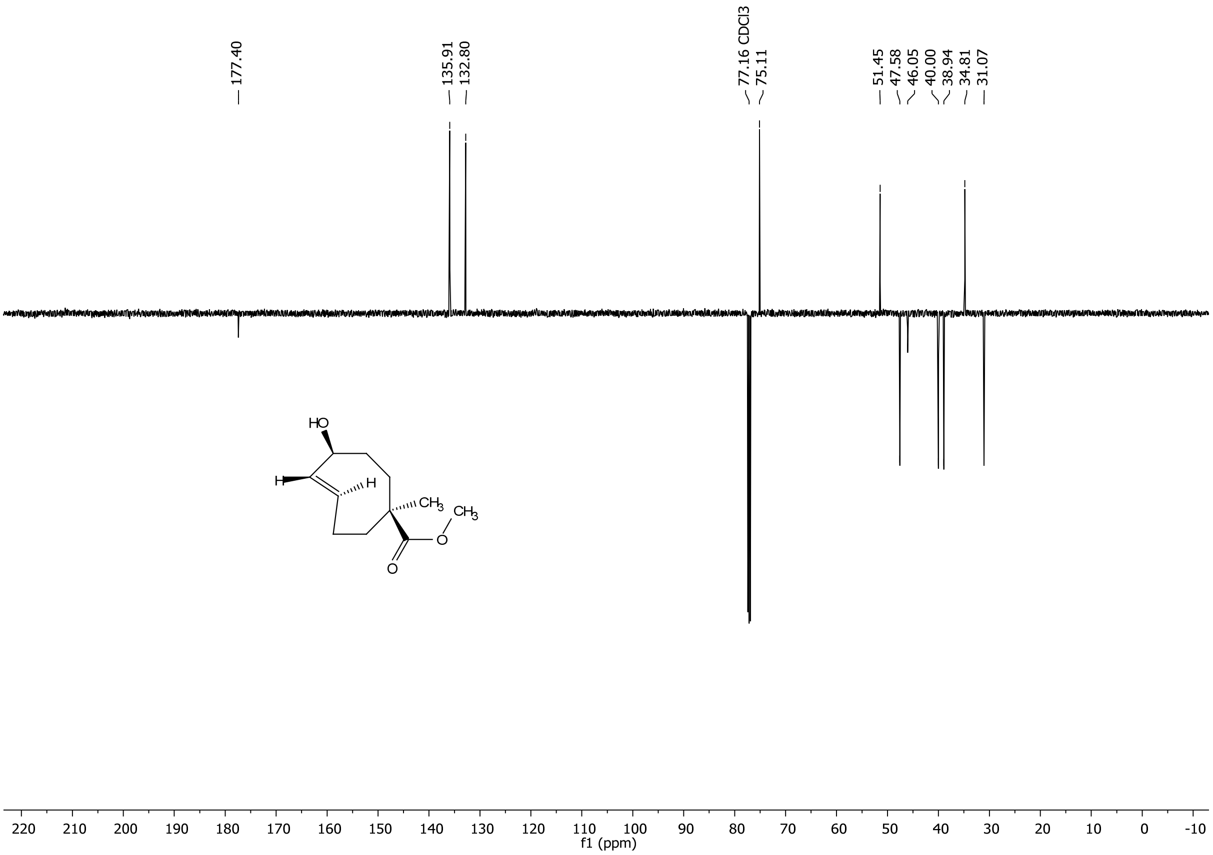

Figure S 35: ^{13}C NMR-spectrum (126 MHz, $CDCl_3$) of **3e**.

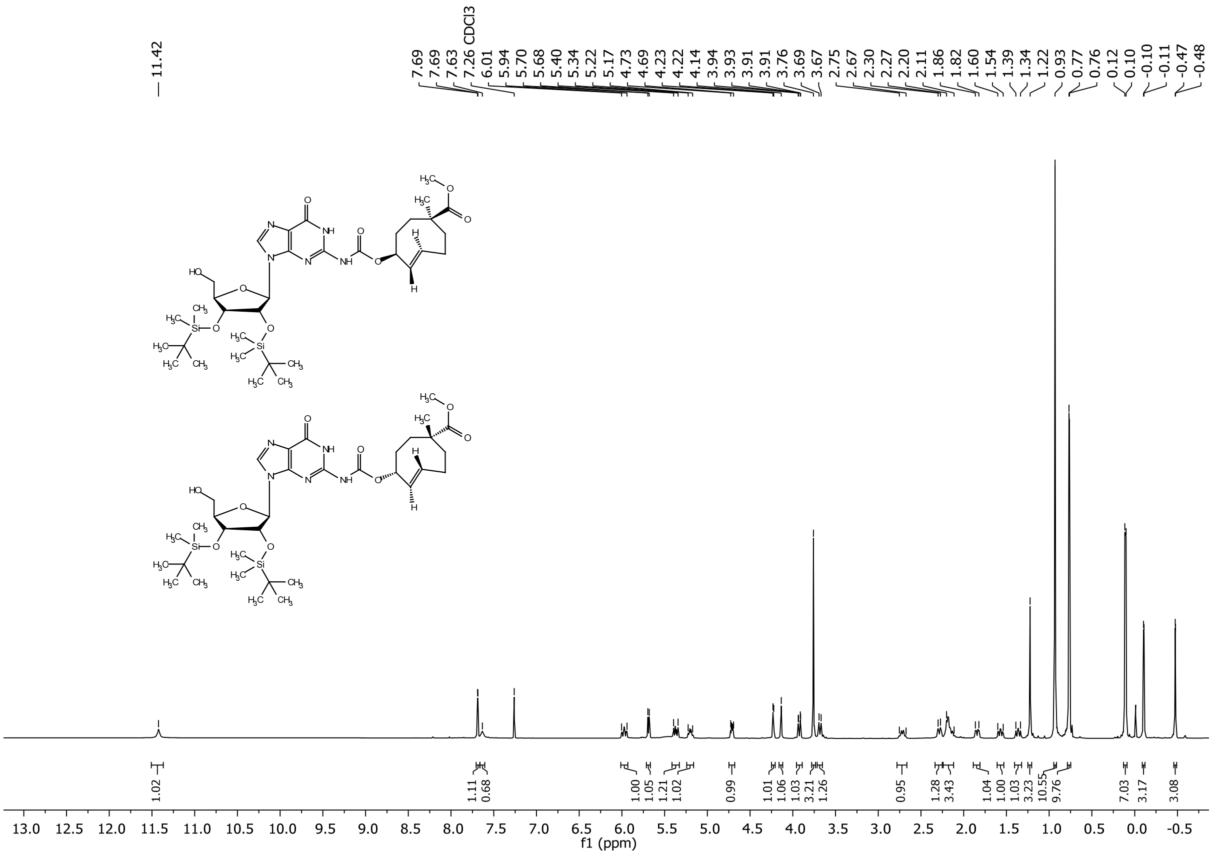

Figure S 36 ^{1}H NMR-spectrum (500 MHz, $CDCl_3$) of **6e.**

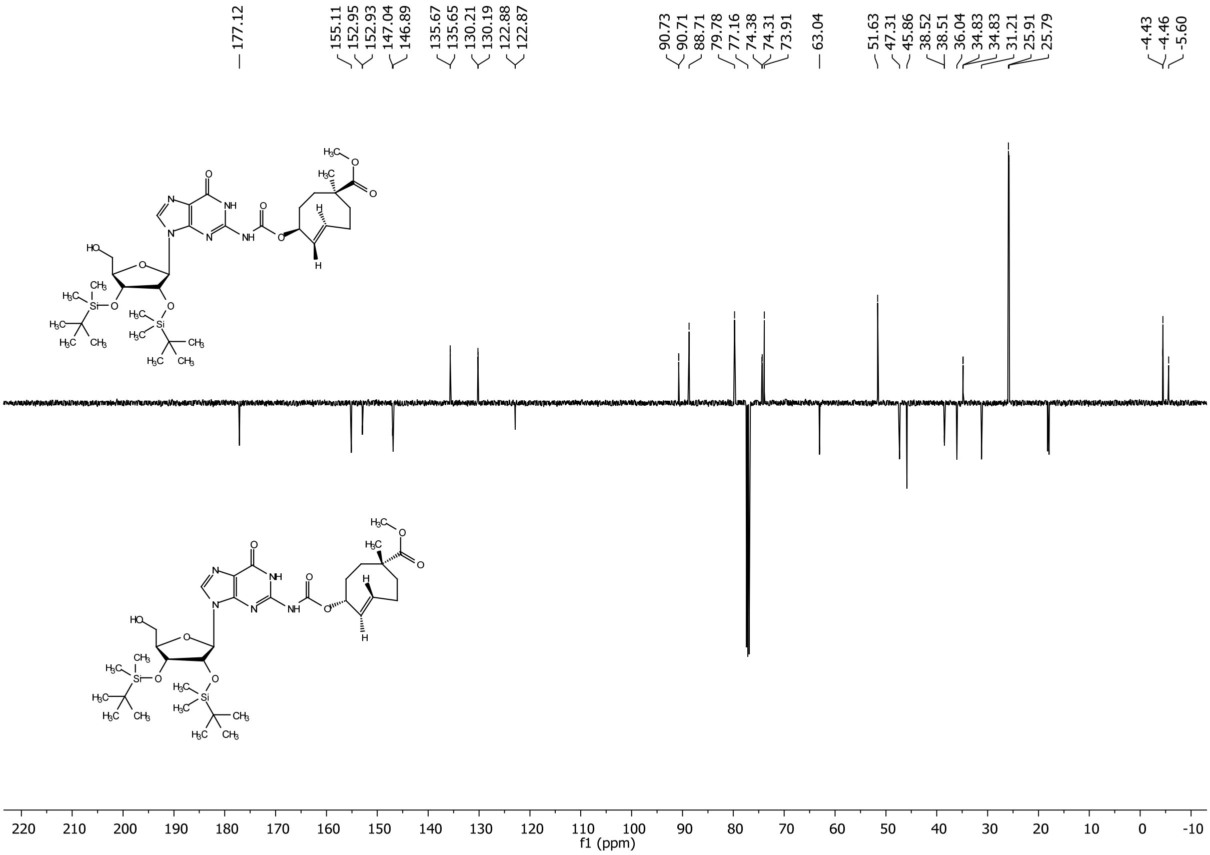

Figure S 37 ^{13}C NMR-spectrum (126 MHz, $CDCl_3$) of **6e**.

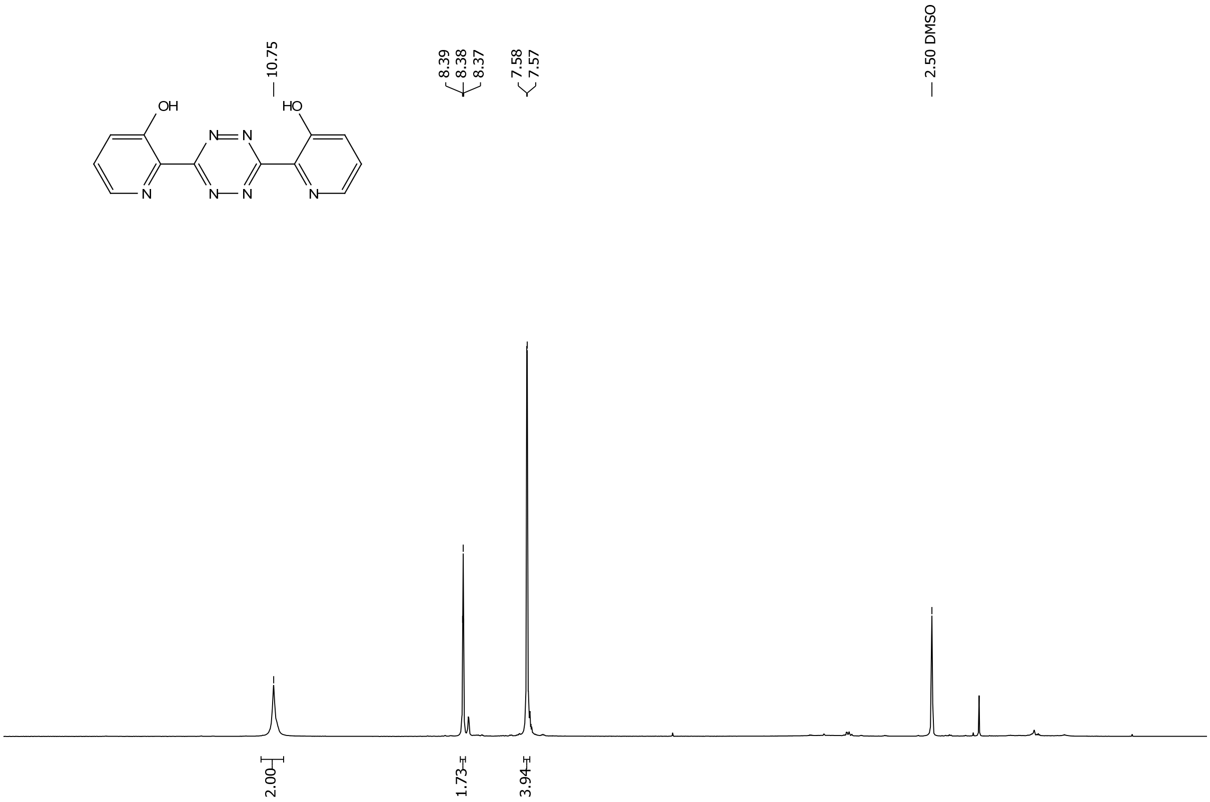

Figure S 38 ^{1}H NMR-spectrum (500 MHz, DMSO_d_6) of **8**.

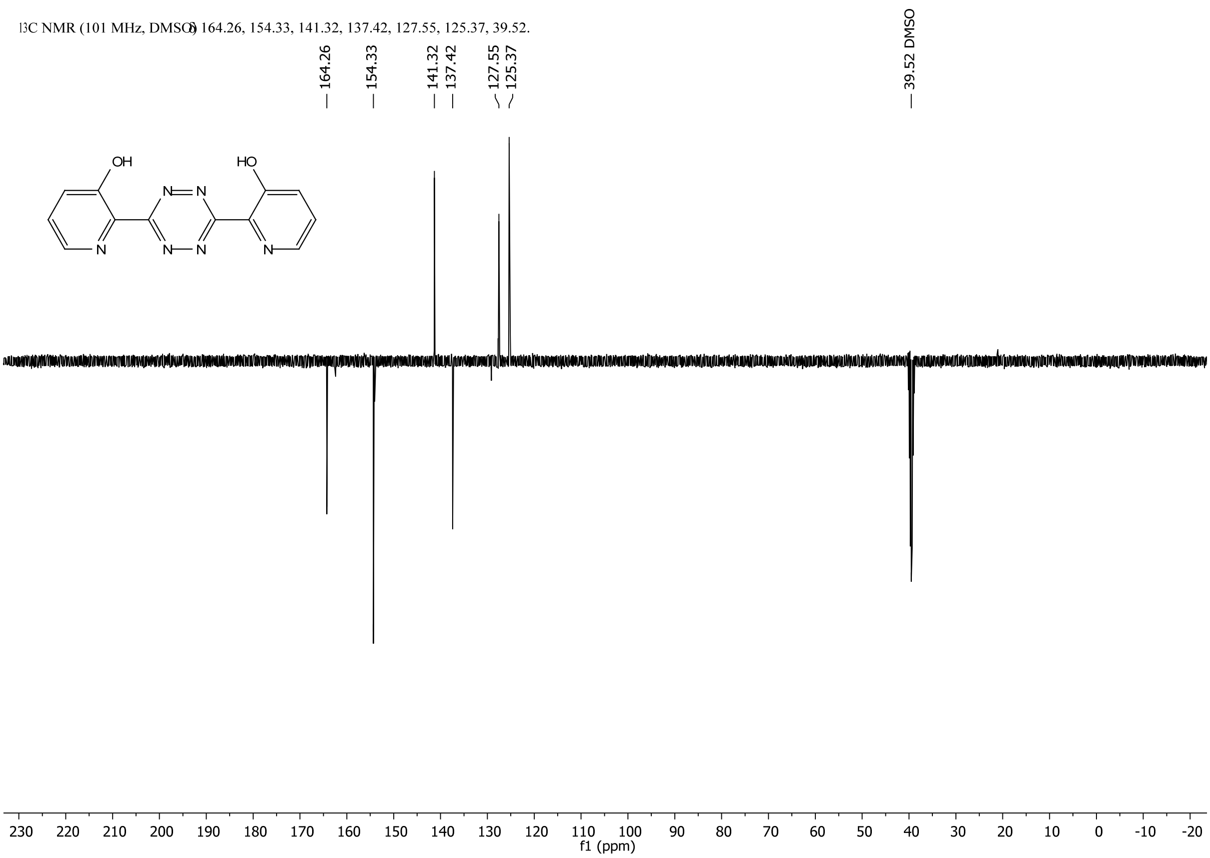

Figure S 39 ^{13}C NMR-spectrum (101 MHz, DMSO_d_6) of 8.

6 References

[4] F. Eggert, S. Kath-Schorr, *Chemical Communications* **2016**, *52*, 7284–7287.

[5] L. Bornewasser, C. Domnick, S. Kath-Schorr, *Chemical Science* **2022**, *13*, 4753–4761.

[8] R. M. Versteegen, R. Rossin, W. ten Hoeve, H. M. Janssen, M. S. Robillard, *Angewandte Chemie International Edition* **2013**, *52*, 14112–14116.

[18] M. Royzen, G. P. A. Yap, J. M. Fox, *Journal of the American Chemical Society* **2008**, *130*, 3760–3761.

[19] D. Svatunek, C. Denk, V. Rosecker, B. Sohr, C. Hametner, G. Allmaier, J. Fröhlich, H. Mikula, *Monatshefte für Chemie - Chemical Monthly* **2016**, *147*, 579–585.

[21] M. Wilkovitsch, W. Kuba, P. Keppel, B. Sohr, A. Löffler, S. Kronister, A. F. del Castillo, M. Goldeck, R. Dzijak, M. Rahm, M. Vrabel, D. Svatunek, J. C. T. Carlson, H. Mikula, *Angewandte Chemie International Edition* **2025**, *64*, e202411707.

[22] M. Rahm, P. Keppel, V. Šlachtová, R. Dzijak, M. Dračínský, S. Bellová, P. E. Reyes-Gutiérrez, S. Štěpánová, J. Raffler, E. Tloušťová, H. Mertlíková-Kaiserová, H. Mikula, M. Vrabel, *Angewandte Chemie International Edition* **2025**, *64*, e202411713.

[24] J. Jemielity, T. Fowler, J. Zuberek, J. Stepinski, M. Lewdorowicz, A. Niedzwiecka, R. Stolarski, E. Darzynkiewicz, R. E. Rhoads, *RNA* **2003**, *9*, 1108–1122.

[25] G. R. Ediriweera, J. D. Simpson, A. V. Fuchs, T. K. Venkatachalam, M. Van De Walle, C. B. Howard, S. M. Mahler, J. P. Blinco, N. L. Fletcher, Z. H. Houston, C. A. Bell, K. J. Thurecht, *Chemical Science* **2020**, *11*, 3268–3280.

[26] J. L. Banks, H. S. Beard, Y. Cao, A. E. Cho, W. Damm, R. Farid, A. K. Felts, T. A. Halgren, D. T. Mainz, J. R. Maple, R. Murphy, D. M. Philipp, M. P. Repasky, L. Y. Zhang, B. J. Berne, R. A. Friesner, E. Gallicchio, R. M. Levy, *Journal of Computational Chemistry* **2005**, *26*, 1752–1780.

[27] L. Schrödinger, Schrödinger, LLC, New York, NY, **2025**.
